# Steric and Geometrical Frustration Generate Two Higher-Order Cu^I^_12_L_8_ Assemblies from a Triaminotriptycene Subcomponent

**DOI:** 10.1021/jacs.3c09547

**Published:** 2024-01-22

**Authors:** Huangtianzhi Zhu, Tanya K. Ronson, Kai Wu, Jonathan R. Nitschke

**Affiliations:** Yusuf Hamied Department of Chemistry, University of Cambridge, Lensfield Road, Cambridge CB2 1EW, U.K.

## Abstract

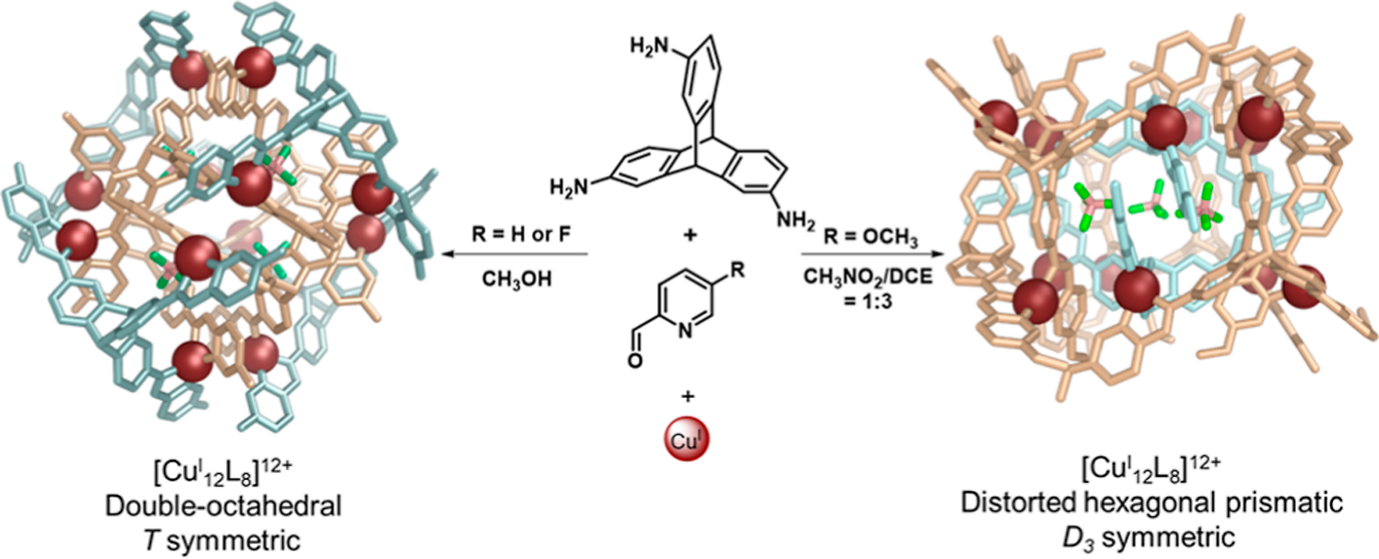

The use of copper(I) in metal–organic assemblies leads readily to the formation of simple grids and helicates, whereas higher-order structures require complex ligand designs. Here, we report the clean and selective syntheses of two complex and structurally distinct Cu^I^_12_L_8_ frameworks, **1** and **2**, which assemble from the same simple triaminotriptycene subcomponent and a formylpyridine around the Cu^I^ templates. Both represent new structure types. In *T*-symmetric **1**, the copper(I) centers describe a pair of octahedra with a common center but whose vertices are offset from each other, whereas in *D*_3_-symmetric **2**, the metal ions form a distorted hexagonal prism. The syntheses of these architectures illustrate how more intricate Cu^I^-based complexes can be prepared via subcomponent self-assembly than has been possible to date through consideration of the interplay between the subcomponent geometry and solvent and electronic effects.

## Introduction

Self-assembly enables the formation of organized, complex structures, as reversibly formed linkages bring simpler components together during thermodynamic equilibration, affording diverse and functional structures and systems.^1^ Self-assembly driven by metal coordination provides an efficient approach to constructing polyhedral metal–organic complexes.^2^ These products have found useful applications in a variety of fields, including guest-specific recognition,^3^ delivery of biomacromolecules,^4^ adsorption and separation,^5^ control of reactivity,^6^ luminescent systems,^7^ and polymeric materials.^8^

An increasing number of these metal–organic self-assembled structures are prepared using subcomponent self-assembly, whereby reversible covalent (usually C=N) and coordinative (N→Metal) bonds are formed during the same overall process.^9^ In most cases, the transition metal templates used have octahedral coordination geometries, such as Fe^II^ and Zn^II^, and the ligands are iminopyridines, with each metal center bringing three such ligands together into a tightly constrained linking unit within a larger superstucture.^10^ Tetrahedral Cu^I^, in contrast, joins only two iminopyridine ligands in a less constrained junction and thus tends to serve as a more flexible linker than the octahedral metals. The generation of more complex self-assembled structures using Cu^I^ is made challenging by this flexibility. Copper(I) thus tends to favor lower-nuclearity structures such as helicates and grids,^11^ with larger structures requiring intricate ligand design,^12^ or careful steric tuning so as to dictate heteroleptic complex formation, such as the intricate architetures reported by Schmittel’s group,^13^ and the earlier cylindrical nanostructures^14^ and grids^15^ reported by Lehn et al.

Because copper(I) structures possess useful features, including photoluminescence,^16^ redox behavior,^17^ and stability in aqueous media,^18^ it is a worthwhile goal to generate increasingly complex host structures using Cu^I^, which would be capable of binding large and information-rich guest species.

**Figure 1:**
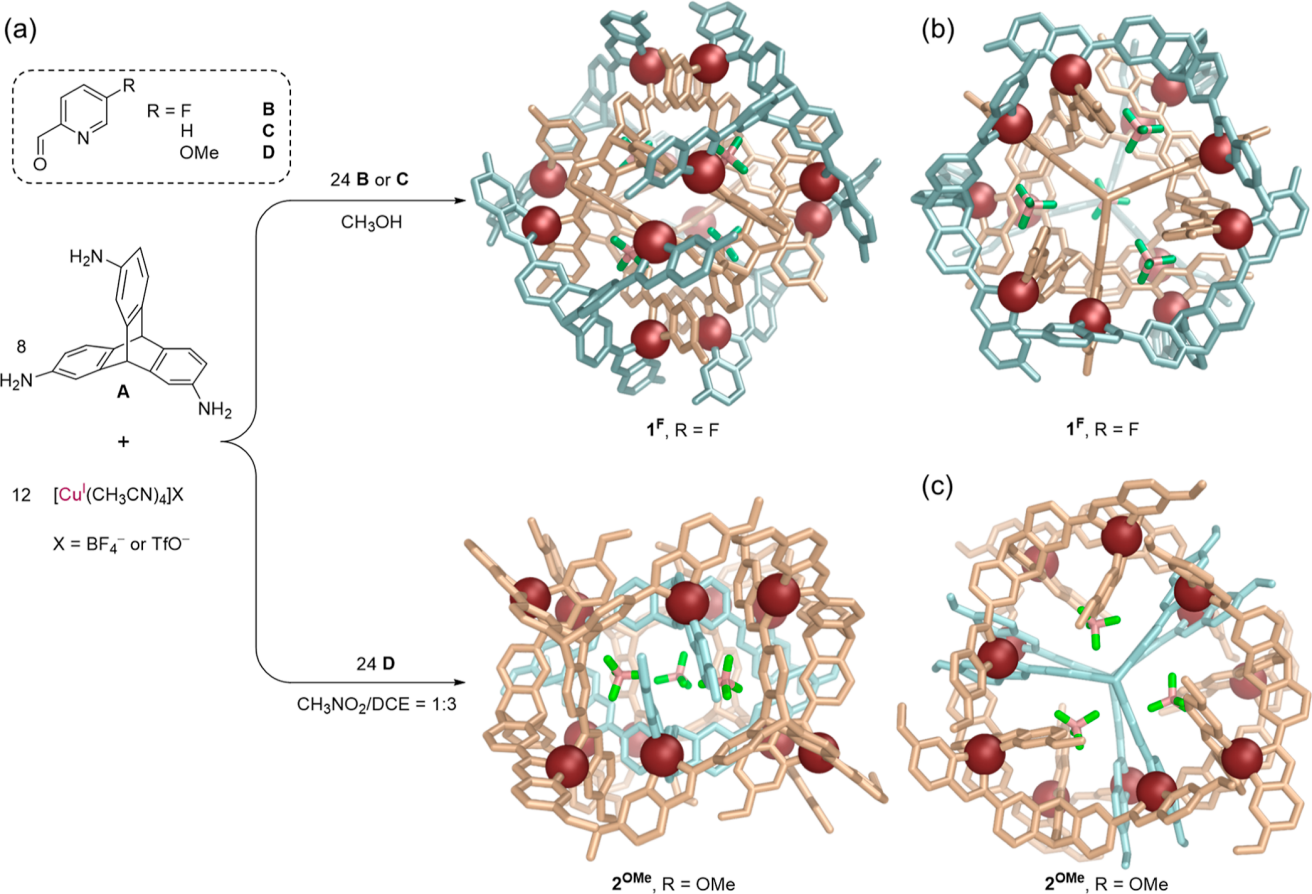
(a) Selective assembly of two distinct Cu^I^_12_L_8_ frameworks **1** and **2** using different solvents and differently substituted formylpyridine derivatives, and views down the *C*_2_ symmetry axes of the crystal structures of **1**^**F**^ and **2**^**OMe**^, where superscripts refer to the 5-substituents on the formylpyridine subcomponent used to make either framework **1** or **2**. (b) and (c) views down the *C*_3_ axes of the crystal structures of **1**^**F**^ and **2**^**OMe**^, respectively. Hydrogen atoms, counteranions except for bound BF_4_^–^, solvent molecules, and disorder are omitted for clarity. Internal and external ligands are individually colored.

We hypothesized that a simple ligand that incorporated the key features of steric hindrance and curvature might be capable of preventing the face-to-face stacking of pyridylimine ligands during subcomponent self-assembly around Cu^I^ templates, affording novel architectures. Triaminotriptycene **A** (Figure 1) exhibits curvature and rigidity,^19^ and we anticipated that its C–H groups positioned between the amino groups and the triptycene bridgehead would generate a steric clash that might preclude the formation of simpler, lower-nuclearity assemblies. Triamine **A** indeed assembled with 2-formylpyridines and copper(I) to form two large and distinct Cu^I^_12_L_8_ assemblies, *T*-symmetric **1** and *D*_3_-symmetric **2** (Figure 1). These two Cu^I^_12_L_8_ assemblies each represent a new structure type.

## Results and Discussion

Triamine **A** (8 equiv) reacted with 5-fluoro-2-formylpyridine (**B**, 24 equiv) and tetrakis(acetonitrile)copper(I) tetrafluoroborate (Cu^I^(CH_3_CN)_4_BF_4_, 12 equiv) in methanol at 343 K to produce product **1**^**F**^ (Figure 1), where the superscripted “**F**” denotes the 5-substituent on the formylpyridine subcomponent. ESI-MS (Figure S30) indicated a Cu^I^_12_L_8_ formulation. We infer that steric hindrance at the central triptycene panel and ligand curvature aid the formation of the complex Cu^I^ structure by preventing face-to-face stacking of pyridylimine ligands during subcomponent self-assembly, thus preventing the formation of smaller assemblies.

**Figure 2:**
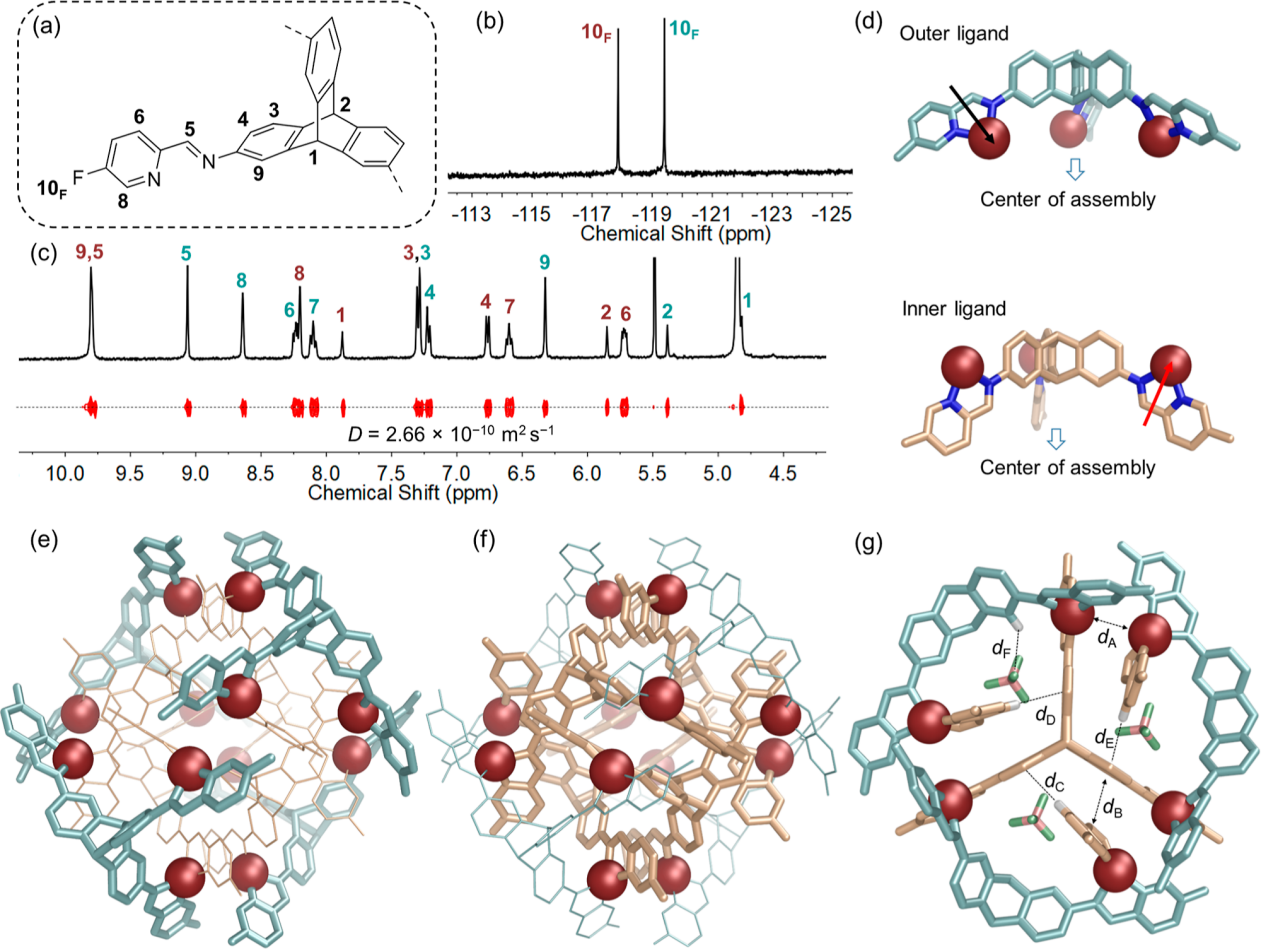
(a) Partial structure of the ligand within **1**^**F**^, showing the labeling scheme. (b) Partial ^19^F NMR spectrum (471 MHz, 298 K, CD_3_OD) of **1**^**F**^. (c) Partial ^1^H NMR and DOSY spectra (400 MHz, 298 K, CD_3_OD) of **1**^**F**^, with two sets of signals labeled to correspond to the numbers in (a) and colored in light cyan and ruby, respectively. (d) Two distinct types of ligands are observed within the X-ray crystal structure of **1**^**F**^, with an inward-facing coordination vector shown in black and an outward-facing vector shown in red. (e) and (f) Crystal structure of **1**^**F**^ viewed down the *C*_2_ axis, with Cu^I^ in ruby; the outer ligands, emphasized in E, are rendered in light cyan, and the inner ligands, emphasized in F, are shown in wheat. Hydrogen atoms, anions, solvents, and disorder are omitted for clarity. (g) Partial view of the crystal structure of **1**^**F**^ down a *C*_3_ axis, where *d*_A_ shows the 4.53 Å distance between two Cu^I^ centers, *d*_B_ gives the 3.90 Å spacing between the centroids of nearest-neighbor aromatic rings, *d*_C_, *d*_D_, and *d*_E_ show the 2.66 Å C–H···π interactions inferred to stabilize **1**^**F**^, and *d*_F_ is one of the C–H···F interactions involved in anion binding. Hydrogen atoms are white; fluorine and boron in BF_4_^–^ are green and pink, respectively.

The ^1^H NMR spectrum of **1**^**F**^ in methanol was complex yet well-resolved. The ^1^H, ^13^C, ^19^F, and ^1^H diffusion-ordered spectroscopy (^1^H DOSY) and 2D NMR spectra are shown in Supporting Information Section 3 and Figure 2. Transferring **1**^**F**^ that had been prepared in methanol into acetonitrile caused disassembly, and **1**^**F**^ could not be prepared in acetonitrile, nitromethane, or DMSO. We infer that the poor solubility of the triptycene backbone in methanol, together with the high polarity of methanol, drive the formation of **1**, consistent with the multiple noncovalent interactions between building blocks that are observed in the structure (see above).^20^ The same framework could be prepared using the parent 2-formylpyridine **C** instead of **B**, which generated a structure designated **1**^**H**^ instead of **1**^**F**^, but the fluorine atoms of **1**^**F**^ aided in its characterization, as noted below.

The ^19^F NMR spectrum of **1**^**F**^ displayed two signals, assigned to the fluorine substituents on the pyridine rings, with an integration ratio of 1:1, suggesting the presence of two magnetically distinct ligand environments (Figure 2b). Encapsulated BF_4_^–^ and free BF_4_^–^ were both also found. The ^1^H NMR spectrum of **1**^**F**^ was assigned using different 2D NMR techniques, which revealed two distinct imine signals and four resonances assigned to the bridgehead C–H groups of triptycene (Figures 2c and S25). The ^1^H–^19^F HMBC spectrum also confirmed peak assignments (Figure S29). The NMR spectra of **1**^**F**^ contained two sets of magnetically distinct ligands in a 1:1 ratio based on integration, which is in line with the ^19^F NMR results. All peaks exhibited the same ^1^H DOSY diffusion coefficient, indicating that they belonged to a single species (Figure 2c).

Vapor diffusion of diethyl ether into a methanol solution of **1**^**F**^ afforded single crystals suitable for single-crystal X-ray diffraction using synchrotron radiation. The crystal structure of **1**^**F**^ revealed an unprecedented [Cu_12_L_8_]^12+^ superstructure, containing 12 identical Cu^I^ vertices and two different ligand environments, as observed in solution.

All 12 Cu^I^ vertices possess the same handedness in each cage, with the enantiomers of **1**^**F**^ related by inversion in the crystal. The tetrahedral coordination geometry of each Cu^I^ center is completed by one inward-facing ligand and one outward-facing ligand. The midpoints of the pairs of closest-spaced Cu^I^ centers describe the vertices of an octahedron, with an average Cu^I^···Cu^I^ distance (Figure 2g, *d*_A_) of 4.53 ± 0.10 Å.

The structure of **1**^**F**^ contains two distinct ligand environments, facing outside and inside (Figure 2, colored cyan and tan, respectively). Each ligand occupies one of the *C*_3_ symmetry axes that generate the *T* point symmetry of **1**^**F**^ together with *C*_2_ axes (Figure 2e,f) that pass between the closest-spaced pairs of Cu^I^ centers. The coordination vectors of the outer ligands point toward the center of the assembly, whereas the coordination vectors of the inner ligands point out from the center (Figure 2d). These two different ligand environments give rise to two sets of peaks in the ^1^H NMR spectrum.

Within **1**^**F**^, the centroid-to-centroid distances (Figure 2g, *d*_B_) between the triptycene phenyl rings and the pyridine rings are 3.90 ± 0.19 Å, outside the range of effective arene stacking. Analysis of the distance (Figure 2g, *d*_C_) between pyridyl hydrogen atoms and the centroids of triptycene phenyl rings reveals multiple C–H···π interactions, with an average distance of 2.66 ± 0.04 Å and an average angle of 144.3° ± 1.3°. The assembly contains 24 such C–H···π interactions, which are inferred to help stabilize this compact and highly ordered architecture. The solvophobic effect is also implicated in holding the structure of **1**^**F**^ together, as this structure is only stable in methanol, whereas its building block, triptycene, is sparingly soluble in only this solvent.

**Figure 3:**
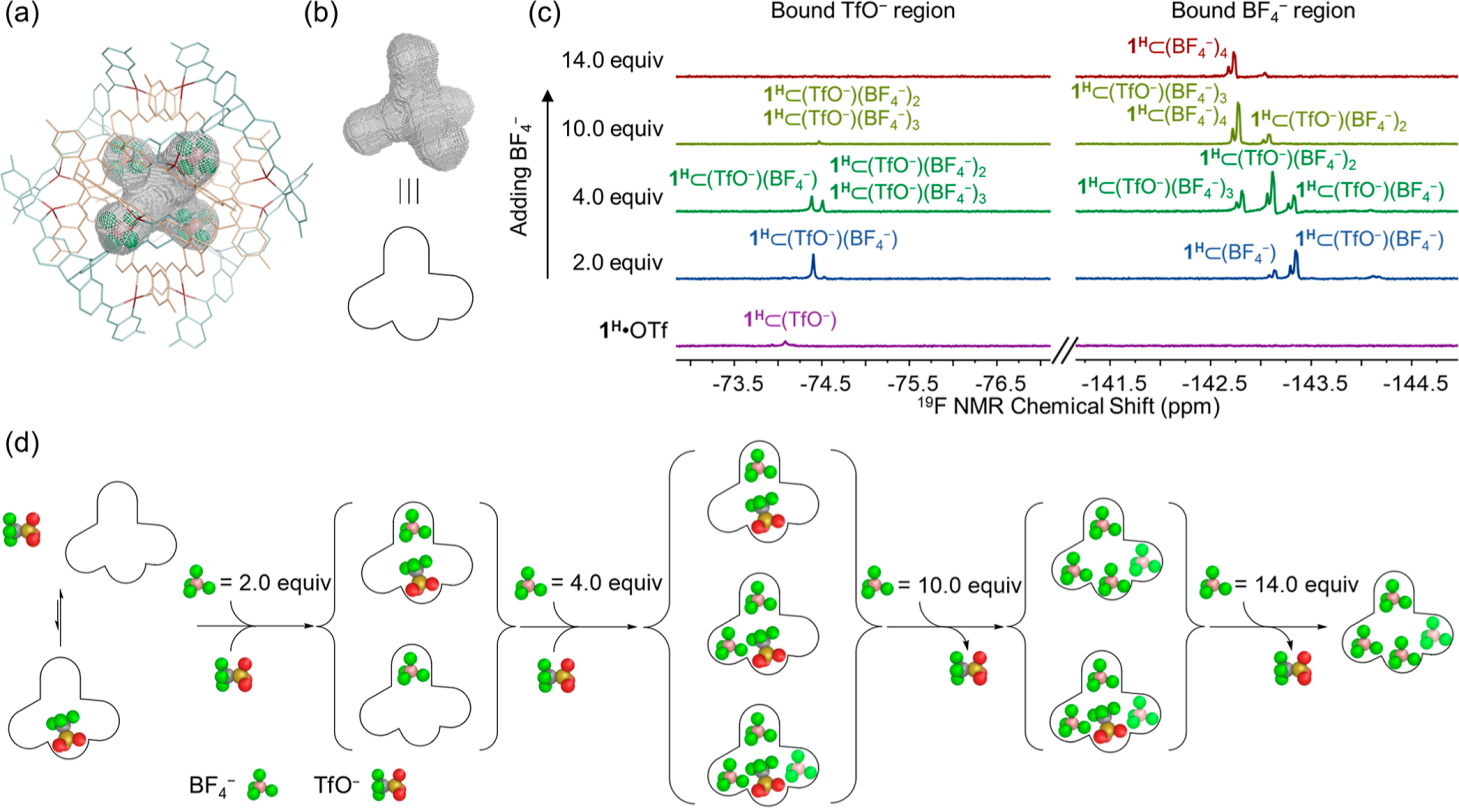
(a) Cavity of **1** outlined in gray mesh based on the X-ray crystal structure of **1**^**F**^·BF_4_. Four encapsulated BF_4_^–^ anions are shown in ball and stick mode. (b) Cavity of **1** taken from **a** and its simplified representation. (c) Partial ^19^F NMR spectra (376 MHz, 298 K, CD_3_OD) of **1**^**H**^ during the addition of TBABF_4_. (d) Schematic representation of the allosterically cooperative binding and subsequent competitive binding of **1**^**H**^ upon the titration of TBABF_4_.

The structure of **1**^**F**^ also includes four BF_4_^–^ anions, consistent with the slow exchange of BF_4_^–^ observed in the ^19^F NMR spectrum. These bound BF_4_^–^ anions each occupy a small, well-enclosed cavity within **1**^**F**^; these cavities connect in a tetrahedral arrangement (Figures 3a and S60). C–H···F hydrogen bonds (Figure 2g, *d*_F_) were observed between the BF_4_^–^ anions and triptycene hydrogen atoms; we infer these interactions to be strengthened by attraction between the complementary charges of the cationic framework of **1**^**F**^ and the anions.^21^

Although BF_4_^–^ matches the sizes of the cavities within framework **1** and undergoes C–H···F hydrogen bonding, this anion is not required to template the formation of **1**. The same framework was prepared by using tetrakis(acetonitrile)copper(I)triflate (Cu^I^(CH_3_CN)_4_OTf) in place of the tetrafluoroborate. Comparison of ^19^F NMR spectra of the triflate and tetrafluoroborate salts of **1** indicated that BF_4_^–^ was bound more strongly than TfO^–^ (Figure S37), however, suggesting that BF_4_^–^ fits better than TfO^–^ within the cavities of **1**.

When both BF_4_^–^ and TfO^–^ were present, host **1** displayed cooperative binding behavior, whereby tetrafluoroborate enhanced the ability of triflate to bind (Figure 3). In the ^19^F NMR spectra (Figures 3c and S60) of the triflate salt of **1**^**H**^, the signal of triflate bound by **1**^**H**^ exhibited very low intensity. Upon progressive addition of TBABF_4_, the peaks corresponding to encapsulated TfO^–^ were observed to increase along with the increasing signal of encapsulated BF_4_^–^. We infer that after the binding of fewer than four tetrafluoroborates, the remaining empty cavities of **1**^**H**^ expanded slightly in order to adapt to the larger volume of TfO^–^. This change in the cavity size followed by enhanced binding of TfO^–^ triggered by BF_4_^–^ is a manifestation of the allosteric effect.

The ^19^F NMR peaks of encapsulated TfO^–^ were of lower intensity compared with those of BF_4_^–^. Following encapsulation, the signals of TfO^–^ shifted upfield, in the opposite direction to those of BF_4_^–^. We therefore inferred that the SO_3_ group of encapsulated TfO^–^ occupied the central space of the tetrahedral cavity, precluding multiple simultaneous TfO^–^ bindings in a way that did not block the binding of BF_4_^–^ within the peripheral spaces. Further addition of TBABF_4_ beyond 6.0 equiv appeared to disfavor the binding of TfO^–^, leading ultimately to only BF_4_^–^ binding in the cavities of **1**^**H**^, revealing a competitive binding mode in the end. After adding 14.0 equiv of TBABF_4_, the ^1^H NMR spectrum became identical with that of **1**^**H**^·BF_4_. Titration of **1**^**F**^·OTf with BF_4_^–^ afforded similar results (Figure S63). The signal of the fluorine substituent on the pyridine ring split into multiple sets of peaks, providing further evidence for the coexistence of multiple species with different numbers of bound OTf^–^ and BF_4_^–^ in slow exchange during the titration, but preventing the calculation of binding constants for this system. The appearance of initial allosteric cooperative binding behavior and then competitive binding implied some flexibility within the tightly knit framework of **1** (Figure 3d and Section 10 in the Supporting Information). The flexibility of the structure is also confirmed by the single crystal structure of **1**^**F**^·OTf. Although binding between **1** and TfO^–^ in solution is not strong, the crystal structure shows that four triflate anions are encapsulated in the solid state, similar to the structure of **1**^**F**^·BF_4_. A comparison of the cavity sizes of these two structures indicates that the cavity volume increases from 234 Å^3^ for **1**^**F**^·BF_4_ to 297 Å^3^ for **1**^**F**^·OTf (Section 10 in the Supporting Information). Furthermore, the coordination geometry of the Cu^I^ corners in **1**^**F**^·OTf shows greater distortion from ideal tetrahedral coordination compared to **1**^**F**^·BF_4_, offering more space to adapt to the larger triflate anions. Adding excess salt will increase the ionic strength and dialectric constant, which could, in turn, promote triflate encapsulation. To control for this effect, sodium tetrakis[3,5-bis(trifluoromethyl)phenyl]borate (NaBAr^F^) was added to a solution of **1**^**H**^·OTf. Upon the addition of 4.0 or 8.0 equiv of BAr^F–^, the ^19^F NMR peaks of encapsulated TfO^–^ remained unchanged (Figure S64), which indicated that the increase in the salt concentration was not responsible for triflate inclusion.

**Figure 4:**
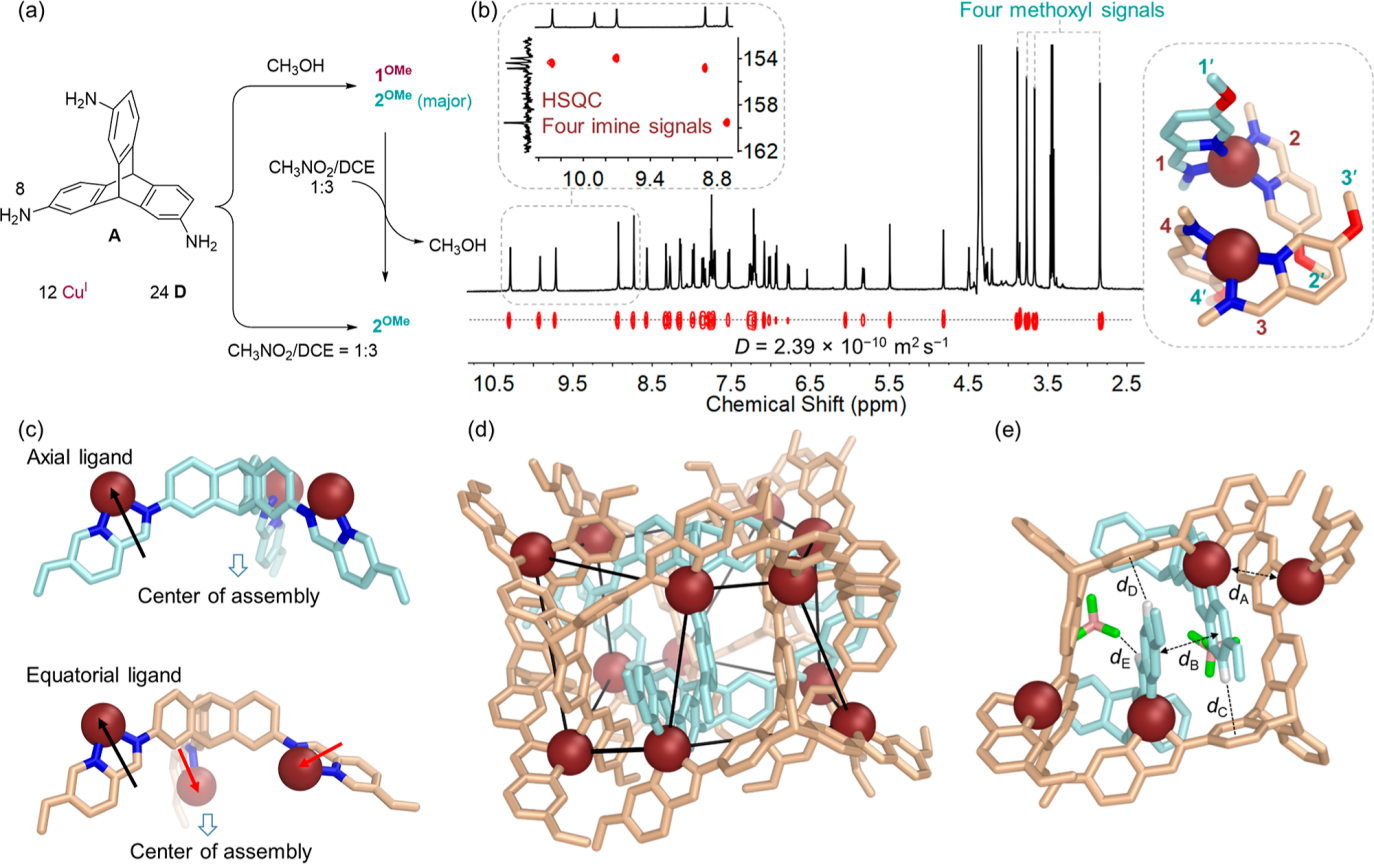
(a) Self-assembly in methanol produced a mixture of **1**^**OMe**^ and **2**^**OMe**^, whereas a 1:3 nitromethane/dichloroethane solvent resulted in the exclusive formation of **2**^**OMe**^. (b) Partial ^1^H NMR and DOSY spectra (400 MHz, 298 K, CD_3_NO_2_) of **2**^**OMe**^, with four sets of imines and methoxy groups labeled. Left inset: the imine region of the HSQC spectrum. Right inset: two adjacent Cu^I^ vertices from the X-ray crystal structure illustrate the four magnetically distinct imines (1–4, magenta) and methoxy groups (1′–4′, cyan), nitrogen atoms, blue; oxygen atoms, red. (c) Two distinct types of ligands observed within the X-ray crystal structure of **2**^**OMe**^, with inward-facing coordination vectors in red, and outward-facing vectors in black. (d) X-ray crystal structure of **2**^**OMe**^, with Cu^I^ in magenta; the six peripheral ligands are cyan, and the two central ligands are tan. Cu^I^ centers are selectively connected to illustrate the distorted hexagonal prismatic framework. Hydrogen atoms, anions, solvents, and disorder are omitted for clarity. (e) Partial view of the X-ray crystal structure of **2**^**OMe**^ down a *C*_2_ axis, where *d*_A_ shows the 5.39 Å distance between two Cu^I^ centers, *d*_B_ gives the 3.57 Å spacing between the centroids of nearest-neighbor aromatic rings, *d*_C_ and *d*_D_ show the 2.71 Å C–H···π interactions inferred to stabilize **2**^**OMe**^, and *d*_E_, one of the C–H···F interactions involved in anion binding. Hydrogen atoms are white; fluorine and boron in BF_4_^–^ are green and pink, respectively.

The reaction of **A** (8 equiv), 5-methoxy-2-formylpyridine (**D**, 24 equiv), and Cu^I^(CH_3_CN)_4_BF_4_ (12 equiv) in nitromethane/1,2-dichloroethane (DCE) (1:3, *v*/*v*) at 343 K over 48 h produced the product **2**^**OMe**^ (Figure 4a). The same reaction carried out in methanol afforded a mixture of **1**^**OMe**^ and **2**^**OMe**^, which converted into pure **2**^**OMe**^ following solvent exchange and heating. Both direct preparation and structural transformation thus resulted in the production of **2**^**OMe**^ (see Sections 5 and 11 in Supporting Information for details). ESI-MS (Figure S50) in methanol indicated a Cu^I^_12_L_8_ formulation for **2**^**OMe**^. The ^1^H NMR spectrum of **2**^**OMe**^ recorded in nitromethane-*d*_3_ was more complex than that of **1** (Figure 4b), indicating lower symmetry. The ^1^H peaks were assigned using different 2D NMR techniques, which revealed four distinct imine signals and four resonances assigned to the methoxy groups on pyridines in a 1:1:1:1 integrated ratio. All peaks exhibited the same ^1^H DOSY diffusion coefficient, indicating that they belonged to a single species (Figure 4b). Encapsulated BF_4_^–^ and free BF_4_^–^ were both also found in the ^19^F NMR spectrum of **2**^**OMe**^, revealing slow-exchange anion binding. Redissolving **2**^**OMe**^ in methanol or nitromethane did not cause decomposition, consistent with the higher stability of **2** (Figure S51) relative to **1**.

Vapor diffusion of diisopropyl ether into a methanol solution of **2**^**OMe**^ produced single crystals that were suitable for single-crystal X-ray diffraction with synchrotron radiation. Although the single-crystal structure revealed the same Cu_12_L_8_ composition as that of **1**, the ligand and metal arrangements are distinct from those of **1** (Figure 4d). Twelve Cu^I^ centers define a distorted hexagonal prismatic array, with four ligand environments and two Cu^I^ environments, lending the assembly *D*_3_ point-group symmetry.

In the structure of **2**^**OMe**^, two of the eight ligands (Figure 4, light cyan) are axial, defining the top and bottom of the prism and the *C*_3_ axis of the assembly. This principal symmetry axis, together with the three *C*_2_ axes that pass between the closest spaced pairs of pyridines, thus generate the *D*_3_ symmetry of the assembly. The coordination vectors of these two ligands point outward from the center of the assembly. The other six equatorial ligands (Figure 4, tan) are symmetry-equivalent and define the walls of the prism. Within each of these six, the coordination vector of one iminopyridine points out from the center of the assembly, and the other two point inward (Figure 4c).

Two distinct Cu^I^ environments are observed in **2**^**OMe**^. Half of the 12 Cu^I^ centers are exclusively coordinated by equatorial ligands, while the other six Cu^I^ centers are coordinated by both types of ligands. This arrangement gives rise to four distinct ligand-arm environments that generate the four sets of peaks observed by ^1^H NMR. The inset at the right in Figure 4b displays a pair of distinct Cu^I^ centers and their ligand environments, illustrating the four magnetically distinct imine protons and methoxy groups (marked with 1–4 and 1′–4′, respectively). Each such pair of Cu^I^ centers is separated by 5.39 ± 0.24 Å (distance *d*_A_ in Figure 4e).

Different stabilizing supramolecular interactions within **2**^**OMe**^ are shown in Figure 4e. The centroid-to-centroid distances (Figure 4e, *d*_B_) between face-to-face pyridine rings are 3.57 ± 0.03 Å, consistent with effective arene stacking. We infer that such stacking, favored by the electron-donating methoxy substituent, provides a driving force for the formation of **2**^**OMe**^ incorporating subcomponent **D**. Incorporation of the electron-withdrawing fluorine substituent on subcomponent **B** renders stacking less favorable, destabilizing a structure analogous to that of **2**. Multiple C–H···π interactions (Figure 4e, *d*_C_ and *d*_D_) between pyridyl and triptycene are also observed, with an average distance of 2.71 ± 0.06 Å and an angle of 159.7° ± 6.5°. Both stacking and C–H···π interactions are thus inferred to contribute to the formation of compact and highly-ordered **2**.

The cavity of **2**^**OMe**^ is occupied by three BF_4_^–^ anions in the crystal, consistent with the slow exchange of BF_4_^–^ observed in the ^19^F NMR spectrum. C–H···F hydrogen bonds (Figure 4e, *d*_E_) are observed between BF_4_^–^ and pyridine hydrogen atoms; we infer these interactions are also strengthened by electrostatic attraction.^22^ This anion is nonetheless not required for formation of **2**. The same framework of **2** was also formed when Cu^I^(CH_3_CN)_4_OTf was used in place of the tetrafluoroborate, as observed by ESI-MS and NMR spectroscopy (see Section 8 in Supporting Information). Notably, in contrast to **1**, which preferentially bound BF_4_^–^, its ^19^F NMR spectrum revealed that **2** readily accommodated OTf^–^, with slow-exchange binding on the NMR time scale. Integration of its ^19^F NMR spectrum suggested that only one OTf^–^ was bound within the cavity of **2**. Furthermore, titration of **2**^**OMe**^·OTf with TBABF_4_ revealed that the anion-binding behavior of **2** is different from that of **1** (Figure S65). The peak corresponding to encapsulated TfO^–^ was observed to decrease during the progressive addition of BF_4_^–^. After the addition of 10.0 equiv of BF_4_^–^, the ^1^H NMR spectrum became messy and precipitates formed, allowing us to conclude that **2**^**OMe**^·BF_4_ did not form.

**Figure 5:**
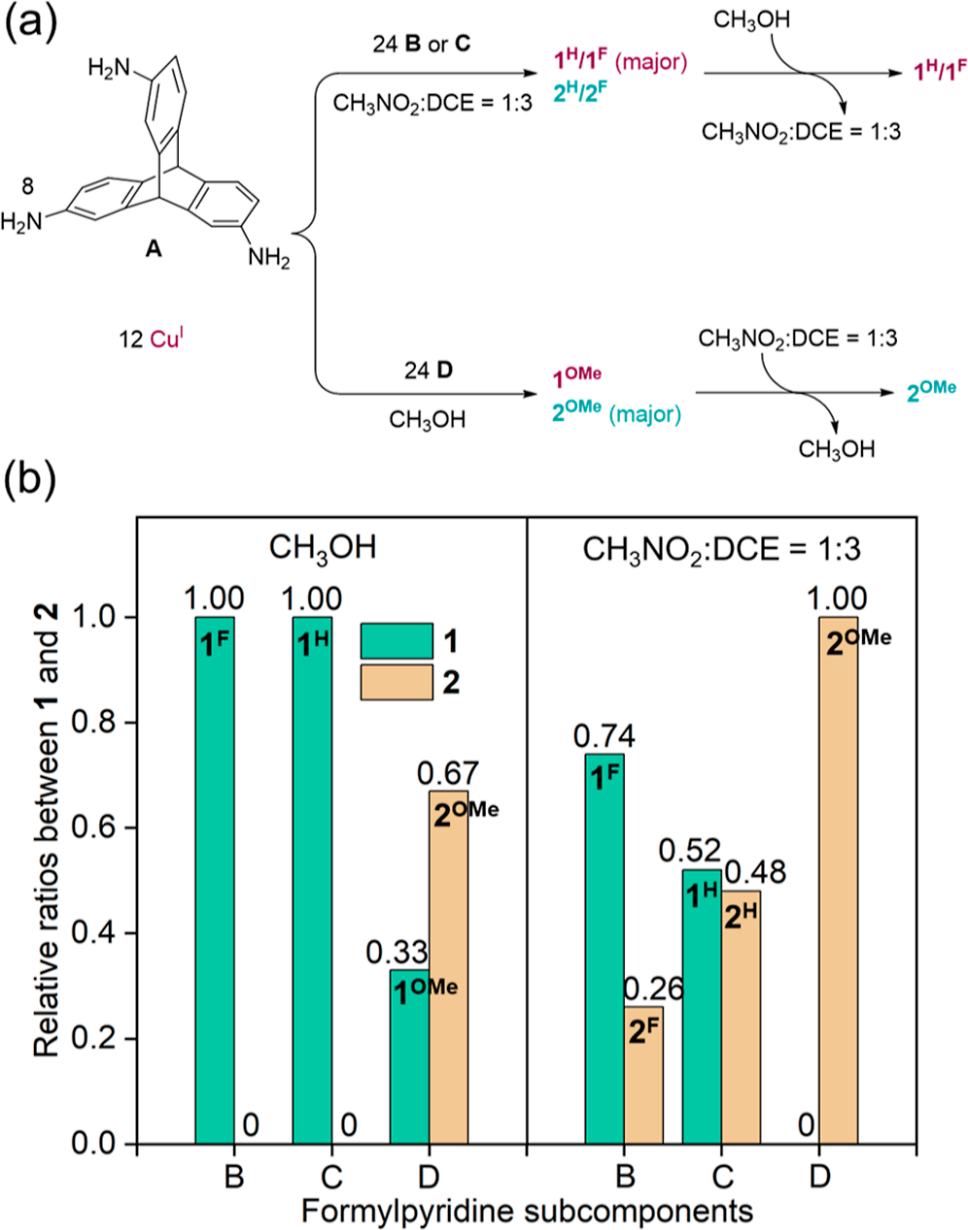
(a) Syntheses and solvent-driven transformations between the frameworks of **1** and **2** under different conditions. (b) Relative ratios of **1** and **2** determined by integration of ^1^H NMR spectra of the assemblies using different formylpyridine subcomponents and solvent systems.

As discussed above, the preparation of pure frameworks **1** and **2** required specific subcomponents and solvent systems. Reactions employing aldehydes **B** or **C** in methanol gave pure **1**, whereas changing the aldehyde to **D** and the solvent from methanol to nitromethane/dichloroethane led to the formation of pure **2**. We thus explored which factor played a more important role in determining the reaction product. Six independent syntheses were carried out using three formylpyridine derivatives and two solvent systems (Figure 5a). The ratio between **1** and **2** formed was in each case determined by the integration of ^1^H NMR spectra (Figure S66). As shown in Figure 5b, in methanol, the incorporation of electron-poor **B** or **C** afforded pure **1**^**F**^ or **1**^**H**^, whereas **2**^**OMe**^ became dominant when the more electron-rich **D** was used, indicating that the electronics of the formylpyridine subcomponent predominated over solvent effects in determining the product structure.^23^

Reactions undertaken in 1:3 nitromethane/DCE exhibited the same substituent dependence. The **1**-to-**2** ratio decreased from 0.74 to 0.52 when the electron-withdrawing fluorine of **B** was replaced with the hydrogen of **C**, and framework **1** disappeared altogether when the electron-rich methoxy groups of the **D** residues were incorporated. The electron density on the subcomponent thus determined which assembly predominated, and the selectivity could be further optimized using solvent effects.

A mixture of **1** and **2** was observed to transform into a pure assembly upon solvent exchange. In methanol, the self-assembly reaction employing methoxyformylpyridine **D** afforded a mixture of **1**^**OMe**^ and **2**^**OMe**^, with **2**^**OMe**^ as the major product. Subsequent evaporation of methanol and dissolution in 1:3 nitromethane/DCE led to the formation of pure **2**^**OMe**^ as the equilibrium shifted away from **1**^**OMe**^ to **2**^**OMe**^ (Figure 5a). Likewise, a mixture of **1**^**F**^ and **2**^**F**^ transformed into pure **1**^**F**^ upon a change of solvent from nitromethane/DCE to methanol, demonstrating a stimulus-responsive structural transformation.^24^

Moreover, both compounds **1** and **2** are emissive in methanol. Photoluminescence studies suggested that solutions of **1** and **2** exhibited broad emissive bands ranging from 450 to 550 nm, with a fine structure observed (Figure S67). We infer that the compact nature of the assemblies minimizes nonradiative decay and boosts photoluminescence.

## Conclusions

The sterics and geometrical arrangement of the three amino groups of triptycene-based subcomponent **A** thus precluded the formation of structures with simpler helicate or Platonic-solid geometries, instead leading to the more complex Cu^I^_12_L_8_ frameworks of **1** and **2**, with substituent and solvent effects allowing one or the other to be prepared exclusively. The present use of geometrical and steric frustration may allow larger and more complex architectures to form by using flexible Cu^I^ as a structural metal ion. The ability of **1** to display complex multiple-anion-binding behavior suggests potential uses for these architectures in guest-binding systems. Moreover, in contrast to other examples of allosteric binding behavior in cages where the cavity size is altered in response to binding events occurring peripherally,^25^ the initial guest binding in the cavity of **1** promotes the encapsulation of another larger guest within the same cavity. Such multiguest responsive behavior may enable the triggered uptake or release of one guest upon treatment with another in the context of chemical purifications. Larger such systems may prove useful in the selective uptake or sensing of biological substrates in water, given the utility of copper(I) complexes in this solvent.^26^

## Supporting Information Available

The Supporting Information is available free of charge at https://pubs.acs.org/doi/10.1021/jacs.3c09547. Crystallographic data for the structures reported in this paper have been deposited at the Cambridge Crystallographic Data Centre under the deposition numbers 2224726 (**1**^**F**^·BF_4_), 2300612 (**1**^**F**^·OTf), and 2233061 (**2**^**OMe**^·BF_4_). Copies of these data can be obtained free of charge via www.ccdc.cam.ac.uk/data_request/cif.

Experimental procedures, NMR spectra, single crystal analysis, photophysical results, and computational details (PDF)
