# Supplementary material for: Steric and Geometrical Frustration Generate Two Higher-Order CuI12L8 Assemblies from a Triaminotriptycene Subcomponent
Source: J Am Chem Soc. 2024 Jan 22;146(4):2370–8. doi: 10.1021/jacs.3c09547 (PMC10835662; doi:10.1021/jacs.3c09547)
Supplement: Supplementary file 1 — ja3c09547_si_001.pdf [file ja3c09547_si_001.pdf]

Steric and geometrical frustration generate two higher-order $Cu^{I}_{12}L_8$ assemblies from a triaminotriptycene subcomponent

Huangtianzhi Zhu, Tanya K. Ronson, Kai Wu and Jonathan R. Nitschke*

Department of Chemistry, University of Cambridge, Lensfield Road, Cambridge CB2 1EW, U.K

Table of Contents

1. Materials and methods……………………………………………………………………………S2

2. Synthesis of subcomponents…………...…………………………………………………………S3

3. Synthesis and characterization of **1**$^{\mathbf{H}}$•BF_4……………………………………………………S5

4. Synthesis and characterization of **1**$^{\mathbf{H}}$•OTf……………...……………………………………S11

5. Synthesis and characterization of **1**$^{\mathbf{F}}$•BF_4……………………………………………………S17

6. Synthesis and characterization of **1**$^{\mathbf{F}}$•OTf ……….………..…………………………………S24

7. Synthesis and characterization of **2**$^{\mathbf{OMe}}$•BF_4…………………………………………………S30

8. Synthesis and characterization of **2**$^{\mathbf{OMe}}$•OTf …….……………………..…………………S38

9. Solvent effect on the self-assembly……….……………………………………………………S44

10. BF_4^- binding of **1**$^{\mathbf{H/F}}$•OTf and **2**$^{\mathbf{OMe}}$•OTf ……………………………………………………S45

11. Effects of solvents and electron density of subcomponents………………………………………S49

12. Photoluminescence studies of **1** and **2**………………………………………...……...…S51

13. X-ray crystallography……………...……………………………………………………….S52

1 4 .
References……………………...……………………………………….………………......S586

1. Materials and methods

Unless otherwise specified, all starting materials were purchased from commercial sources and used as supplied. 2,7,14-trinitrotriptycene was prepared according to a published procedure.[1] 5-Hydroxy-2-formylpyridine was purchased from Fluorochem. Solvents were used as supplied.

NMR spectra were recorded using 400 MHz Avance III HD Smart Probe (routine ^{1}H NMR, DOSY) and DCH 500 MHz dual cryoprobe (high-resolution ^{13}C and 2D experiments) NMR spectrometers. Chemical shifts (δ) for ^{1}H NMR spectra are reported in parts per million (ppm) and are reported relative to the solvent residual peak. Coupling constants (J) were reported in Hz to 1 decimal place. The 1H NMR spectra recorded in a mixture of nitromethane-d_3 and non-deuterated 1,2-dichloroethane (1:3, *v*/*v*) were shown as acquired, without signal suppression. ^{1}H DOSY NMR experiments were conducted on a Bruker 400 MHz Avance III HD Smart Probe spectrometer. Maximum gradient strength was 5.35 G/cm A. The standard Bruker pulse program, ledbpgp2s,3 employing a stimulated echo and longitudinal eddy-current delay (LED) using bipolar gradient pulses for diffusion using 2 spoil gradients, was utilized. A gradient ramp of 5% to 90% was used. Low-resolution electrospray ionisation mass spectra were recorded on a Micromass Quattro LC instrument. High-resolution electrospray ionisation mass spectra were recorded on a Waters Synapt G2-Si instrument.

2. Synthesis of subcomponents

2.1 Synthesis of 2,7,14-triaminotriptycene

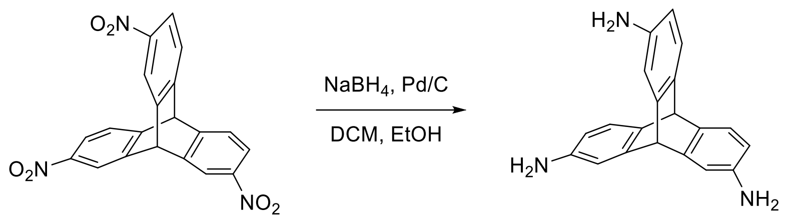

Scheme S1. Synthesis of 2,7,14-triaminotriptycene

The procedure was modified from a published one.[1] To a round-bottom flask, 2,7,14-trinitrotriptycene (389 mg, 1.00 mmol), palladium on carbon (Pd/C, 38.9 mg), sodium borohydride (378 mg, 10 mmol) and dichloromethane (15.0 mL) were added. Ethanol (15.0 mL) was added dropwise over 2 h, and the mixture was stirred overnight under protection of nitrogen. After filtration, the solution was concentrated. The resulting solid was purified by column chromatography using dichloromethane : methanol = 20 :1 as the eluent to afford 2,7,14-triaminotriptycene as a pale yellow powder (190 mg, yield 63.3%). ^{1}H NMR (400 MHz, 298 K, CD_3CN) δ 6.99 (d, J = 7.7 Hz, 3H), 6.72 (d, J = 2.3 Hz, 3H), 6.23 (dd, J = 7.7, 2.3 Hz, 3H), 5.04 (s, 1H), 5.02 (s, 1H), 3.97 (s, 6H).

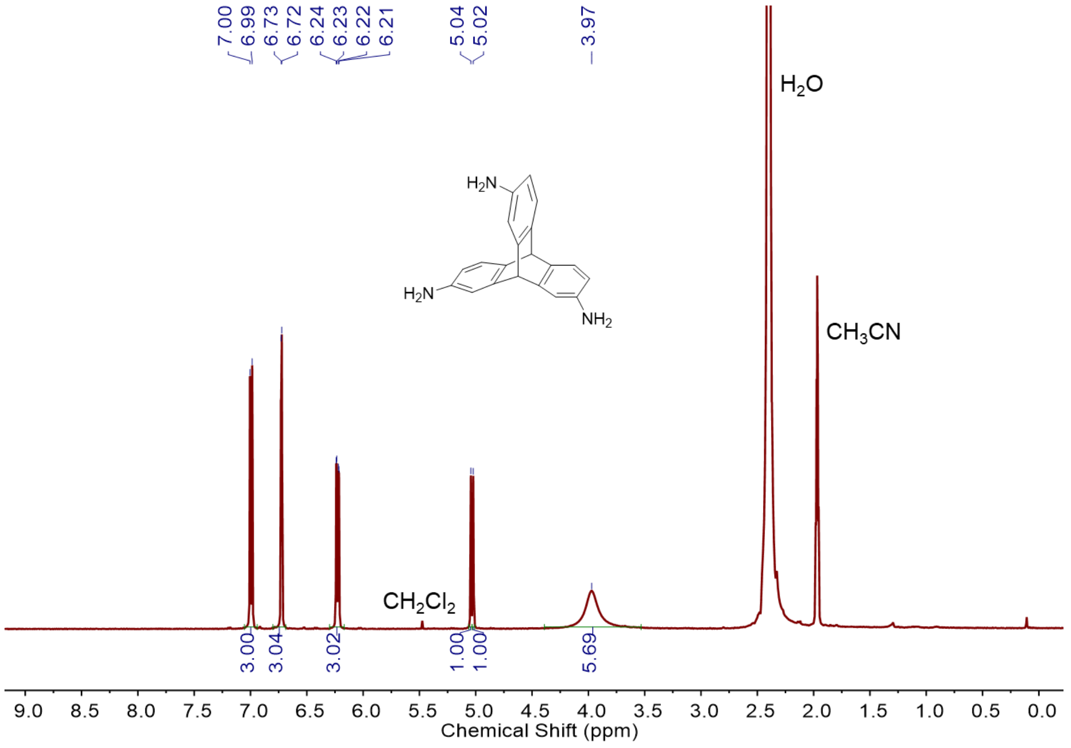

Figure S1. ^{1}H NMR spectrum (400 MHz, 298 K, CD_3CN) of 2,7,14-triaminotriptycene.

2.2 Synthesis of 5-methoxy-2-formylpyridine

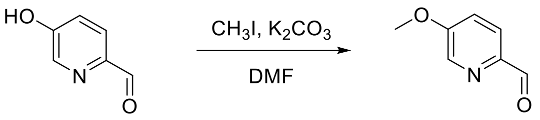

Scheme S2. Synthesis of 5-methoxy-2-formylpyridine

To a round bottom flask, 5-hydroxy-2-formylpyridine (300 mg, 2.44 mmol), K_2CO_3 (1.38 g, 10.0 mmol) and *N*,*N*-dimethylformamide (30.0 mL) were added under protection of nitrogen. After heating to 90 °C, iodomethane (450 mg, 3.17 mmol) was added, and the mixture was stirred for 2 h. After completion, the solvent was removed in vacuum, and the crude product was purified by column chromatography using hexane : ethyl acetate = 2:1 as the eluent to afford 5-methoxy-2-formylpyridine as a pale yellow crystal (242 mg, yield 72.5%). ^{1}H NMR (400 MHz, 298 K, CD_3CN) δ 9.94 (s, 1H), 8.47 (d, *J* = 2.8 Hz, 1H), 7.96 (d, *J* = 8.7 Hz, 1H), 7.47 (dd, *J* = 8.7, 2.8 Hz, 1H), 3.97 (s, 3H).

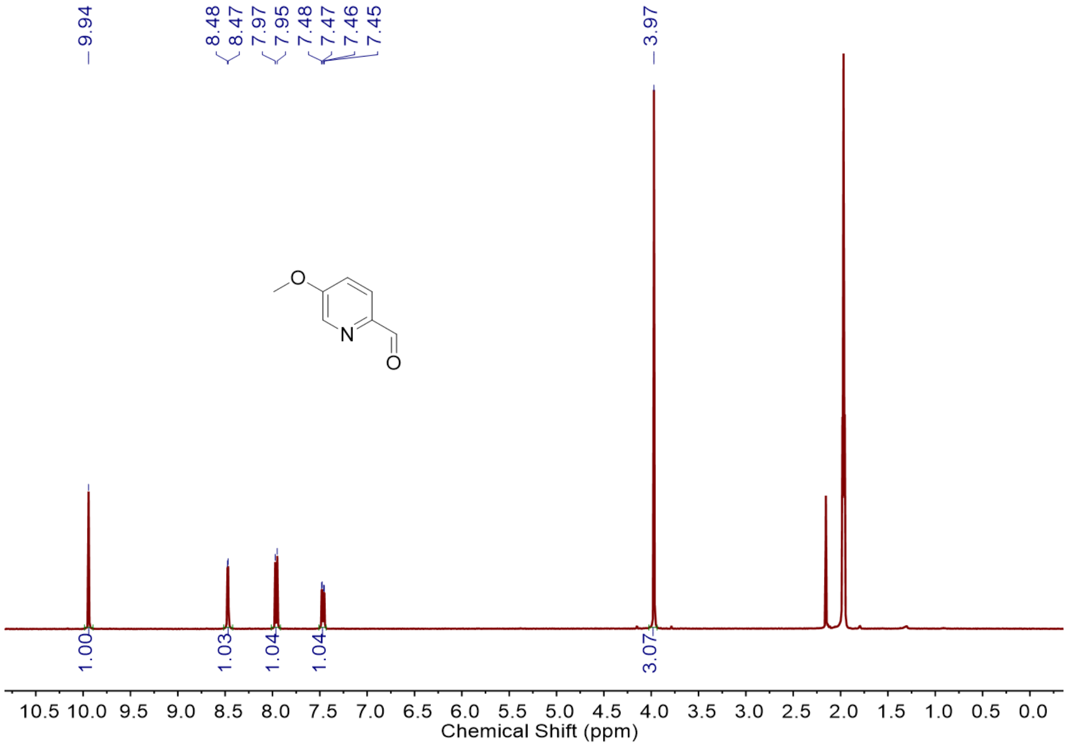

Figure S2. ^{1}H NMR spectrum (400 MHz, 298 K, CD_3CN) of 5-methoxy-2-formylpyridine.

3. Synthesis and characterization of $\mathbf{1^H}$•BF_4

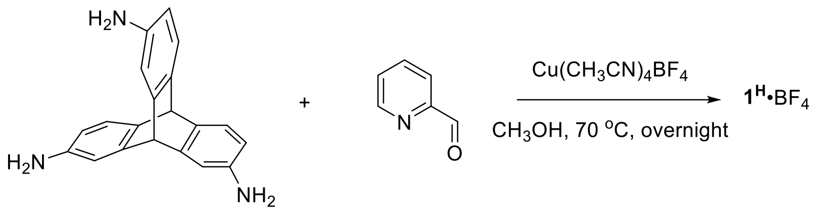

Scheme S3. Synthesis of $\mathbf{1^H}$•BF_4.

2,7,14-triaminotriptycene (1.00 mg, 2 equiv, 3.34 μmol), 2-formylpyridine (1.07 mg, 6 equiv, 10.0 μmol), tetrakis(acetonitrile)copper(I) tetrafluoroborate (1.58 mg, 3 equiv, 5.01 μmol) and 0.70 mL of methanol (using less solvent, 0.50 mL, yielded a heterogeneous suspension but the assembly was identical) were added into a small vial that was sealed in glove box. The vial was kept at 343 K overnight, affording a red solution. The solvent was reduced by nitrogen flow, followed by the addition of diethyl ether. Precipitates were collected by centrifugation and washed with ether three times. After drying in vacuum, $\mathbf{1^H}$•BF_4 was obtained as a deep red solid in approximately quantitative yield. ^{1}H NMR (400 MHz, 298 K, CD_3OD) δ 10.01 (s, 12H, one imine), 9.88 (s, 12H), 9.09 – 8.96 (m, 24H, containing the other imine), 8.29 (d, J = 5.1 Hz, 12H), 8.17 (t, J = 7.8 Hz, 12H), 8.10 (d, J = 7.9 Hz, 12H), 7.93 (s, 4H, alkyl bridge), 7.76 – 7.69 (m, 12H), 7.23 (d, J = 8.0 Hz, 12H), 7.12 (d, J = 7.9 Hz, 12H), 7.02 (d, J = 7.9 Hz, 12H), 6.98 – 6.92 (m, 12H), 6.61 (t, J = 7.7 Hz, 12H), 6.55 (d, J = 8.0 Hz, 12H), 6.37 (d, J = 2.0 Hz, 12H), 5.92 (d, J = 7.8 Hz, 12H), 5.62 (s, 4H, alkyl bridge), 5.30 (s, 4H, alkyl bridge), 4.85 (4H, alkyl bridge buried in the peak of H_2O). ^{13}C NMR (126 MHz, 298 K, CD_3OD) δ 158.8 (imine), 158.6 (the other imine), 152.9, 152.0, 150.3, 149.3, 148.0, 147.8, 147.7, 146.8, 145.7, 144.0, 139.8, 138.9, 130.3, 128.8, 127.5, 126.4, 125.5, 121.2, 117.9, 116.7, 56.0 (alkyl bridge), 54.0 (alkyl bridge), 53.8 (alkyl bridge), 52.0 (alkyl bridge). ^{19}F NMR (376 MHz, 298 K, CD_3OD) δ –142.76 (encapsulated BF_4^-), –153.50 (free BF_4^-). High-resolution ESI-MS: m/z = 818.4176 $[\mathbf{1^H} + (BF_4)_5]^{7+}$, 969.4896 $[\mathbf{1^H} + (BF_4)_6]^{6+}$, 1180.5889 $[\mathbf{1^H} + (BF_4)_7]^{5+}$, 1497.2359 $[\mathbf{1^H} + (BF_4)_8]^{4+}$.

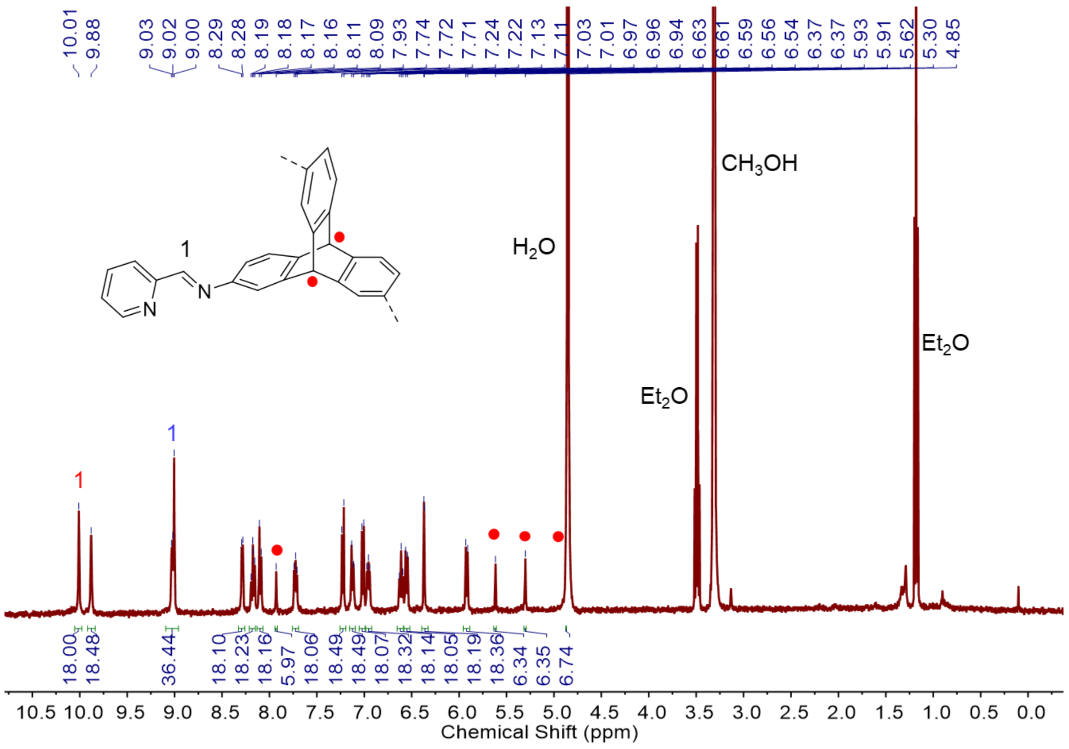

Figure S3. ^{1}H NMR spectrum (400 MHz, 298 K, CD_3OD) of $\mathbf{1^{H}}$•BF_4.

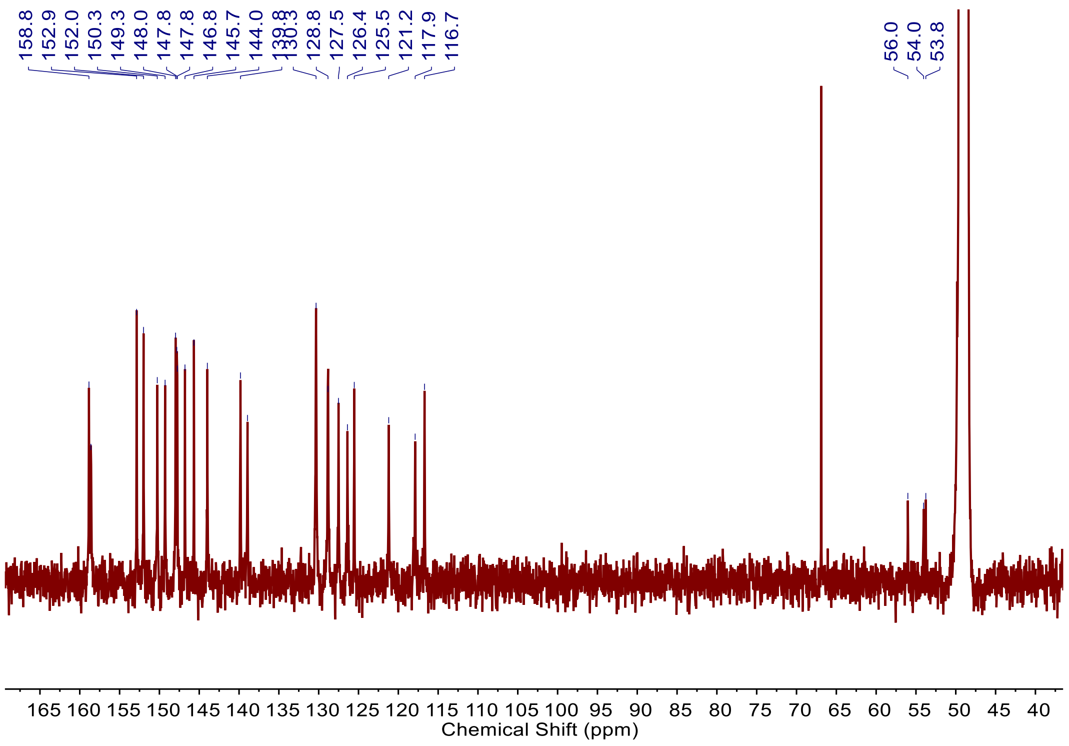

Figure S4. ^{13}C NMR spectrum (126 MHz, 298 K, CD_3OD) of $\mathbf{1^{H}}$•BF_4.

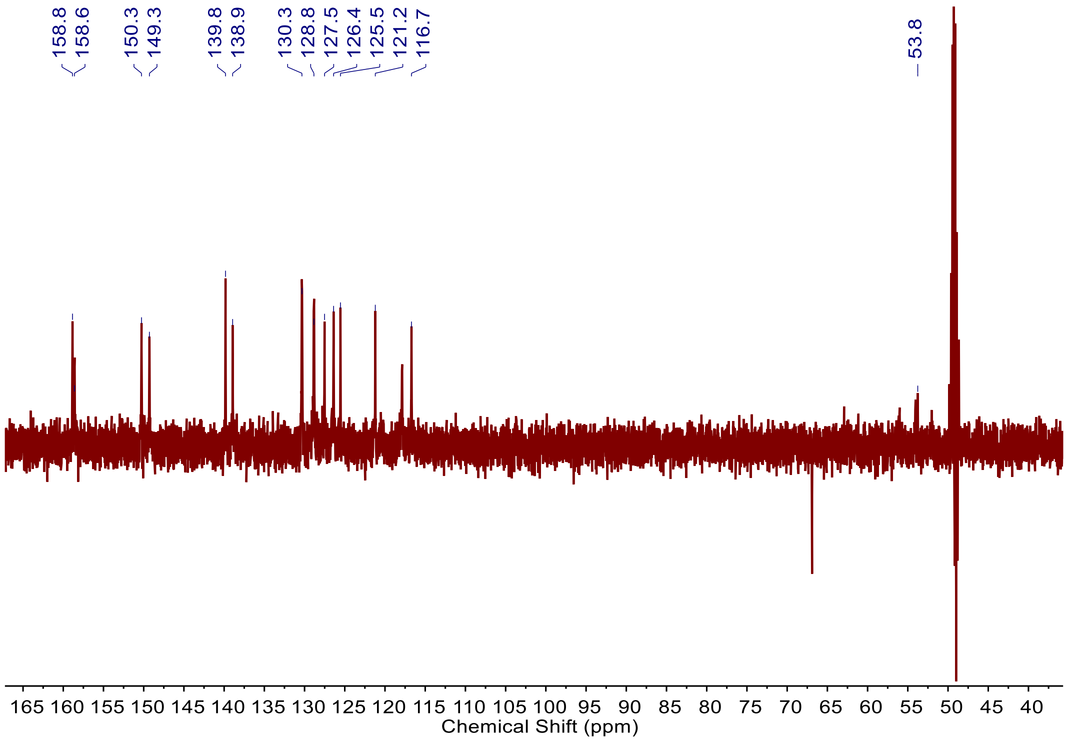

Figure S5. ^{13}C dept NMR spectrum (126 MHz, 298 K, CD_3OD) of $\mathbf{1^H}$•BF_4.

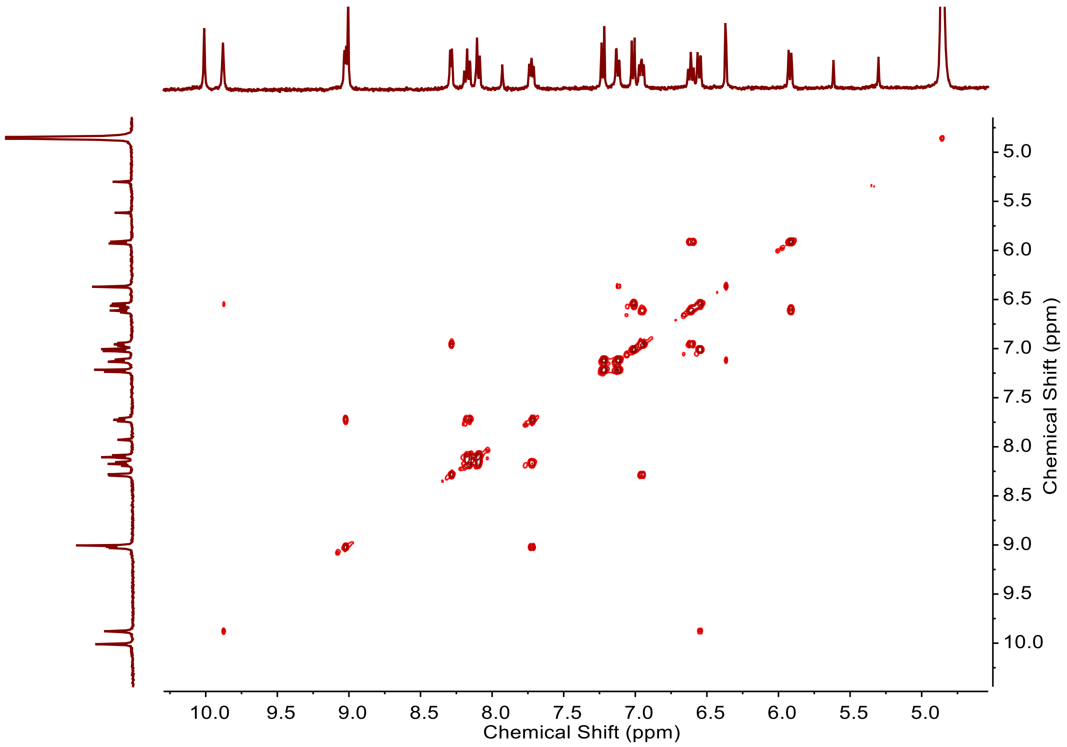

Figure S6. ^{1}H-^{1}H COSY spectrum (400 MHz, 298 K, CD_3OD) of $\mathbf{1^H}$•BF_4.

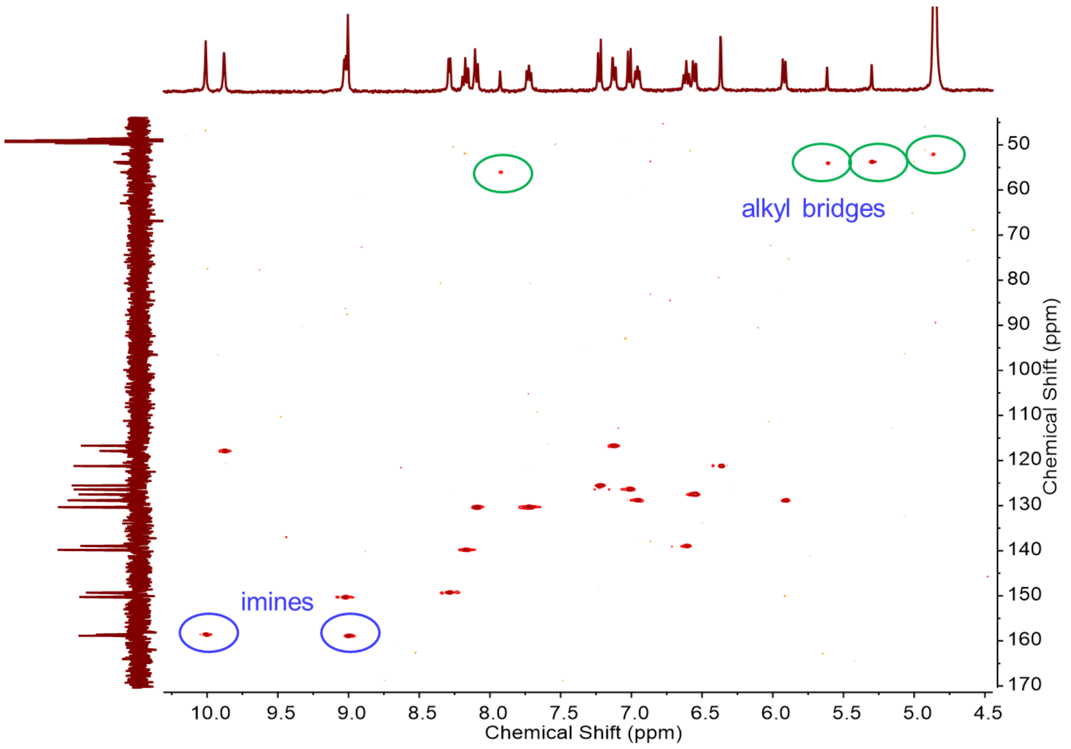

Figure S7. ^{1}H-^{13}C HSQC spectrum (400 MHz, 298 K, CD_3OD) of $\mathbf{1^{H}}$•BF_4.

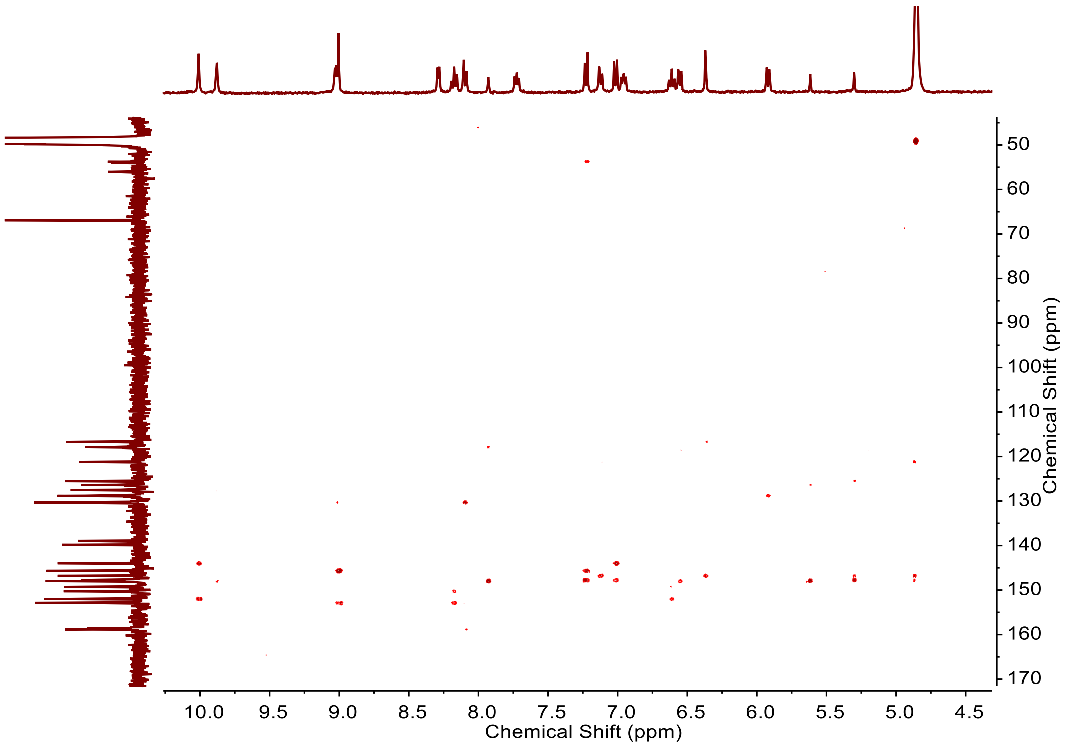

Figure S8. ^{1}H-^{13}C HMBC spectrum (400 MHz, 298 K, CD_3OD) of $\mathbf{1^{H}}$•BF_4.

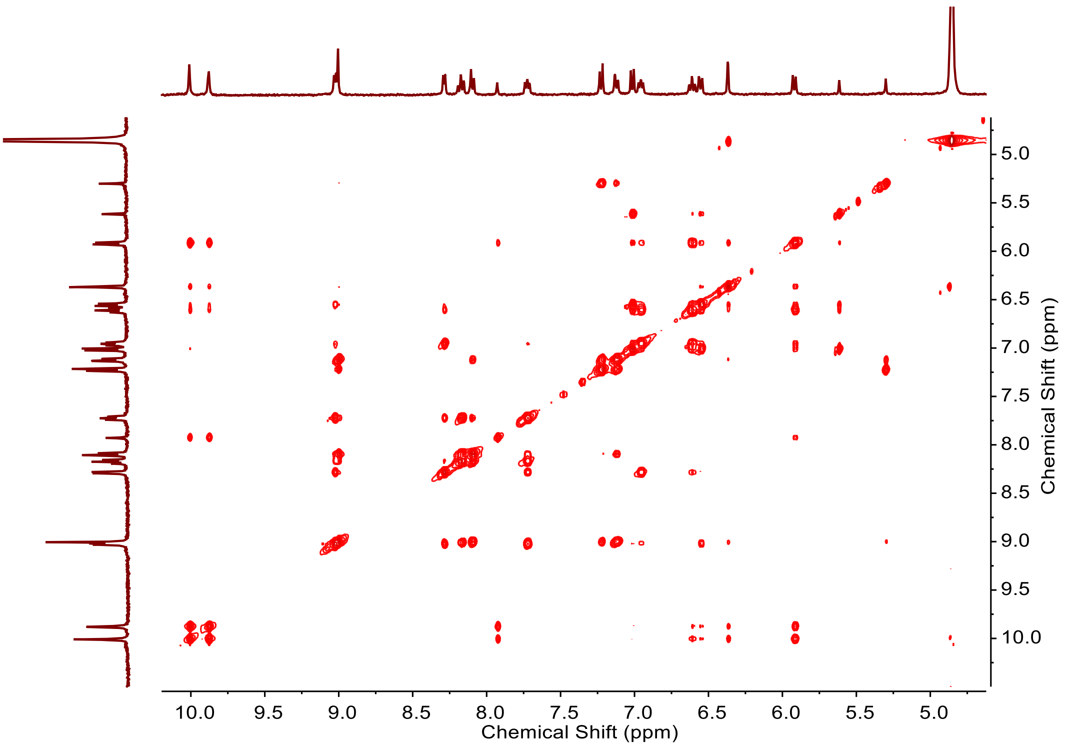

Figure S9. ^{1}H-^{1}H NOESY spectrum (400 MHz, 298 K, CD_3OD) of $\mathbf{1^{H}}$•BF_4.

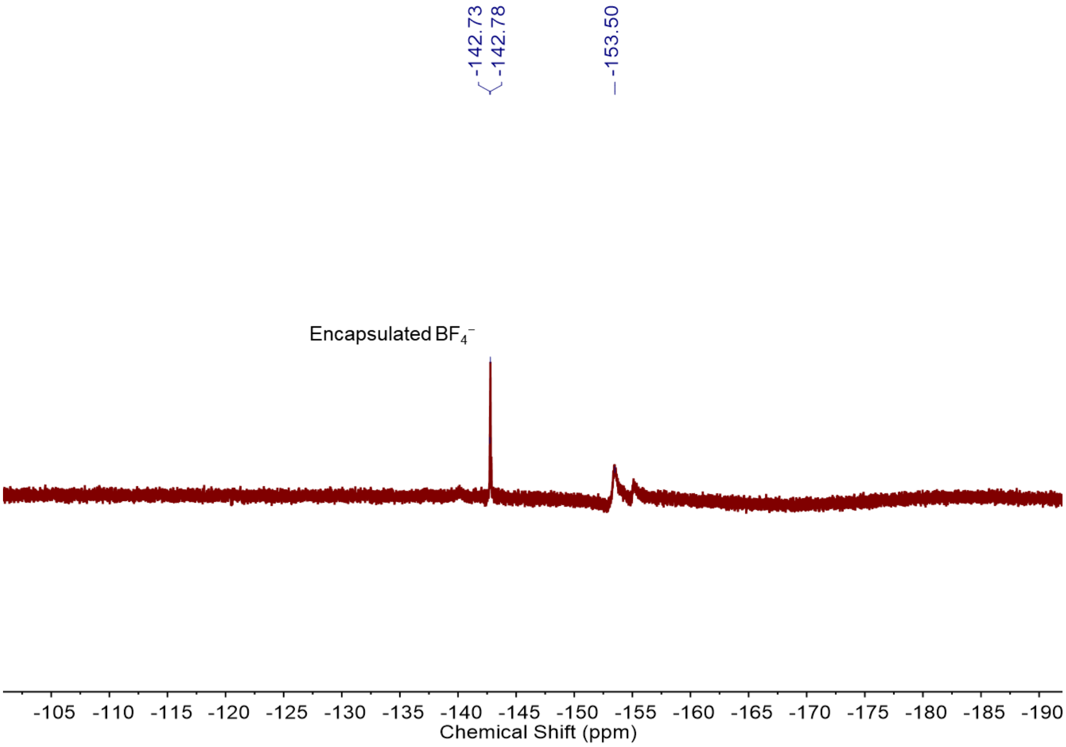

Figure S10. ^{19}F NMR spectrum (376 MHz, 298 K, CD_3OD) of $\mathbf{1^{H}}$•BF_4.

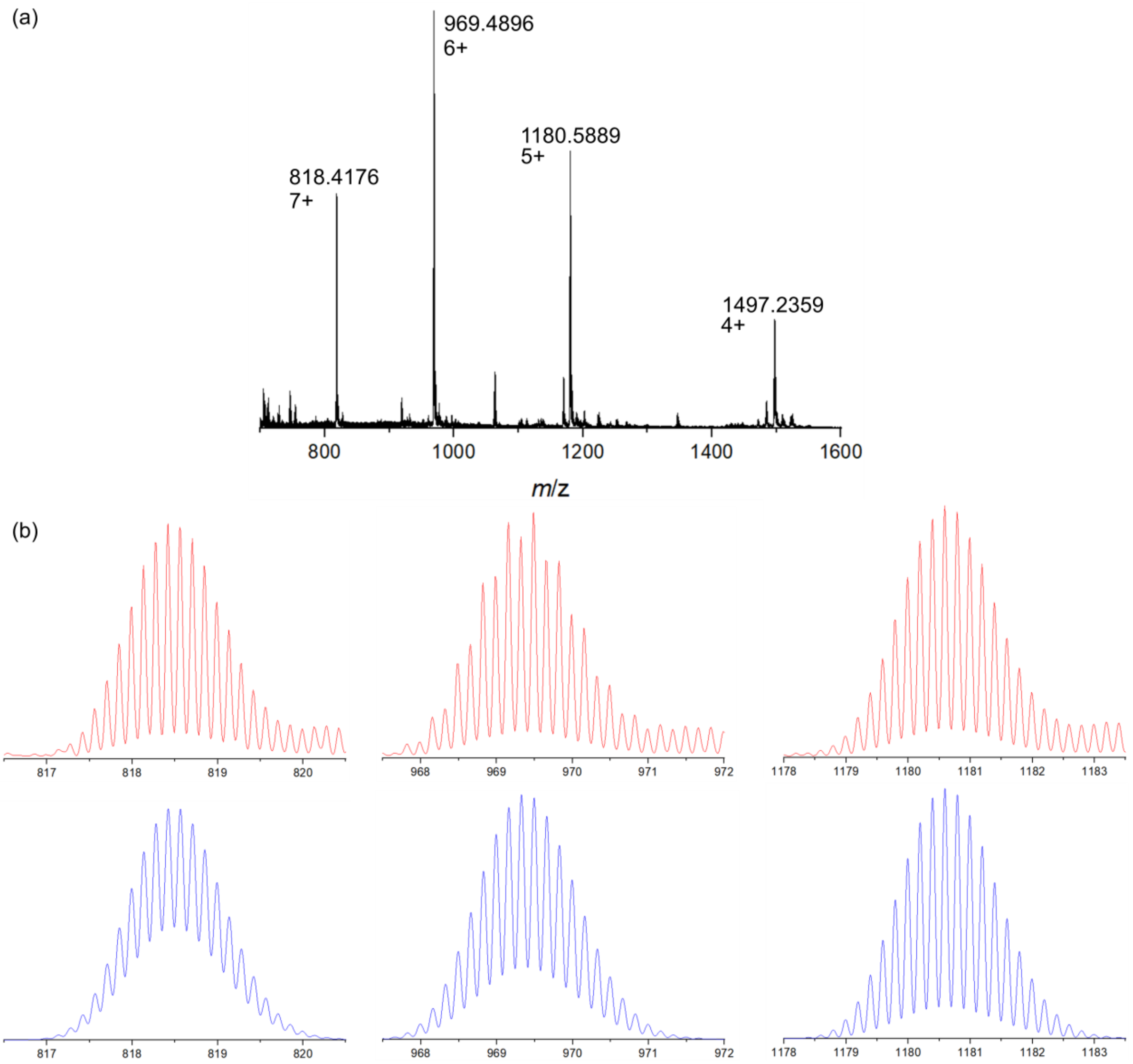

Figure S11. High-resolution ESI-mass spectrum of $\mathbf{1^H}$•BF_4: (a) Full spectrum and (b) selected cations (7+, 6+, 5+, from left to right) with experimental (red) and calculated (blue) isotopic distributions.

4. Synthesis and characterization of $\mathbf{1^{H}}$•OTf

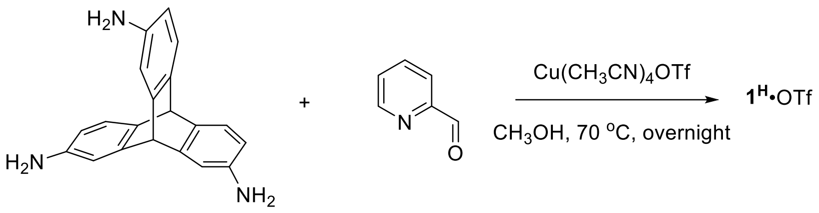

Scheme S4. Synthesis of $\mathbf{1^{H}}$•OTf.

2,7,14-triaminotriptycene (1.00 mg, 2 equiv, 3.34 μmol), 2-formylpyridine (1.07 mg, 6 equiv, 10.0 μmol), tetrakis(acetonitrile)copper(I) triflate (1.89 mg, 3 equiv, 5.01 μmol) and 0.70 mL of methanol were added into a small vial that was sealed in glove box. The vial was kept at 343 K overnight, affording a red solution. The solvent was reduced by nitrogen flow, followed by the addition of diethyl ether. Precipitates were collected by centrifugation and washed with ether three times. After drying in vacuum, $\mathbf{1^{H}}$•OTf was obtained as a deep red solid in approximately quantitative yield. ^{1}H NMR (500 MHz, 298 K, CD_3OD) δ 10.41 (s, 12H, imine), 10.28 (s, 12H), 9.07 (s, 24H, containing the other imine), 8.54 (d, J = 4.9 Hz, 12H), 8.44 (s, 4H, alkyl bridge), 8.20 (d, J = 4.8 Hz, 16H), 8.14 (dd, J = 7.7, 1.6 Hz, 12H), 8.08 (d, J = 7.9 Hz, 12H), 7.64 (t, J = 6.8 Hz, 12H), 7.31 (d, J = 8.1 Hz, 12H), 7.25 (d, J = 8.2 Hz, 12H), 7.12 (d, J = 7.9 Hz, 12H), 6.97 – 6.89 (m, 12H), 6.52 (dd, J = 7.8, 2.1 Hz, 12H), 6.46 (td, J = 7.8, 1.6 Hz, 12H), 6.34 (s, 12H), 6.20 (s, 12H), 5.76 (s, 4H, alkyl bridge), 5.41 (s, 4H, alkyl bridge). The signal of one alkyl bridge (possibly buried in the peak of H_2O) is missing in ^{1}H, ^{13}C and HSQC spectra, but the structure of this assembly should be the same as the previous one, as evidenced by two imines and mass spectrum. ^{13}C NMR (126 MHz, 298 K, CD_3OD) δ 157.4 (two imines overlap here based on HSQC), 151.4, 150.6, 148.3, 147.7, 147.5, 147.0, 145.8, 145.5, 144.0, 142.8, 138.4, 137.1, 128.9, 127.2, 125.8, 124.7, 124.5, 121.7, 120.0, 119.1, 118.6, 115.7, 53.8 (alkyl bridge), 52.9 (alkyl bridge), 52.2 (alkyl bridge). ^{19}F NMR (376 MHz, 298 K, CD_3OD) δ –74.00 (encapsulated OTf^{-}), –79.92 (free OTf^{-}). ESI-MS: m/z = 818.4176 [$\mathbf{1^{H}}$ + $(OTf)_5]^{7+}$, 969.4896 [$\mathbf{1^{H}}$ + $(OTf)_6]^{6+}$, 1180.5889 [$\mathbf{1^{H}}$ + $(OTf)_7]^{5+}$, 1497.2359 [$\mathbf{1^{H}}$ + $(OTf)_8]^{4+}$.

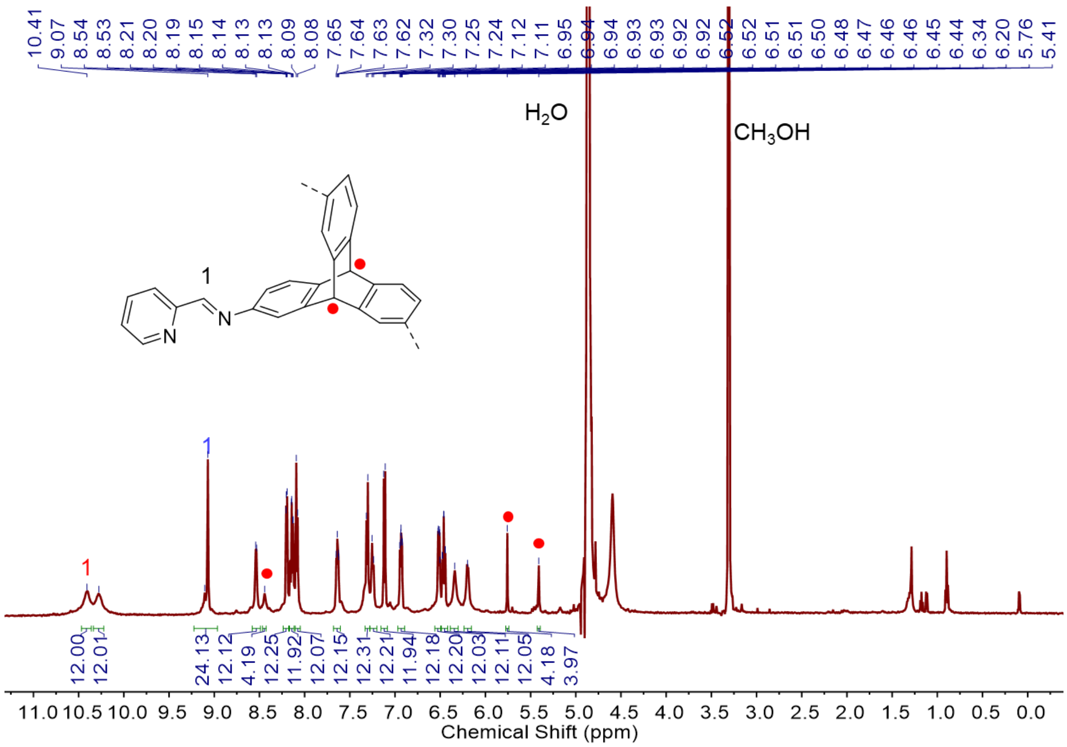

Figure S12. ^{1}H NMR spectrum (500 MHz, 298 K, CD_3OD) of **1^{H}**•OTf.

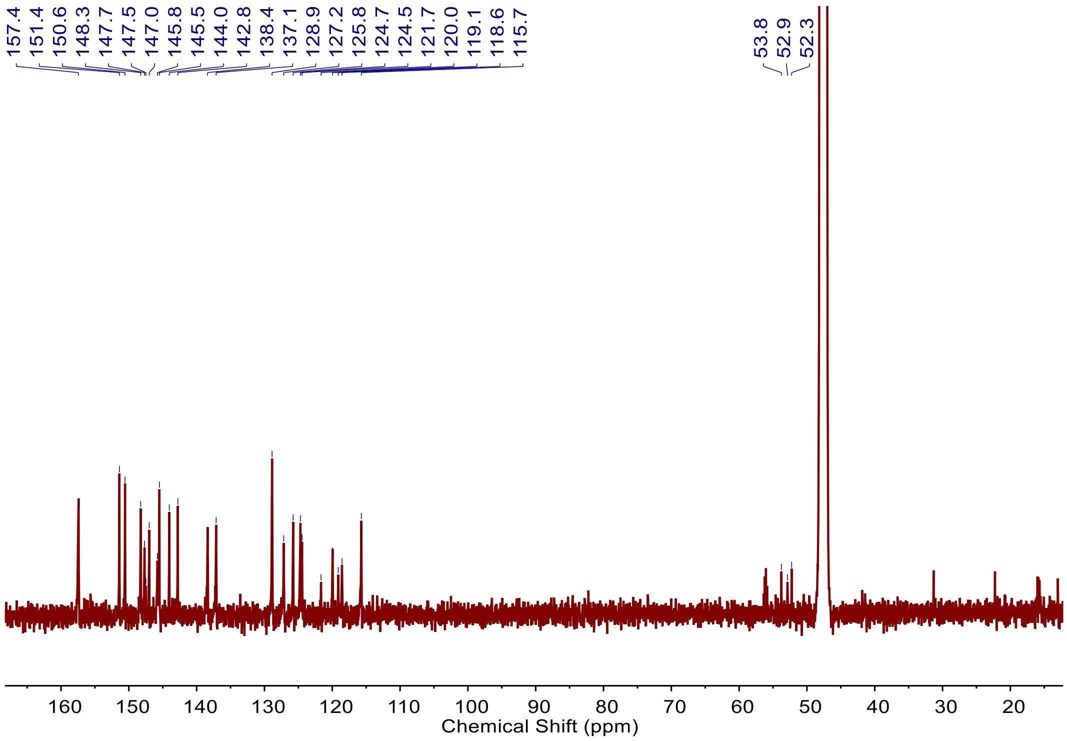

Figure S13. ^{13}C NMR spectrum (126 MHz, 298 K, CD_3OD) of **1^{H}**•OTf.

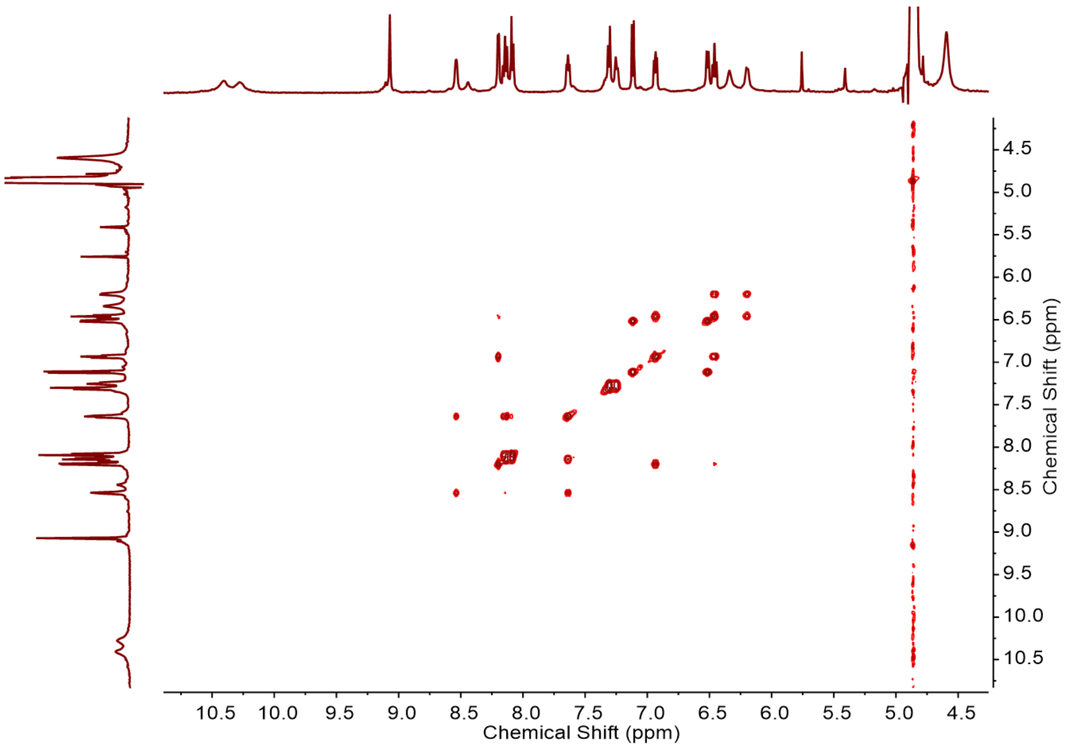

Figure S14. ^{1}H-^{1}H COSY spectrum (500 MHz, 298 K, CD_3OD) of $\mathbf{1^H}$•OTf.

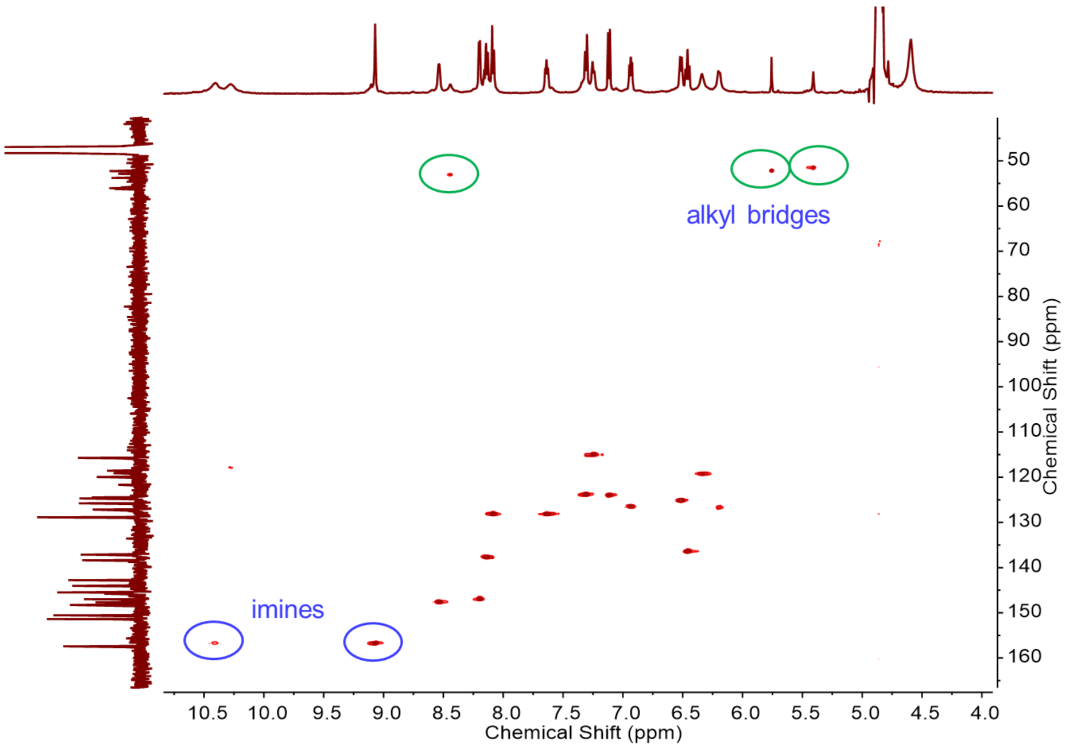

Figure S15. ^{1}H-^{13}C HSQC spectrum (500 MHz, 298 K, CD_3OD) of $\mathbf{1^H}$•OTf.

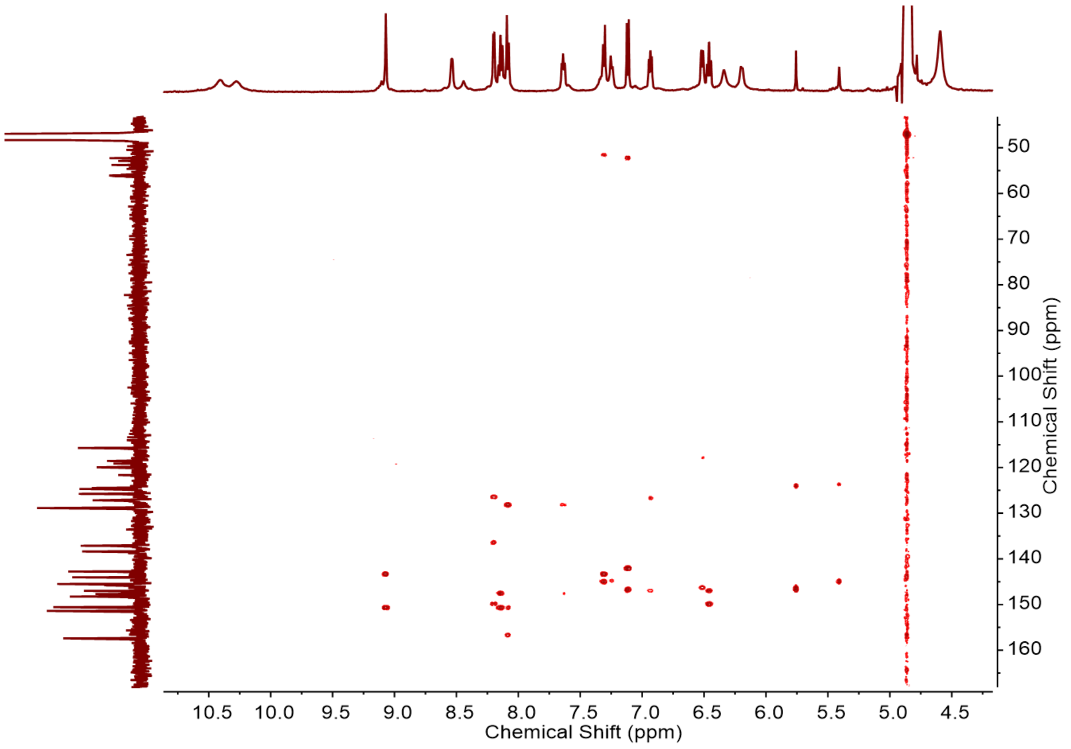

Figure S16. ^{1}H-^{13}C HMBC spectrum (500 MHz, 298 K, CD_3OD) of $\mathbf{1^H}$•OTf.

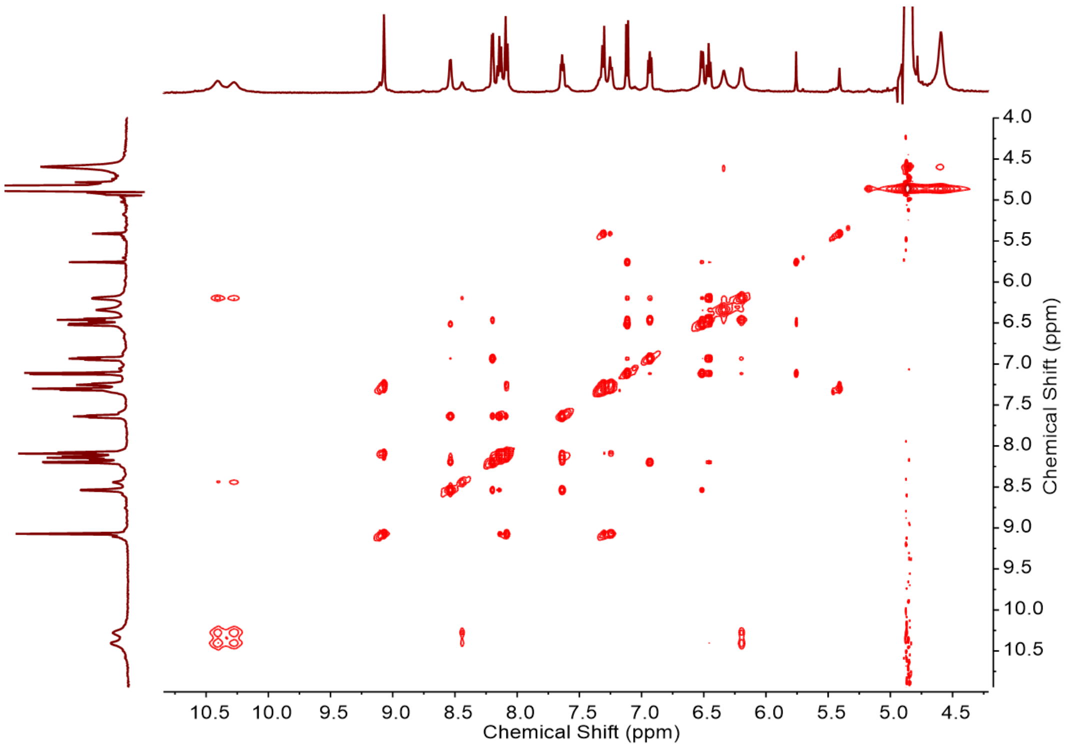

Figure S17. ^{1}H-^{1}H NOESY spectrum (500 MHz, 298 K, CD_3OD) of **1^{H}**•OTf.

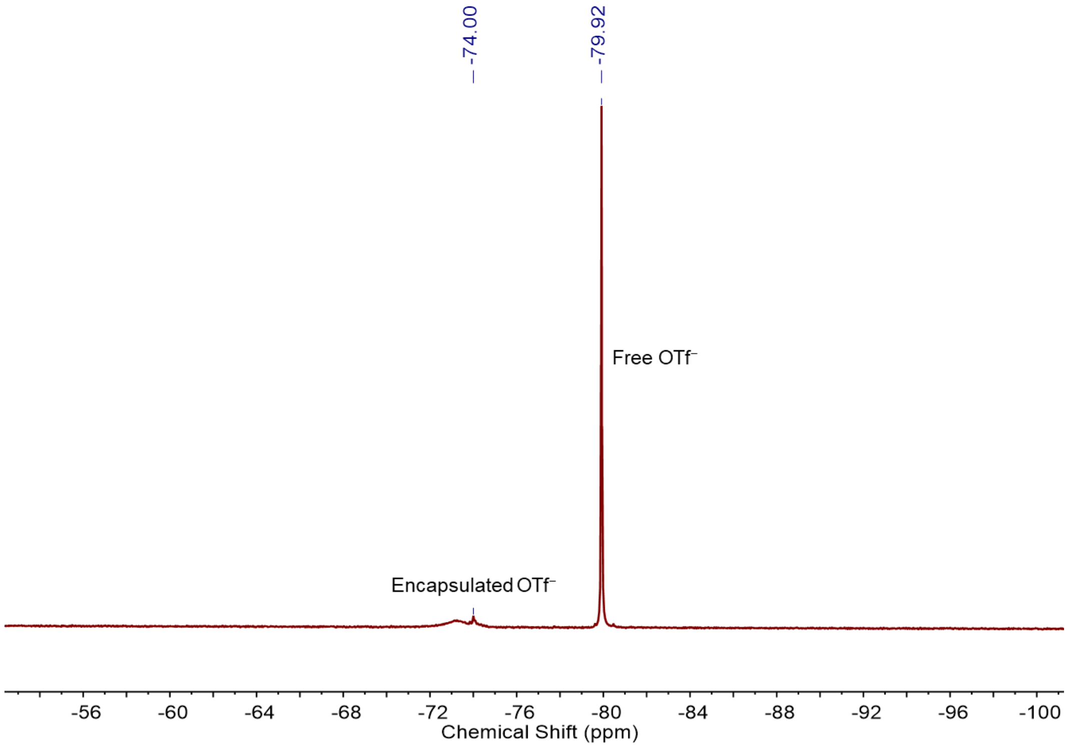

Figure S18. ^{19}F NMR spectrum (376 MHz, 298 K, CD_3OD) of **1^{H}**•OTf.

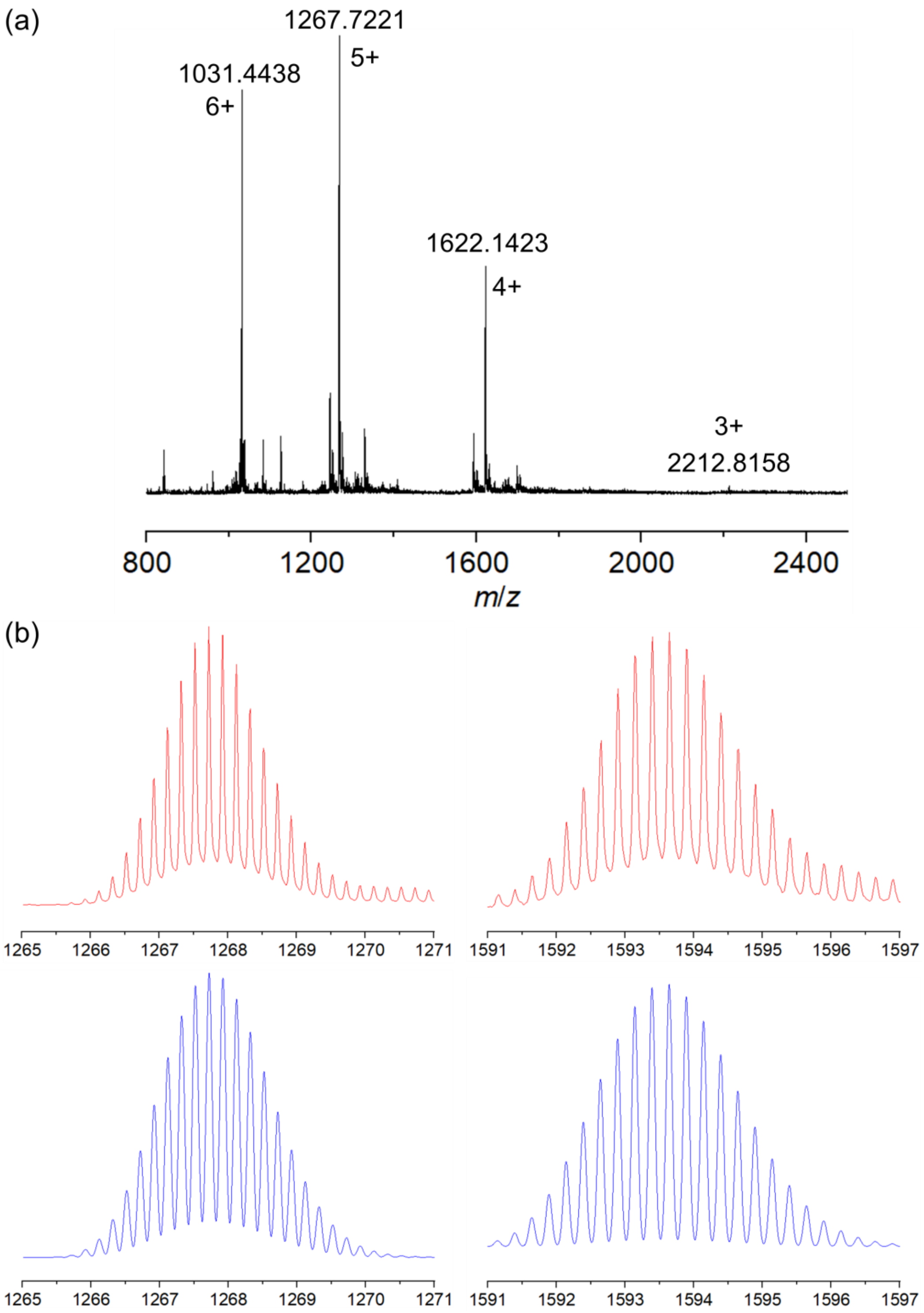

Figure S19. High resolution ESI-mass spectrum of $\mathbf{1^H}$•OTf: (a) Full spectrum and (b) selected cations $[\mathbf{1^H} + (OTf)_7]^{5+}$ (left) and $[\mathbf{1^H} + (OTf)_7Cl]^{4+}$ (right) with experimental (red) and calculated (blue) isotopic distributions. Replacement of OTf^- with Cl^- is observed. We infer that anion exchange arises from trace amounts of chloride present in the mass spectrometer. Chloride binds tightly to the cages, and thus if there is a minor chloride contamination anion exchange with chloride will occur.

5. Synthesis and characterization of **1^F^**•BF$_4$

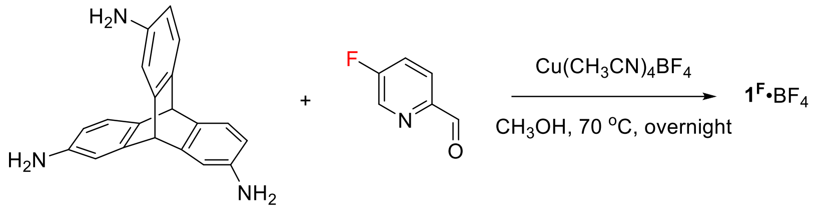

Scheme S5. Synthesis of $\mathbf{1^F}$•BF$_4$.

2,7,14-triaminotriptycene (1.00 mg, 2 equiv, 3.34 μmol), 5-fluoro-2-formylpyridine (1.25 mg, 6 equiv, 10.0 μmol), tetrakis(acetonitrile)copper(I) tetrafluoroborate (1.58 mg, 3 equiv, 5.01 μmol) and 0.70 mL of methanol were added into a small vial that was sealed in glove box. The vial was kept at 343 K overnight, affording a red solution. The solvent was reduced by nitrogen flow, followed by the addition of diethyl ether. Precipitates were collected by centrifugation and washed with ether three times. After drying in vacuum, $\mathbf{1^F}$•BF$_4$ was obtained as a deep red solid in approximately quantitative yield. ^{1}H NMR (400 MHz, 298 K, CD$_3$OD) δ 9.80 (s, 24H, containing one imine), 9.06 (s, 12H, the other imine), 8.64 (s, 12H), 8.29 – 8.16 (m, 24H), 8.10 (t, J = 8.5 Hz, 12H), 7.88 (s, 4H, alkyl bridge), 7.29 (d, J = 8.3 Hz, 24H), 7.22 (d, J = 8.2 Hz, 12H), 6.76 (d, J = 7.9 Hz, 12H), 6.60 (t, J = 8.0 Hz, 12H), 6.32 (s, 12H), 5.85 (s, 4H, alkyl bridge), 5.72 (dd, J = 8.8, 4.6 Hz, 12H), 5.39 (s, 4H, alkyl bridge), 4.82 (s, 4H, alkyl bridge). ^{13}C NMR (126 MHz, 298 K, CD$_3$OD) δ 163.3, 162.2, 161.2, 160.1, 156.6 (one imine), 155.2 (the other imine), 148.8, 148.7, 147.1, 146.8, 146.2, 146.1, 145.6, 143.7, 142.3, 136.9, 130.8, 128.4, 127.1, 125.8, 124.5, 124.0, 119.7, 116.8, 116.0, 115.6, 54.6 (alkyl bridge), 52.4 (alkyl bridge), 52.2 (alkyl bridge), 50.5 (alkyl bridge). ^{19}F NMR (471 MHz, 298 K, CD$_3$OD) δ –117.87 (s, 12F, fluoride on pyridine), –119.41 (s, 12F, fluoride on pyridine), –143.23 (encapsulated BF_4^-), –153.46 (free BF_4^-).ESI-MS: m/z = 759.08 [$\mathbf{1^F}$ + $(BF_4)_4]^{8+}$, 880.08 [$\mathbf{1^F}$ + $(BF_4)_5]^{7+}$, 1041.43 [$\mathbf{1^F}$ + $(BF_4)_6]^{6+}$, 1269.32 [$\mathbf{1^F}$ + $(BF_4)_7]^{5+}$, 1608.16 [$\mathbf{1^F}$ + $(BF_4)_8]^{4+}$.

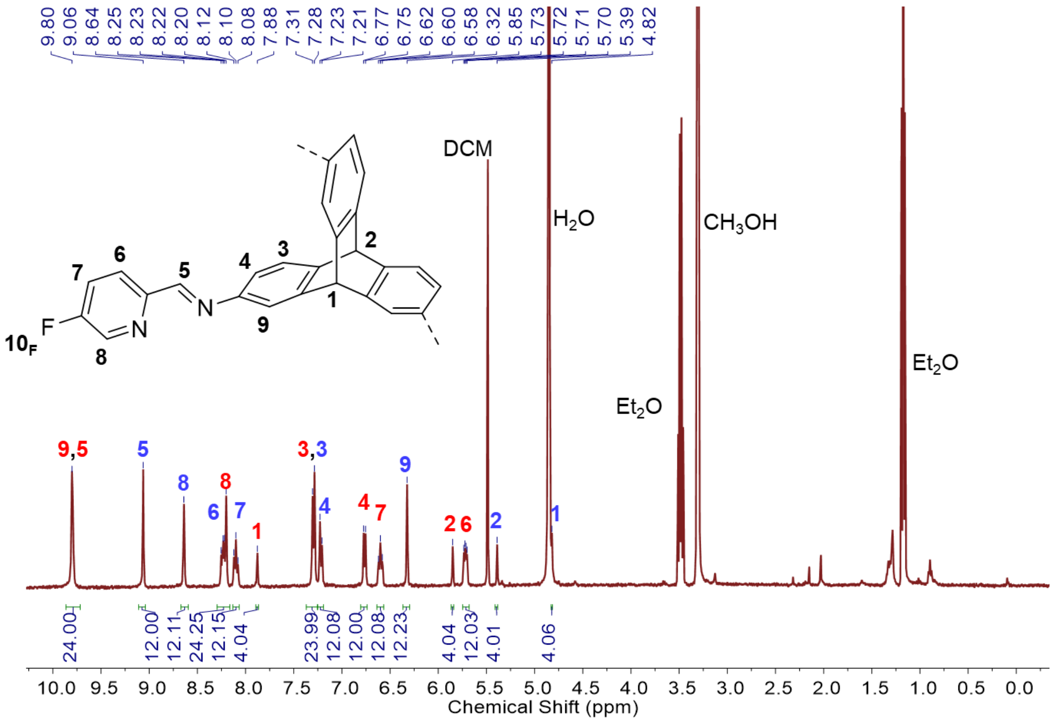

Figure S20. ^{1}H NMR spectrum (400 MHz, 298 K, CD_3OD) of $\mathbf{1^F}$•BF_4.

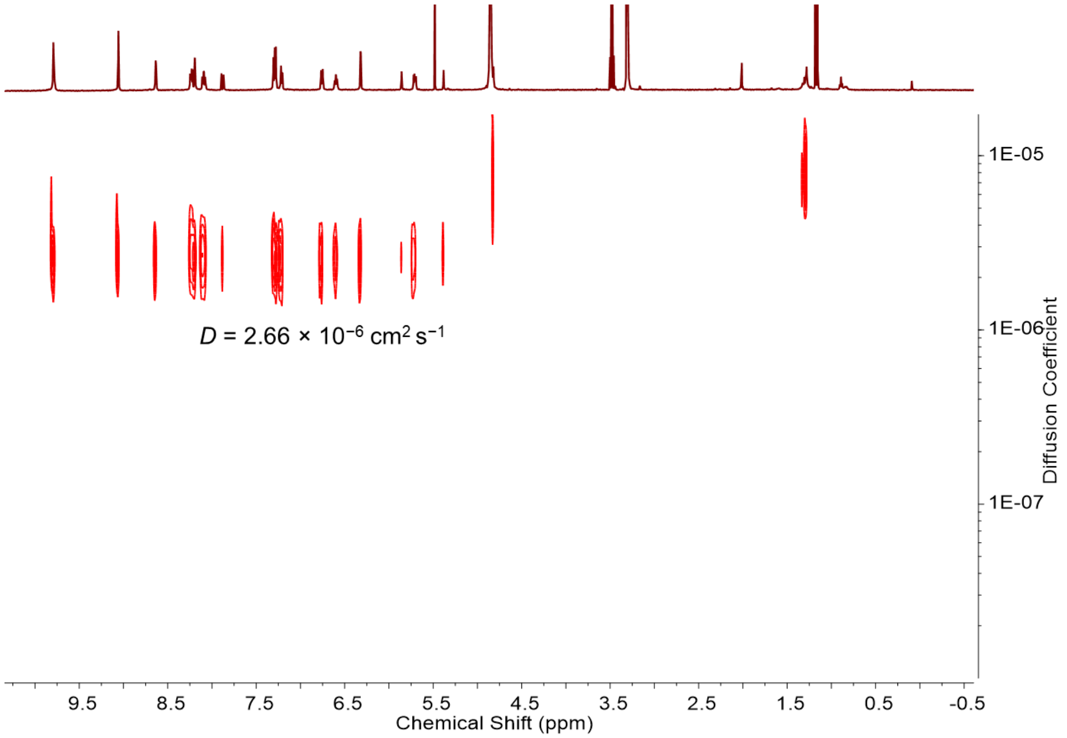

Figure S21. ^{1}H DOSY spectrum (400 MHz, 298 K, CD_3OD) of $\mathbf{1^F}$•BF_4.

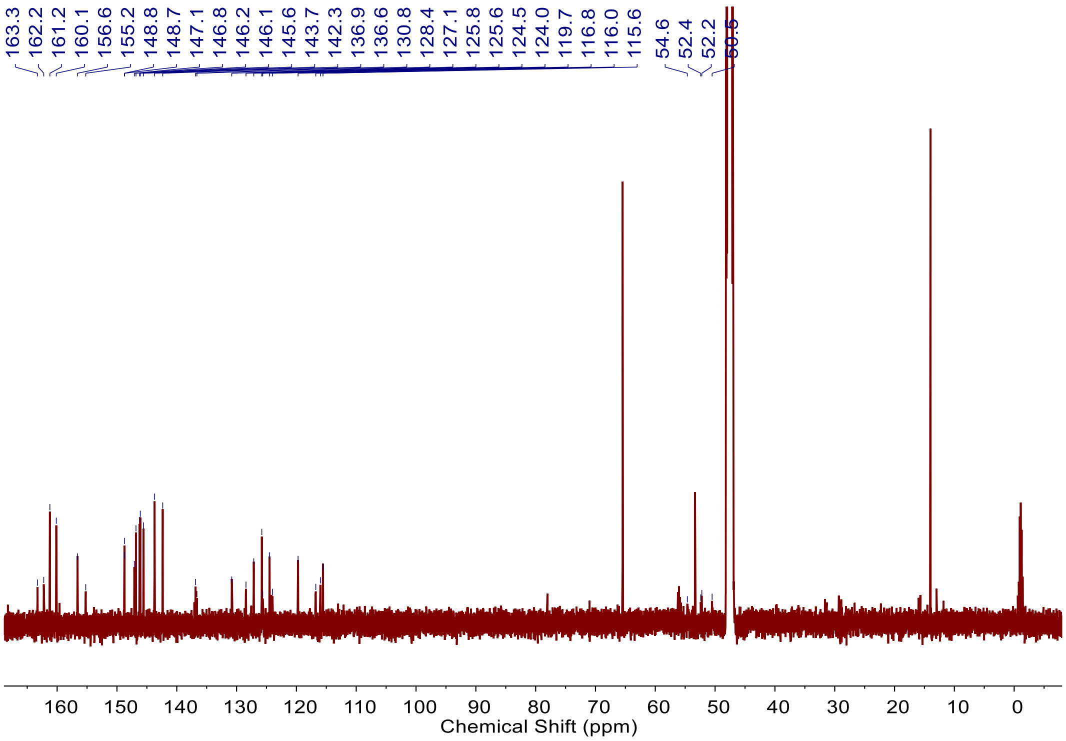

Figure S22. ^{13}C NMR spectrum (126 MHz, 298 K, CD_3OD) of $\mathbf{1^F}$•BF_4.

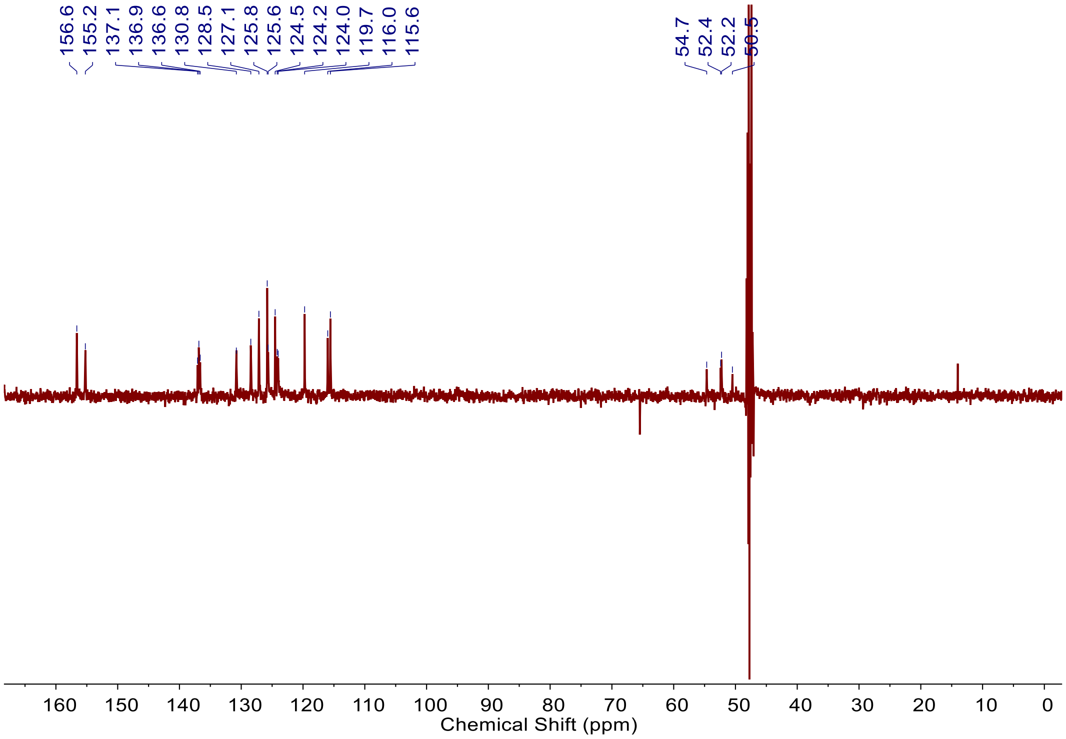

Figure S23. ^{13}C DEPT NMR spectrum (126 MHz, 298 K, CD_3OD) of $\mathbf{1^F}$•BF_4.

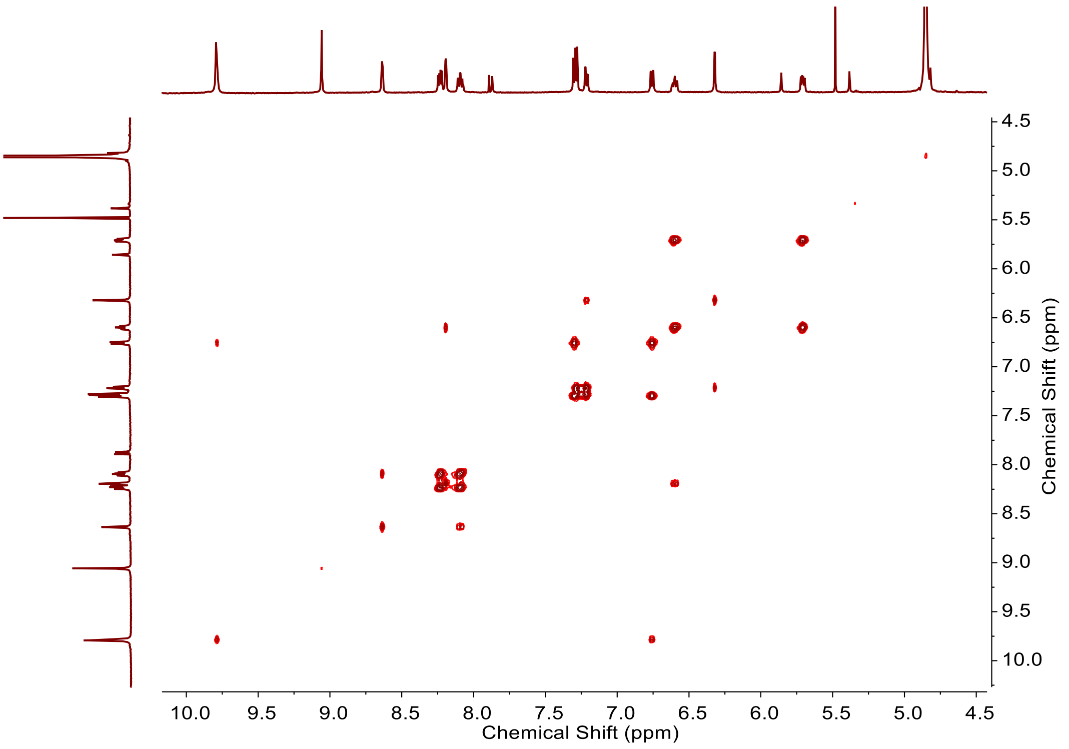

Figure S24. ^{1}H-^{1}H COSY spectrum (400 MHz, 298 K, CD_3OD) of $\mathbf{1^F}$•BF_4.

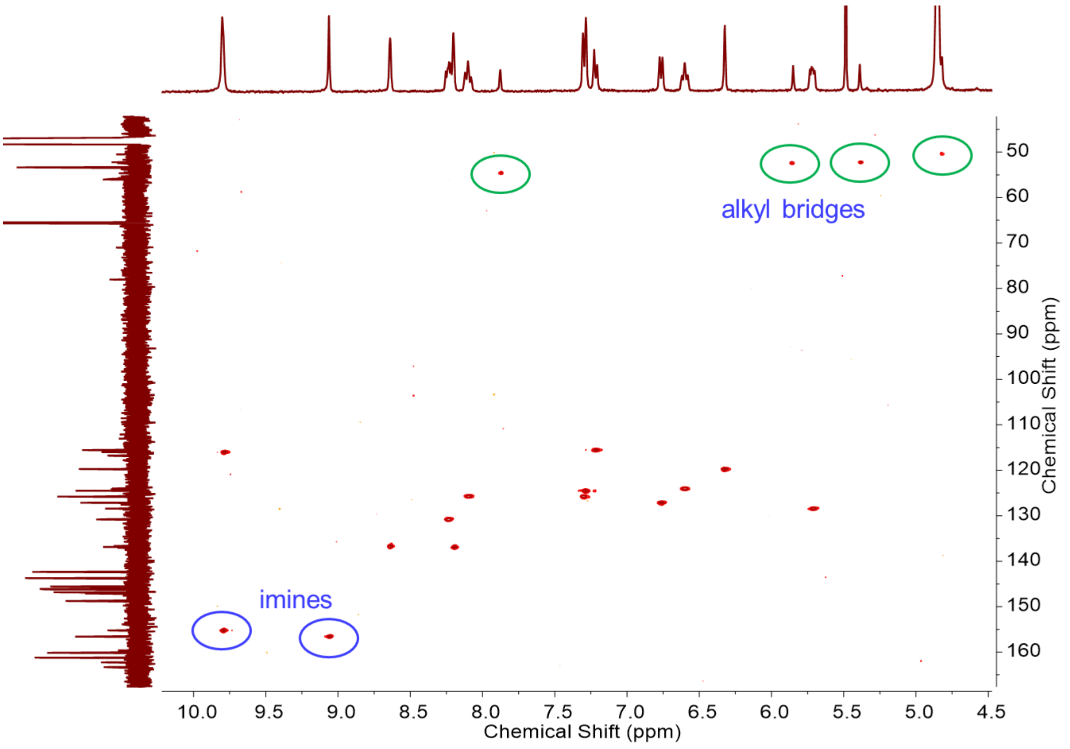

Figure S25. ^{1}H-^{13}C HSQC spectrum (400 MHz, 298 K, CD_3OD) of $\mathbf{1^F}$•BF_4.

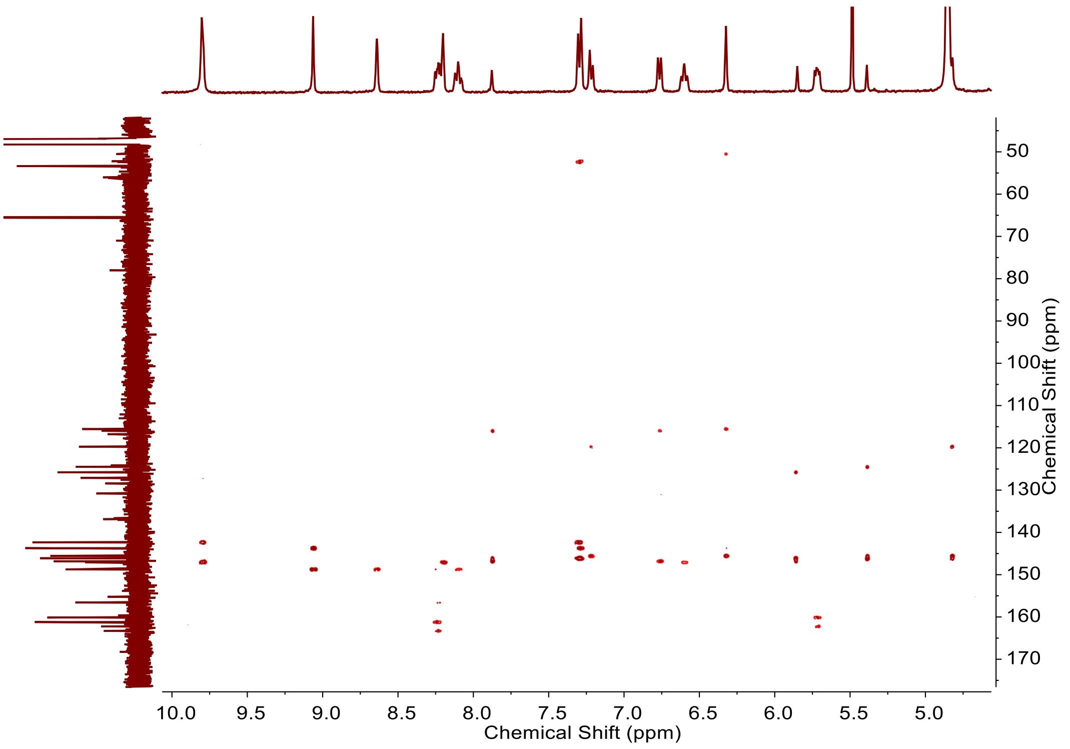

Figure S26. ^{1}H-^{13}C HMBC spectrum (400 MHz, 298 K, CD_3OD) of $\mathbf{1^F}$•BF_4.

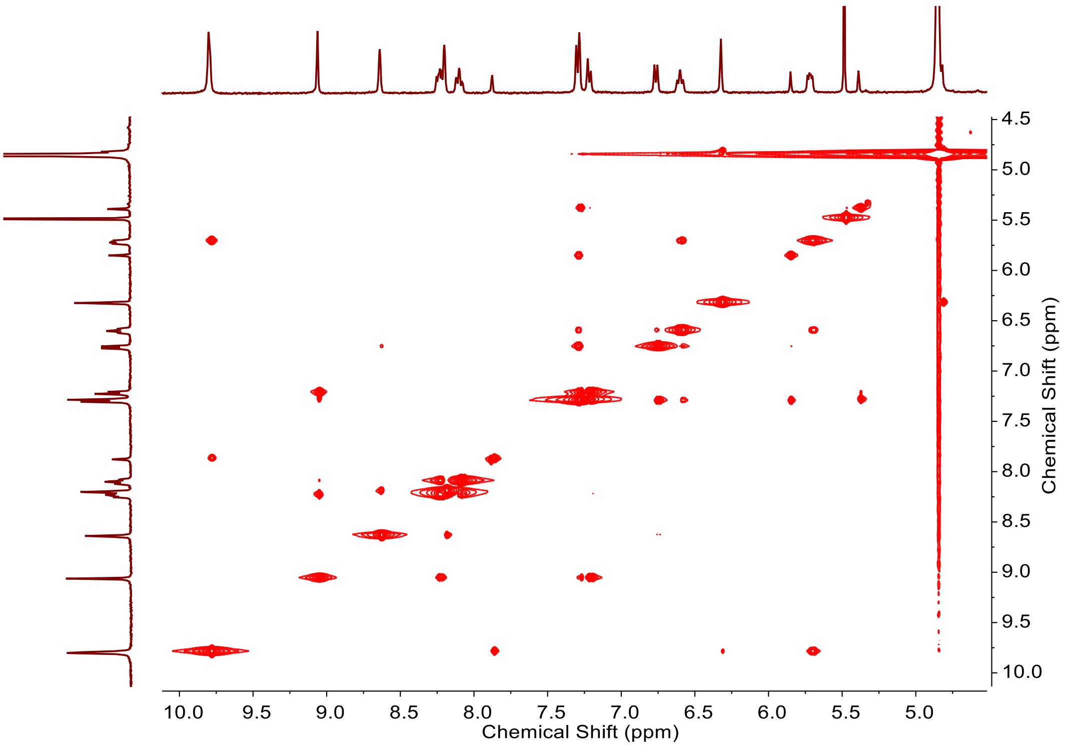

Figure S27. ^{1}H-^{1}H NOESY spectrum (500 MHz, 298 K, CD_3OD) of $\mathbf{1^F}$•BF_4.

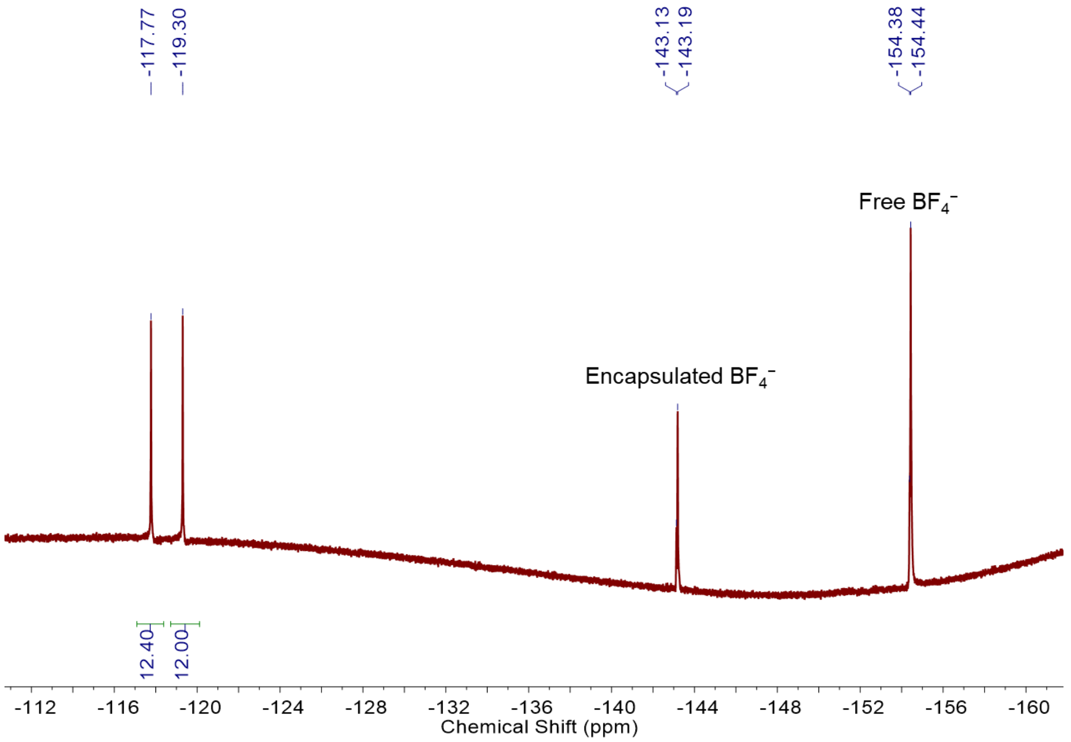

Figure S28. ^{19}F NMR spectrum (471 MHz, 298 K, CD_3OD) of $\mathbf{1^F}$•BF_4.

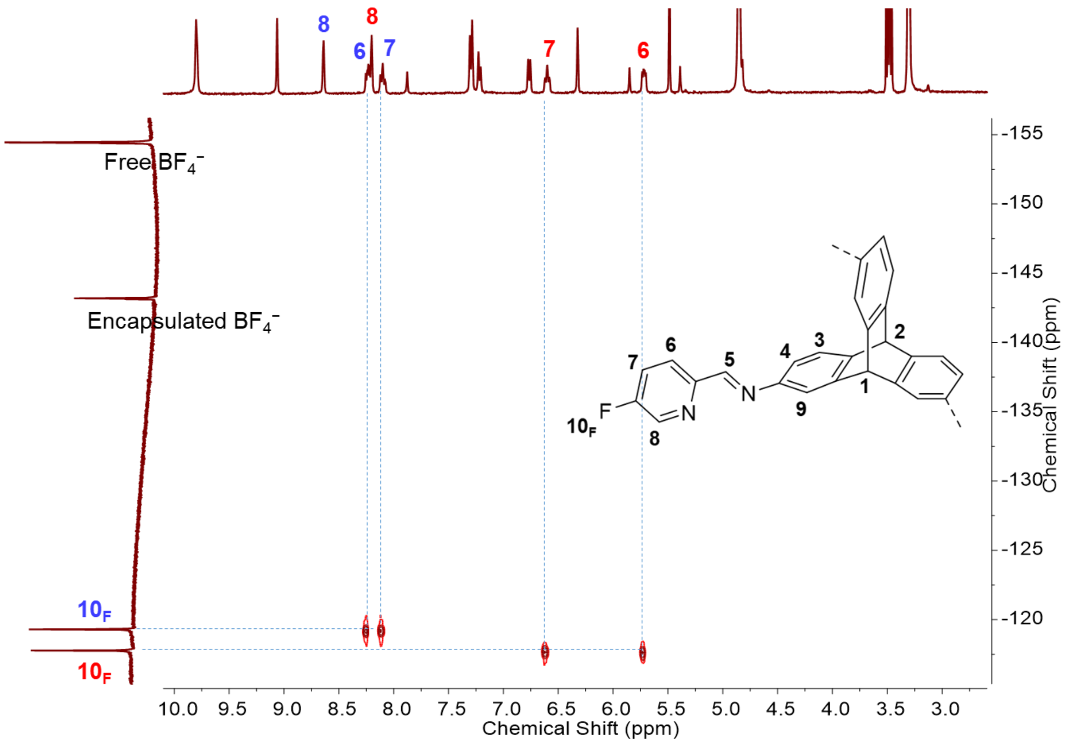

Figure S29. ^{1}H-^{19}F HMBC spectrum (500 MHz, 298 K, CD_3OD) of $\mathbf{1^F}$•BF_4. Different colours indicate distinct sets of peaks. Correlations between fluoride and protons 6 and 7 are marked with dashed lines, to help assign each peak in the ^{1}H NMR spectrum.

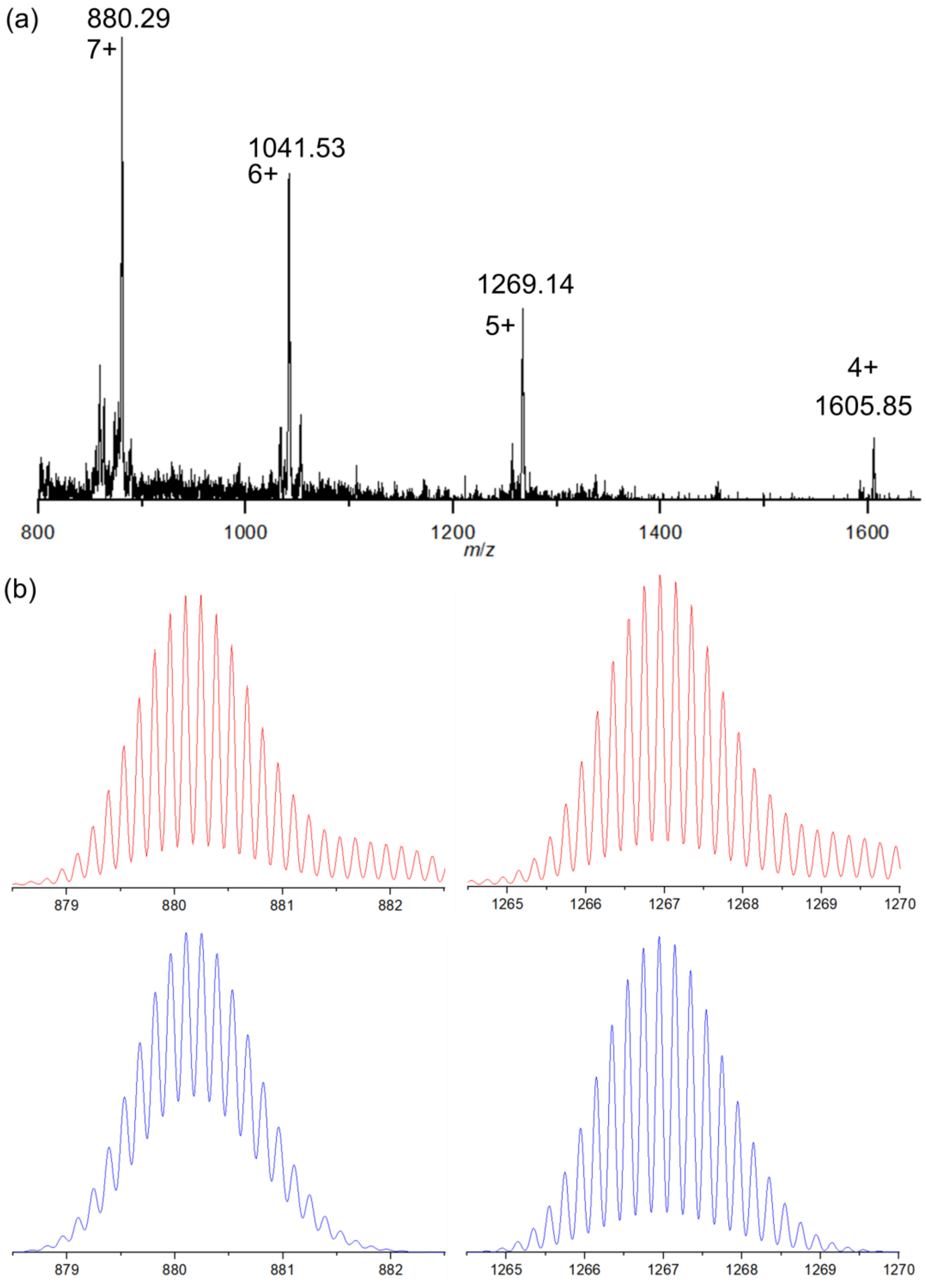

Figure S30. (a) Low-resolution ESI-mass spectrum of $\mathbf{1^F}$•BF_4. (b) Selected cations (7+, 5+, from left to right) with experimental (red) and calculated (blue) isotopic distributions from the high-resolution ESI-mass spectrum. This assembly is very fragile and was fragmentised intensively under the harsh condition of high-resolution ESI-mass spectrometer (although the condition has been modified), and thus only the low-resolution ESI-mass spectrum is shown as the full spectrum.

6. Synthesis and characterization of **1**^{F}•OTf

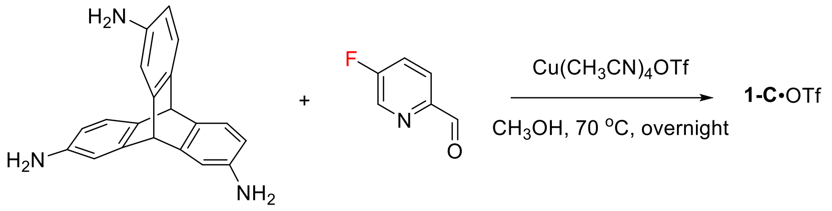

Scheme S6. Synthesis of **1**^{F}•OTf.

2,7,14-triaminotriptycene (1.00 mg, 2 equiv, 3.34 μmol), 5-fluoro-2-formylpyridine (1.25 mg, 6 equiv, 10.0 μmol), tetrakis(acetonitrile)copper(I) triflate (1.89 mg, 3 equiv, 5.01 μmol) and 0.70 mL of methanol were added into a small vial that was sealed in glove box. The vial was kept at 343 K overnight, affording a red solution. The solvent was reduced by nitrogen flow, followed by the addition of diethyl ether. Precipitates were collected by centrifugation and washed with ether three times. After drying in vacuum, **1**^{F}•OTf was obtained as a deep red solid in approximately quantitative yield. ^{1}H NMR (500 MHz, 298 K, CD_3OD) δ 10.33 (s, 12H, one imine), 10.25 (s, 12H), 9.13 (s, 12H, the other imine), 8.43 (s, 4H, alkyl bridge), 8.26 – 8.20 (m, 24H), 8.15 (s, 12H), 8.08 (td, J = 7.0, 6.2, 3.8 Hz, 12H), 7.41 – 7.36 (m, 24H), 7.33 (d, J = 8.3 Hz, 12H), 6.70 – 6.65 (m, 12H), 6.36 (td, J = 8.7, 8.1, 2.7 Hz, 12H), 6.29 (s, 12H), 6.08 (s, 12H), 6.00 (s, 4H, alkyl bridge), 5.50 (s, 4H, alkyl bridge), 4.56 (br, 4H, alkyl bridge). ^{13}C NMR (126 MHz, 298 K, CD_3OD) δ 163.3, 162.1, 161.3, 160.0, 156.7 (one imine), 155.6 (the other imine), 148.6, 148.5, 147.4, 147.3, 147.2, 147.2, 145.6, 143.6, 142.6, 137.1, 136.9, 136.4, 136.2, 130.7, 130.7, 128.1, 126.4, 125.7, 125.6, 125.4, 124.9, 123.7, 123.6, 121.6, 119.7, 119.1, 118.4, 116.1, 53.7 (alkyl bridge), 52.6 (alkyl bridge), 52.2 (alkyl bridge), 50.7 (alkyl bridge). ^{19}F NMR (471 MHz, 298 K, CD_3OD) δ –74.36 (encapsulated OTf^-), –80.01 (free OTf^-), –117.84 (br, 12F, fluoride on pyridine), –119.16 (s, 12F, fluoride on pyridine). ESI-MS: m/z = 1102.7130 [**1**^{F} + $(OTf)_6]^{6+}$, 1354.0744 [**1**^{F} + $(OTf)_7]^{5+}$, 1730.0808 [**1**^{F} + $(OTf)_8]^{4+}$, 2356.4282 [**1**^{F} + $(OTf)_9]^{3+}$.

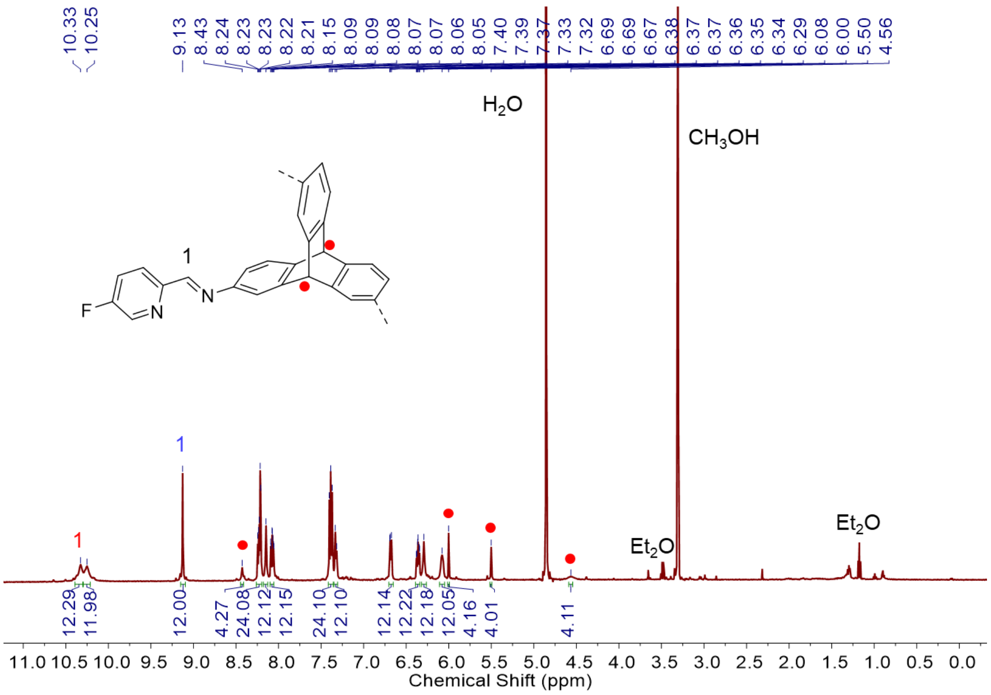

Figure S31. ^{1}H NMR spectrum (500 MHz, 298 K, CD_3OD) of $\mathbf{1^F}$•OTf. The imine signal H_1 is broad. We infer that $\mathbf{1^F}$ binds TfO^- weakly, leading to species with different numbers of bound TfO^- in solution. This multiple binding decreases the symmetry of the cage framework, resulting in different chemical environments for the imine protons. Therefore, the resonance of the imine is not a sharp singlet.

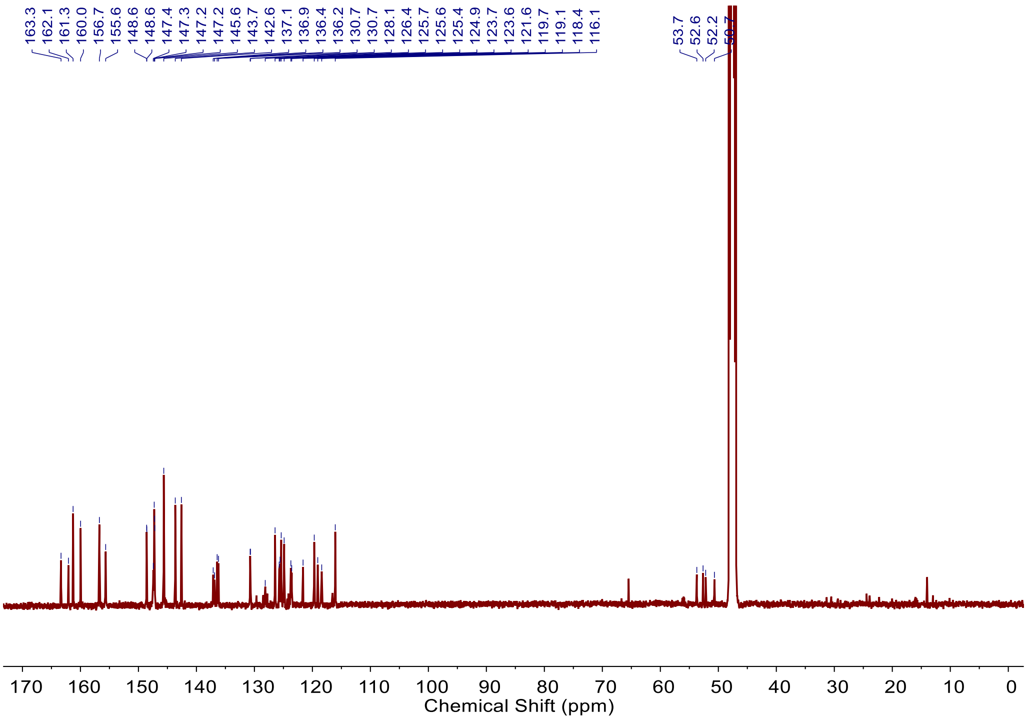

Figure S32. ^{13}C NMR spectrum (126 MHz, 298 K, CD_3OD) of $\mathbf{1^F}$•OTf.

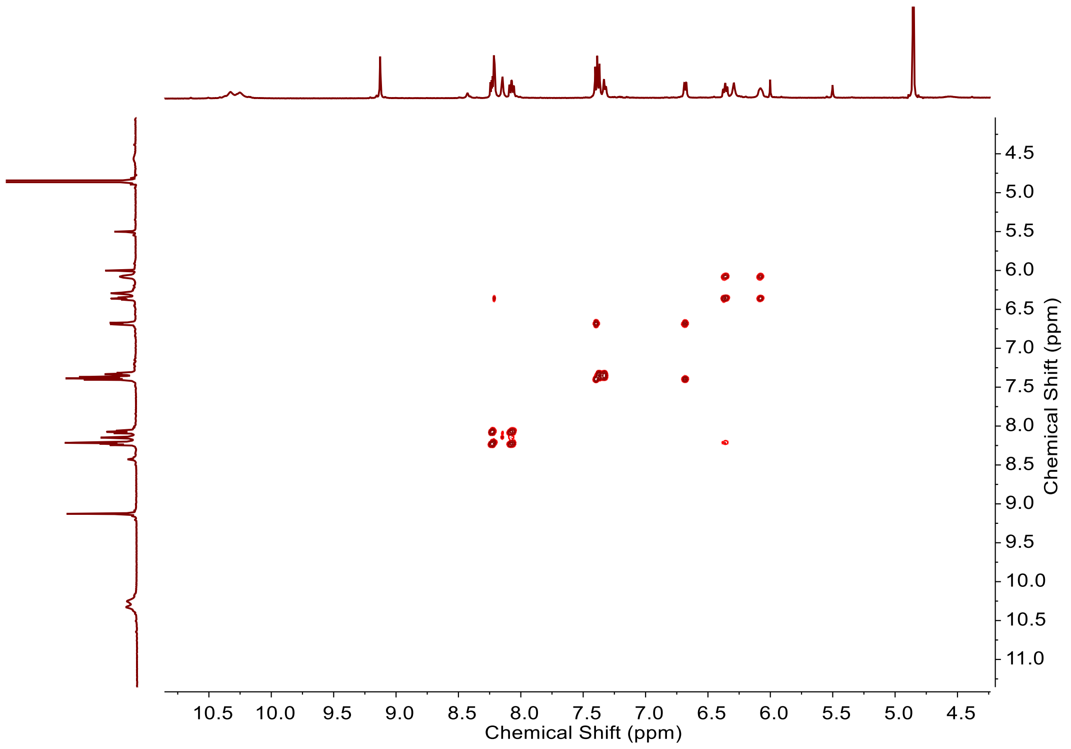

Figure S33. ^{1}H-^{1}H COSY spectrum (500 MHz, 298 K, CD_3OD) of **1^{F}**•OTf.

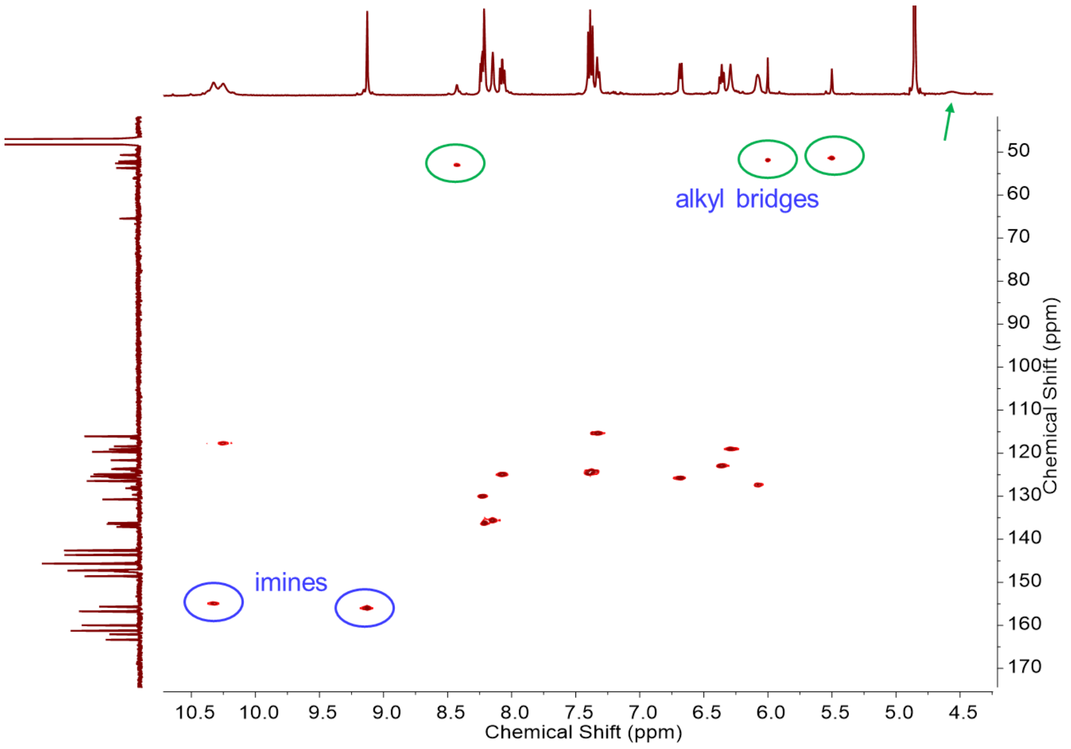

Figure S34. ^{1}H-^{13}C HSQC spectrum (500 MHz, 298 K, CD_3OD) of **1^{F}**•OTf.

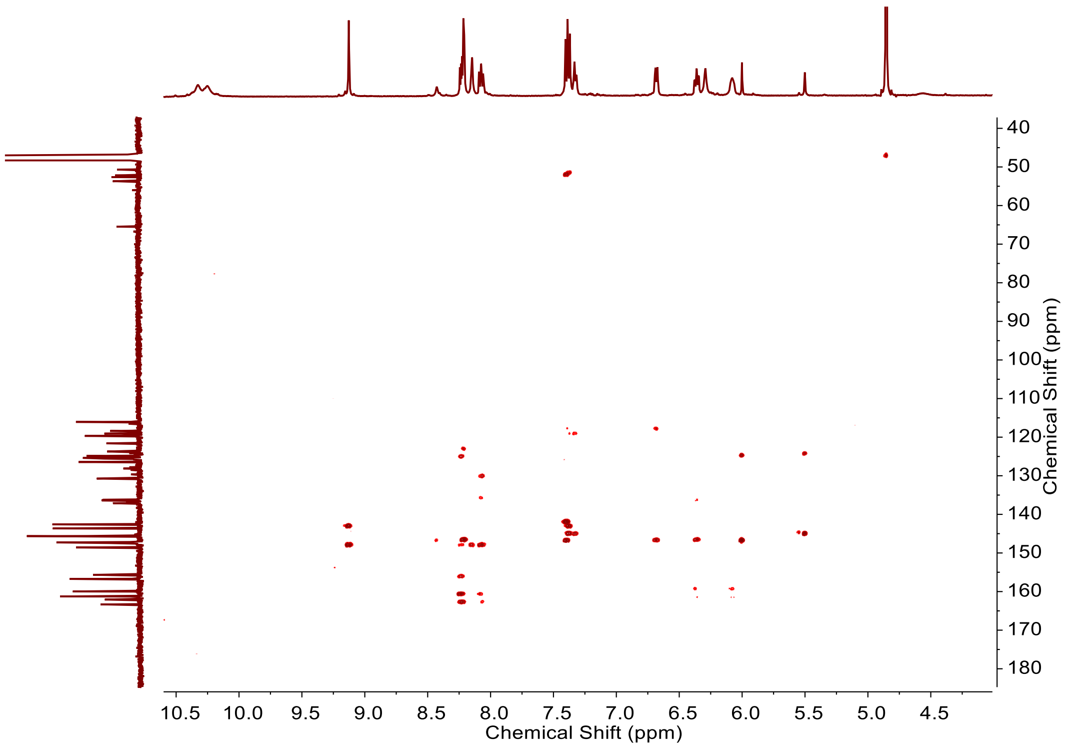

Figure S35. ^{1}H-^{13}C HMBC spectrum (500 MHz, 298 K, CD_3OD) of **1^F**•OTf.

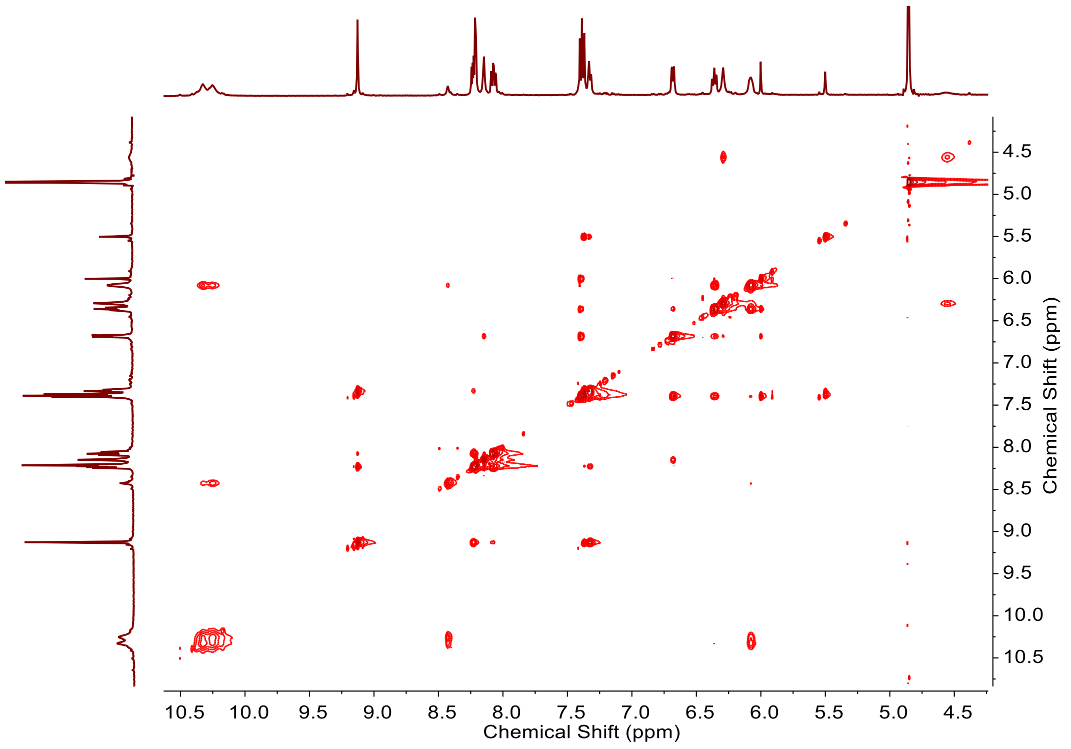

Figure S36. ^{1}H-^{1}H NOESY spectrum (500 MHz, 298 K, CD_3OD) of **1^F**•OTf.

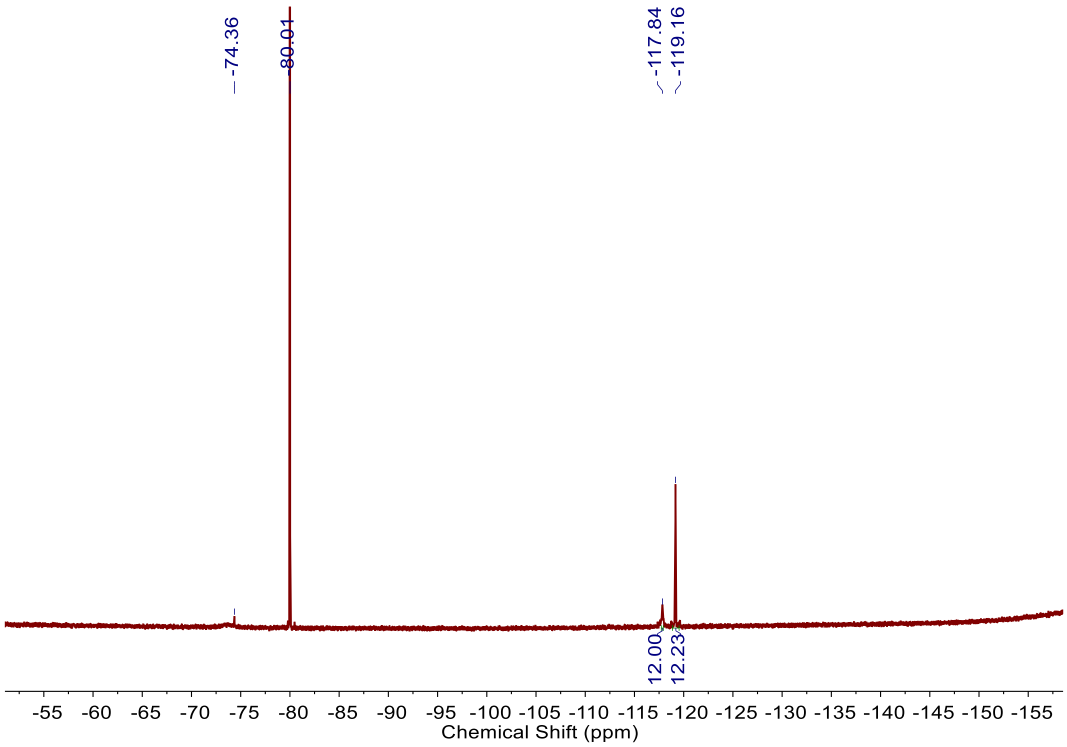

Figure S37. ^{19}F NMR spectrum (471 MHz, 298 K, CD_3OD) of $\mathbf{1^F}$•OTf.

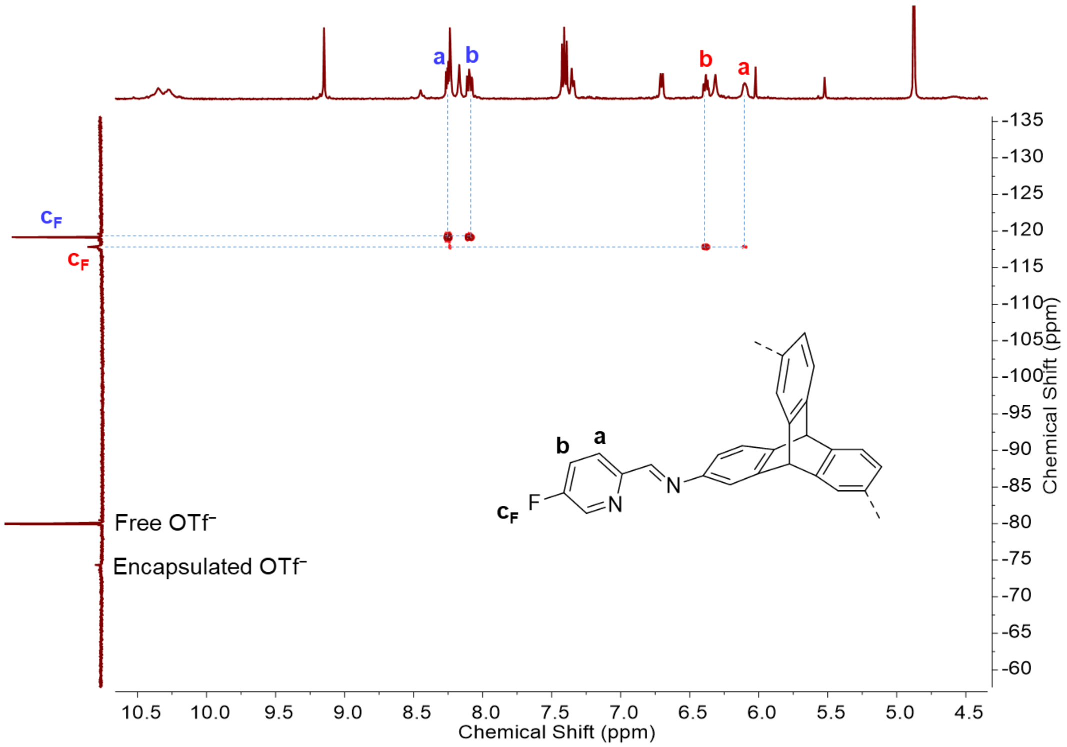

Figure S38. ^{1}H-^{19}F HMBC spectrum (500 MHz, 298 K, CD_3OD) of $\mathbf{1^F}$•OTf. Different colours indicate distinct sets of peaks. Correlations between fluoride and protons a and b are marked with dash lines, which help assigning each peak in the ^{1}H NMR spectrum.

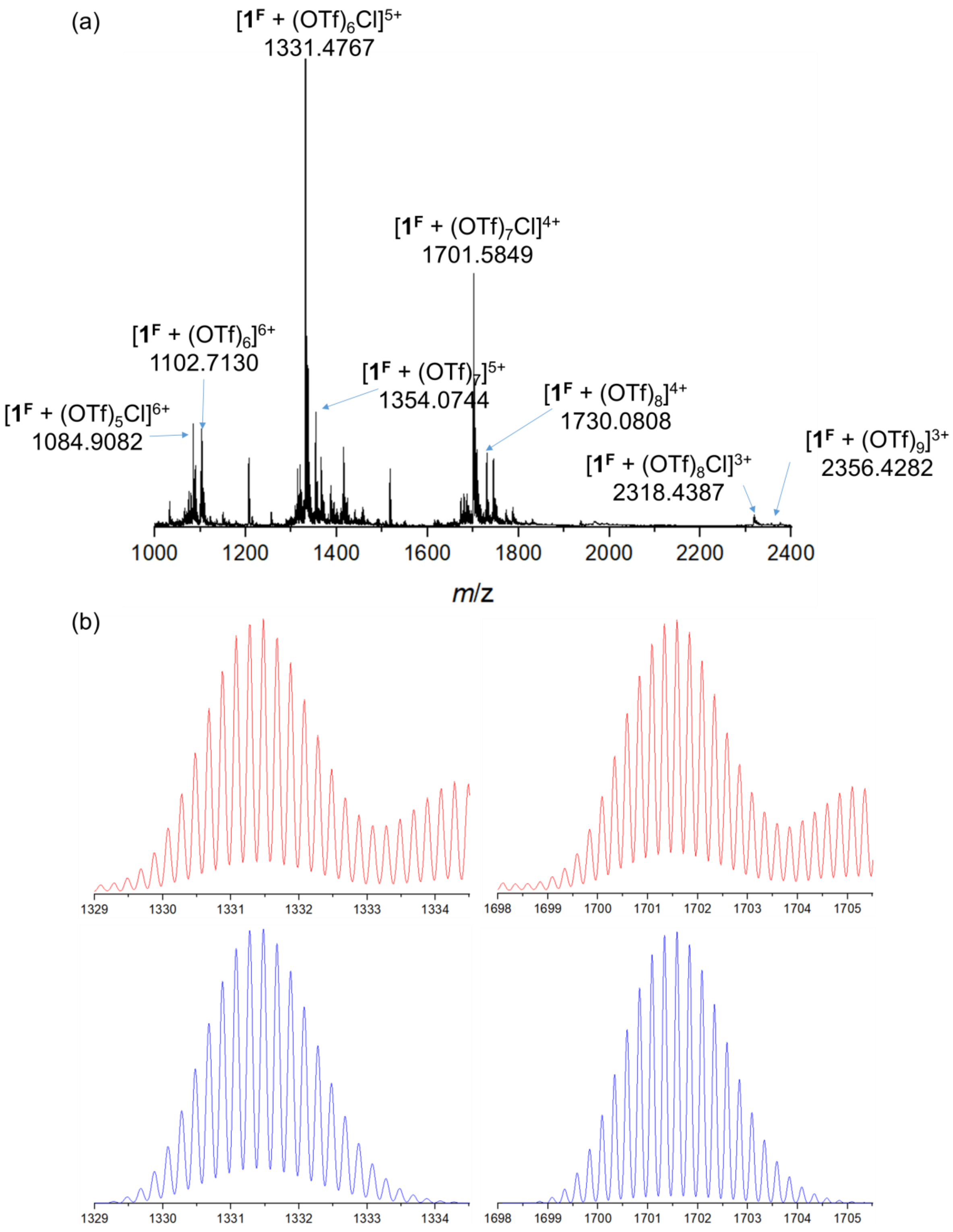

Figure S39. High-resolution ESI-mass spectrum of $\mathbf{1}^{\mathbf{F}}$•OTf: (a) Full spectrum and (b) selected cations (5+ and 4+, from left to right) with experimental (red) and calculated (blue) isotopic distributions. In the full spectrum, one OTf^{-} of the assembly was replaced by one Cl^{-}. Both signals are marked.

7. Synthesis and characterization of $\mathbf{2^{OMe}}$•BF_4

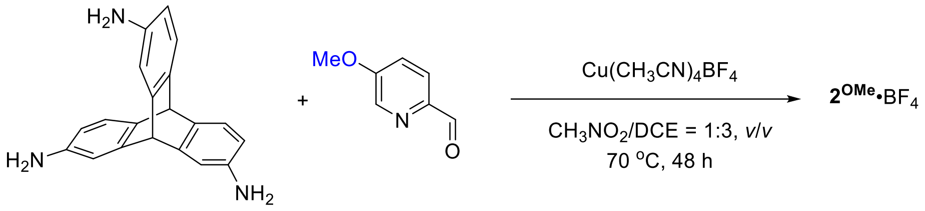

Scheme S7. Synthesis of $\mathbf{2^{OMe}}$•BF_4

2,7,14-triaminotriptycene (1.00 mg, 2 equiv, 3.34 μmol), 5-methoxy-2-formylpyridine (1.37 mg, 6 equiv, 10.0 μmol), tetrakis(acetonitrile)copper(I) tetrafluoroborate (1.58 mg, 3 equiv, 5.01 μmol), 0.20 mL of nitromethane and 0.60 mL of 1,2$^{\mathbf{OMe}}$ichloroethane were added into a small vial that was sealed in glove box. The vial was kept at 343 K for 48 h, affording a red-orange solution. The solvent was reduced by vacuum, followed by the addition of diethyl ether. Precipitates were collected by centrifugation and washed with ether three times. After drying in vacuum, $\mathbf{2^{OMe}}$•BF_4 was obtained as a red-orange solid in approximately quantitative yield. ^{1}H NMR (500 MHz, 298 K, CD_3NO_2) δ 10.28 (s, 6H, the first imine), 9.90 (d, J = 2.3 Hz, 6H), 9.70 (s, 6H, the second imine), 8.91 (s, 6H, the third imine), 8.71 (s, 6H, the fourth imine), 8.55 (d, J = 2.7 Hz, 6H), 8.31 (d, J = 2.8 Hz, 6H), 8.27 – 8.25 (m, 6H), 8.13 (dd, J = 5.8, 3.0 Hz, 12H), 7.96 (d, J = 8.8 Hz, 6H), 7.84 (d, J = 8.8 Hz, 6H), 7.81 (s, 2H, alkyl bridge), 7.76 – 7.68 (m, 24H), 7.52 (d, J = 7.9 Hz, 6H), 7.25 (dd, J = 7.8, 2.2 Hz, 6H), 7.22 (s, 2H, alkyl bridge), 7.21 – 7.17 (m, 14H), 7.07 (d, J = 2.0 Hz, 6H), 7.00 (d, J = 8.6 Hz, 6H), 6.92 (d, J = 7.8 Hz, 6H), 6.76 (dd, J = 7.8, 2.1 Hz, 6H), 6.53 (s, 2H, alkyl bridge), 6.04 (d, J = 2.0 Hz, 6H), 5.82 (dd, J = 8.9, 2.8 Hz, 6H), 5.48 (s, 6H), 4.80 (s, 6H), 4.27 – 4.25 (m, 6H), 3.87 (s, 18H, methyoxyl group), 3.84 (s, 2H, alkyl bridge), 3.75 (s, 18H, methyoxyl group), 3.65 (s, 18H, methyoxyl group), 2.83 (s, 18H, methyoxyl group). Note that some protons of the alkyl bridge are missing in the spectrum, which could be ascribed to the broadened signal or burying in other peaks. This assembly has been confirmed by its single crystal structure. ^{13}C NMR (126 MHz, 298 K, CD_3NO_2) δ 160.2, 160.0, 159.8, 159.5, 158.8, 154.8, 154.3, 153.9, 148.1, 147.5, 146.3, 145.7, 145.0, 145.0, 144.7, 144.4, 143.7, 142.9, 142.8, 141.8, 141.6, 129.7, 129.2, 128.7, 128.2, 126.0, 125.8, 125.4, 124.9, 124.5, 121.1, 119.8, 119.2, 118.8, 118.6, 117.1, 116.6, 115.2, 114.0, 112.6, 56.6, 55.9, 55.9, 55.6, 53.3, 51.6. ^{19}F NMR (376 MHz, 298 K, CD_3NO_2) δ –146.81 (encapsulated BF_4^-), –151.55 (free BF_4^-). ESI-MS: m/z = 921.4480 [$\mathbf{2^{OMe}}$ + $(BF_4)_5$]$^{7+}$, 1089.5237 [$\mathbf{2^{OMe}}$ + $(BF_4)_6$]$^{6+}$, 1324.6297 [$\mathbf{2^{OMe}}$ + $(BF_4)_7$]$^{5+}$, 1677.5400 [$\mathbf{2^{OMe}}$ + $(BF_4)_8$]$^{4+}$, 2265.7111 [$\mathbf{2^{OMe}}$ + $(BF_4)_9$]$^{3+}$.

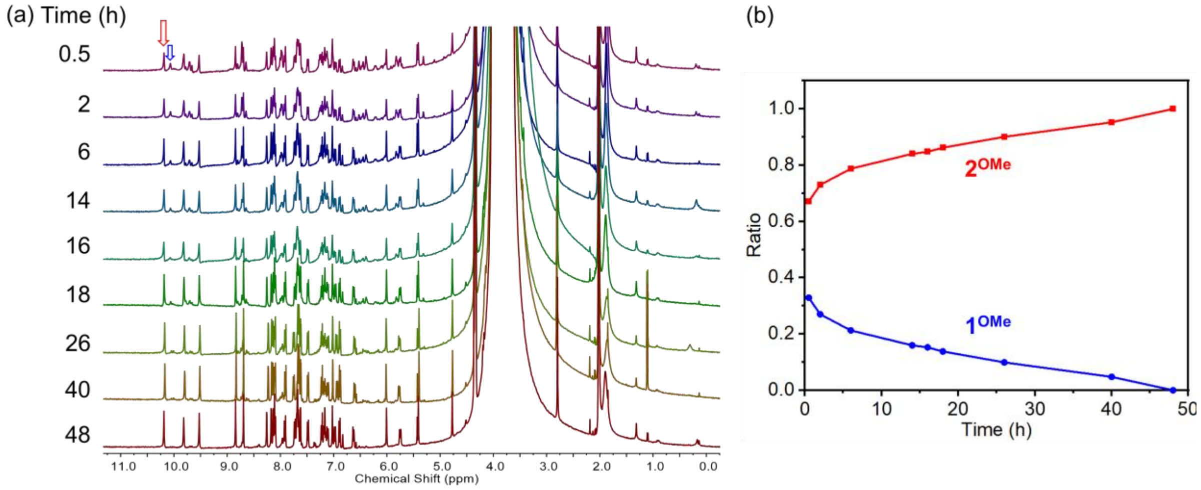

Figure S40. (a) Time-dependent ^{1}H NMR spectra (400 MHz, 298 K, CD_3NO_2/DCE = 1:3, *v*/*v*) of the preparation of $\mathbf{2^{OMe}}$•BF_4. The huge peak at 3–4.5 ppm is from non-deuterated DCE. The heating time is shown on the left. (b) Ratios of $\mathbf{1^{OMe}}$ and $\mathbf{2^{OMe}}$ versus reaction time. The ratios were determined by NMR integration. The peaks used for integration were marked with arrows ($\mathbf{1^{OMe}}$, blue; $\mathbf{2^{OMe}}$, red) in (a).

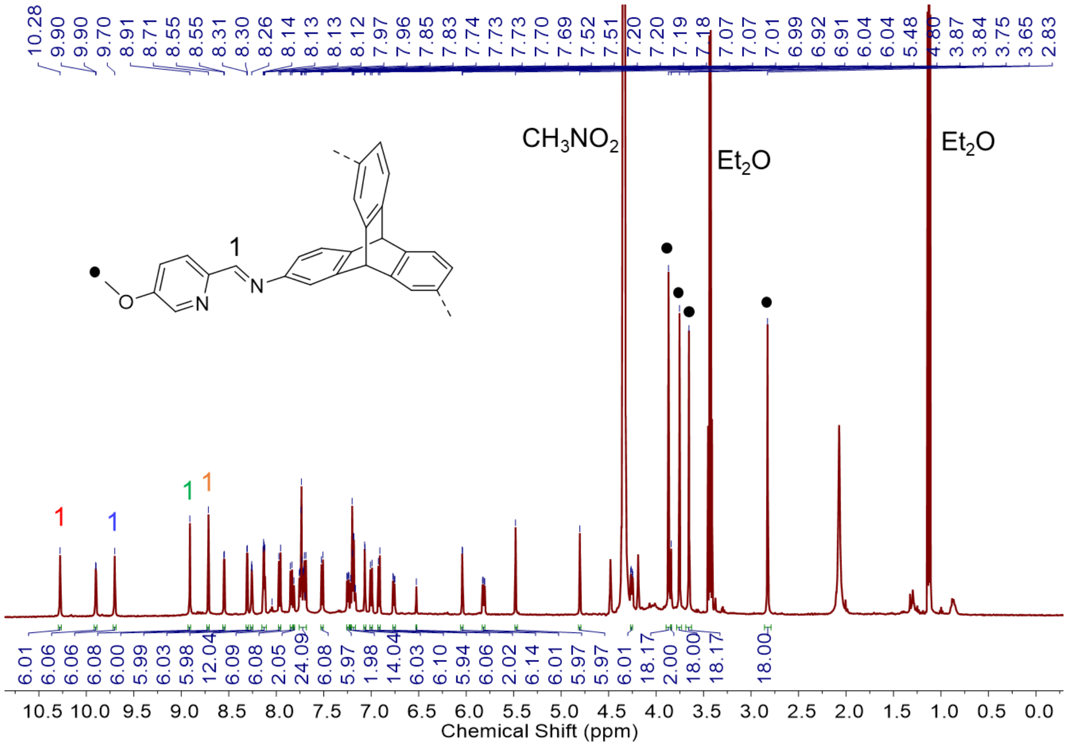

Figure S41. ^{1}H NMR spectrum (500 MHz, 298 K, CD_3NO_2) of $\mathbf{2^{OMe}}$•BF_4.

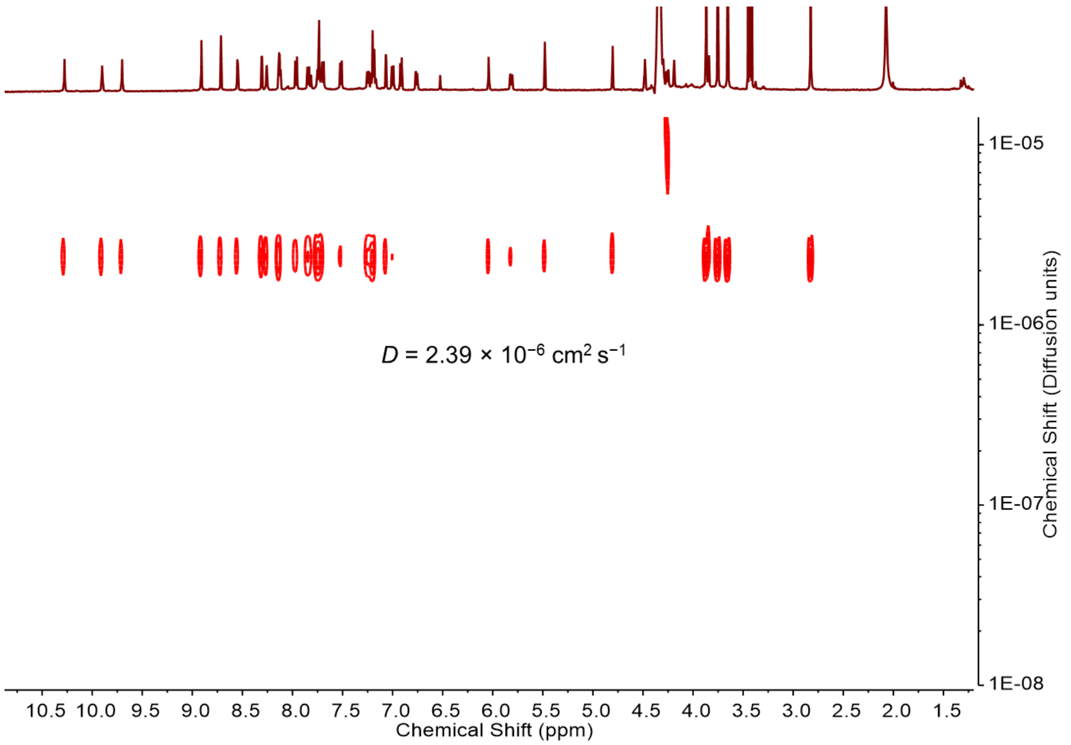

Figure S42. DOSY spectrum (400 MHz, 298 K, CD_3NO_2) of $\mathbf{2^{OMe}}$•BF_4.

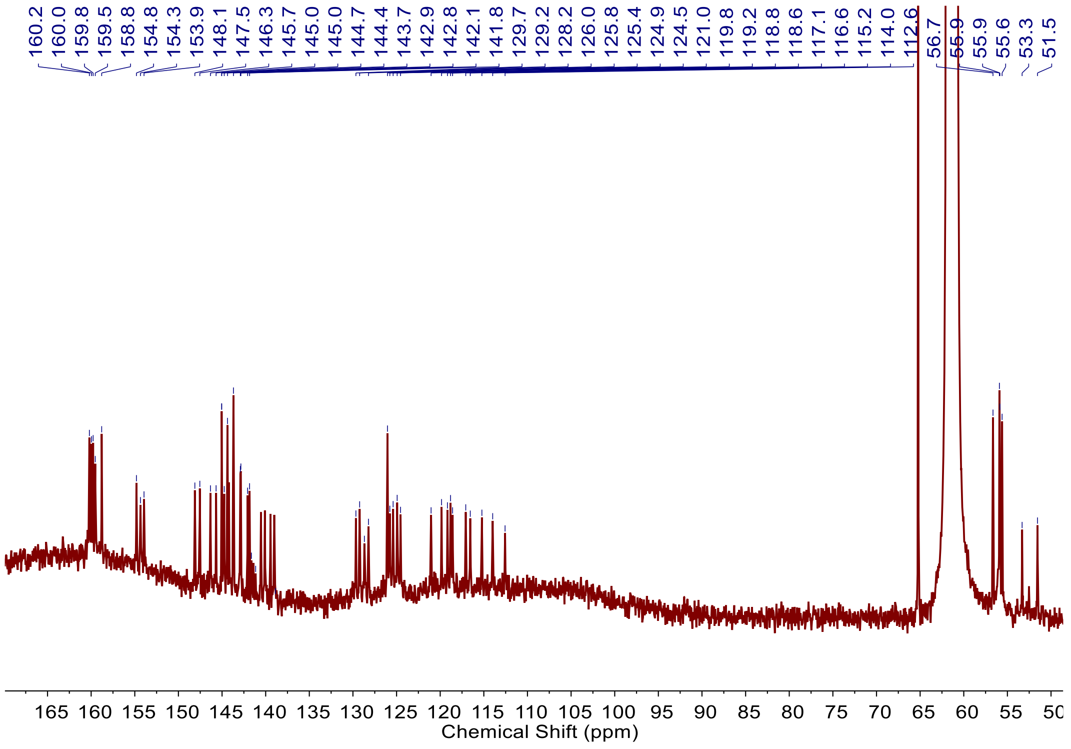

Figure S43. ^{13}C NMR spectrum (126 MHz, 298 K, CD_3NO_2) of $\mathbf{2^{OMe}}$•BF_4.

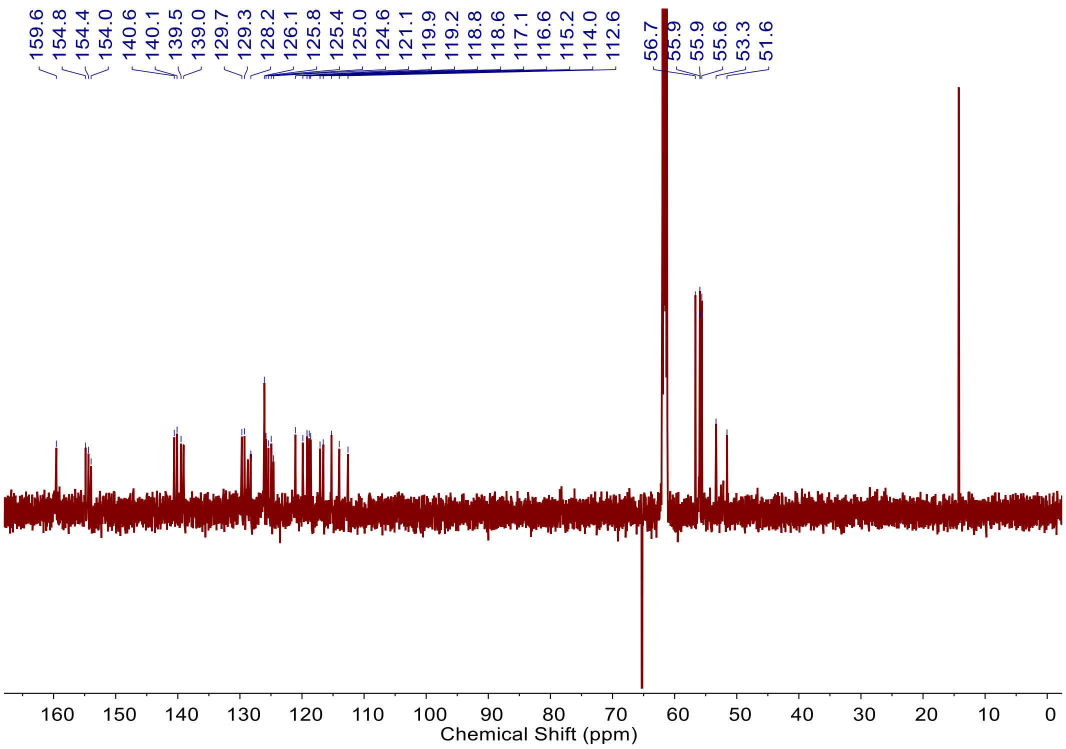

Figure S44. ^{13}C NMR dept spectrum (126 MHz, 298 K, CD_3NO_2) of $\mathbf{2}^{\mathbf{OMe}}$•$BF_4$.

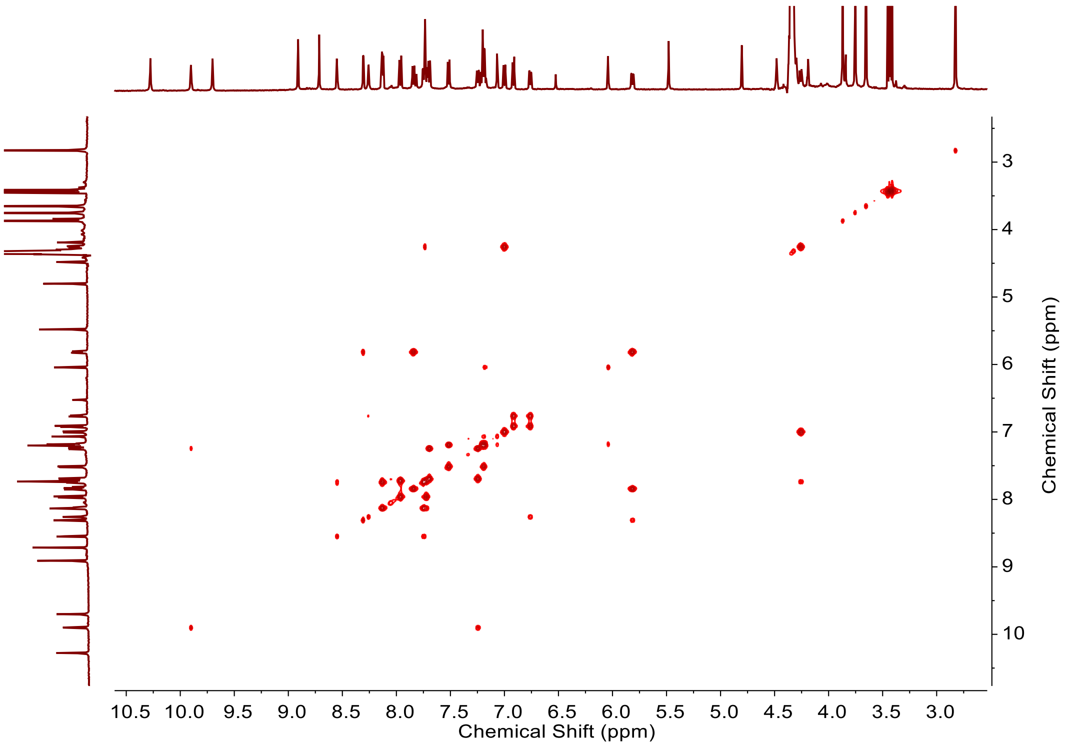

Figure S45. ^{1}H-^{1}H COSY spectrum (500 MHz, 298 K, CD_3NO_2) of $\mathbf{2}^{\mathbf{OMe}}$•$BF_4$.

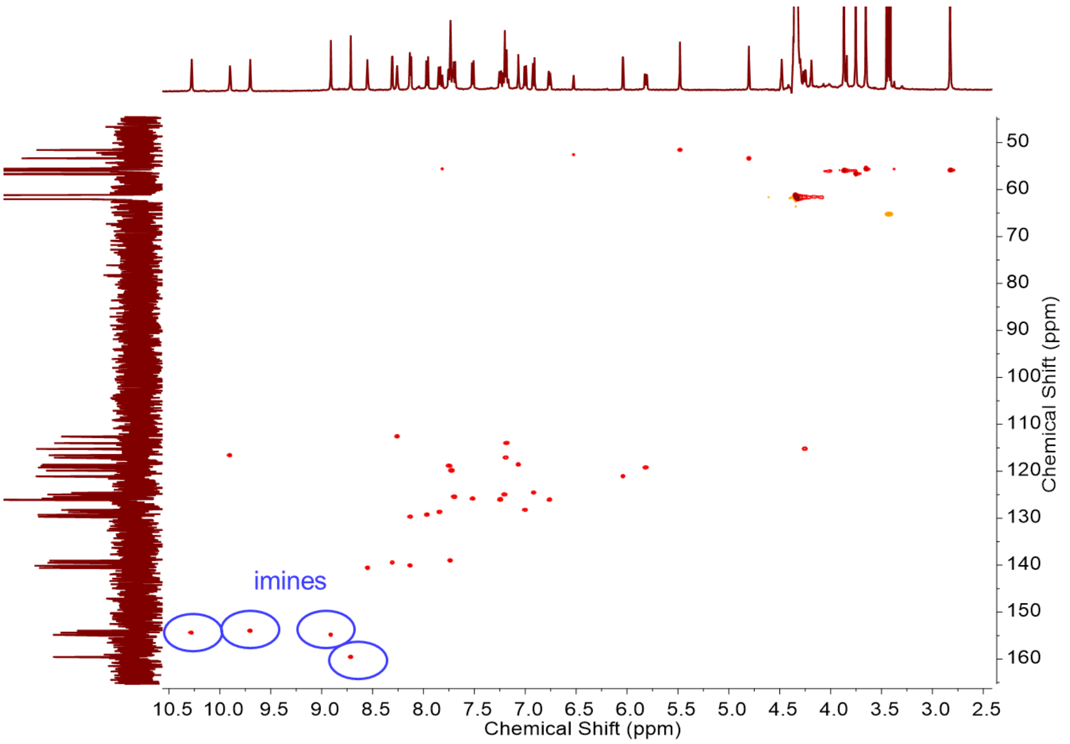

Figure S46. ^{1}H-^{13}C HSQC spectrum (500 MHz, 298 K, CD_3NO_2) of **2^{OMe}**•BF_4.

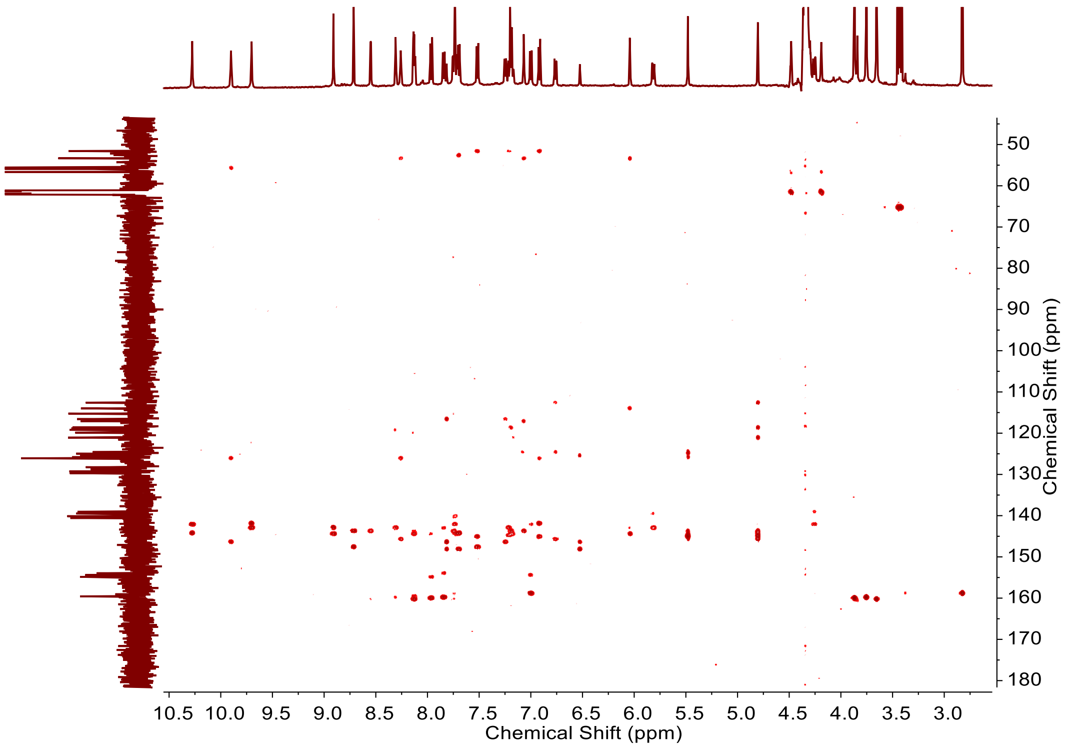

Figure S47. ^{1}H-^{13}C HMBC spectrum (500 MHz, 298 K, CD_3NO_2) of **2^{OMe}**•BF_4.

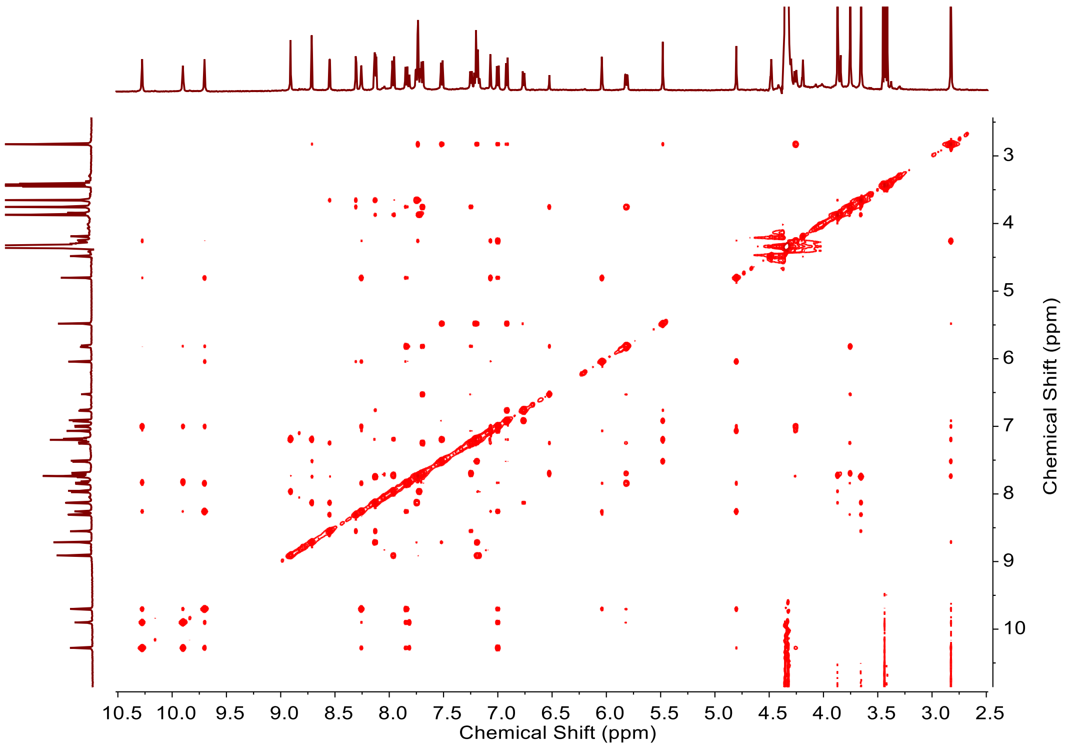

Figure S48. ^{1}H-^{1}H NOESY spectrum (500 MHz, 298 K, CD_3NO_2) of $\mathbf{2^{OMe}}$•BF_4.

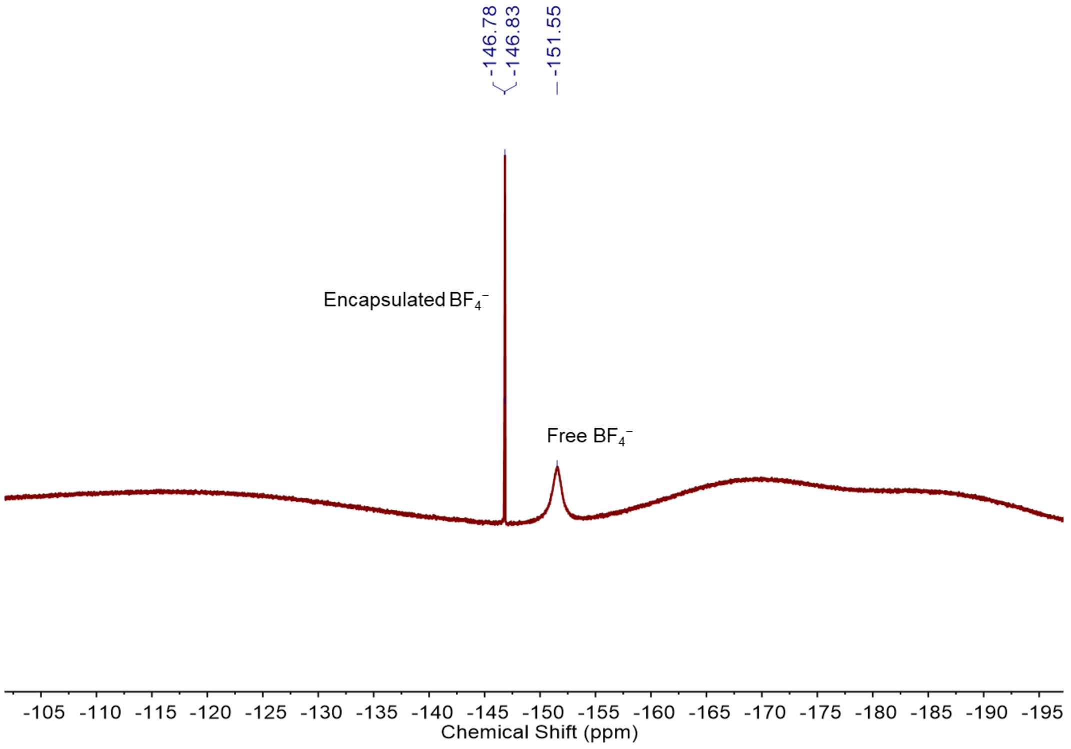

Figure S49. ^{19}F NMR spectrum (376 MHz, 298 K, CD_3NO_2) of $\mathbf{2^{OMe}}$•BF_4.

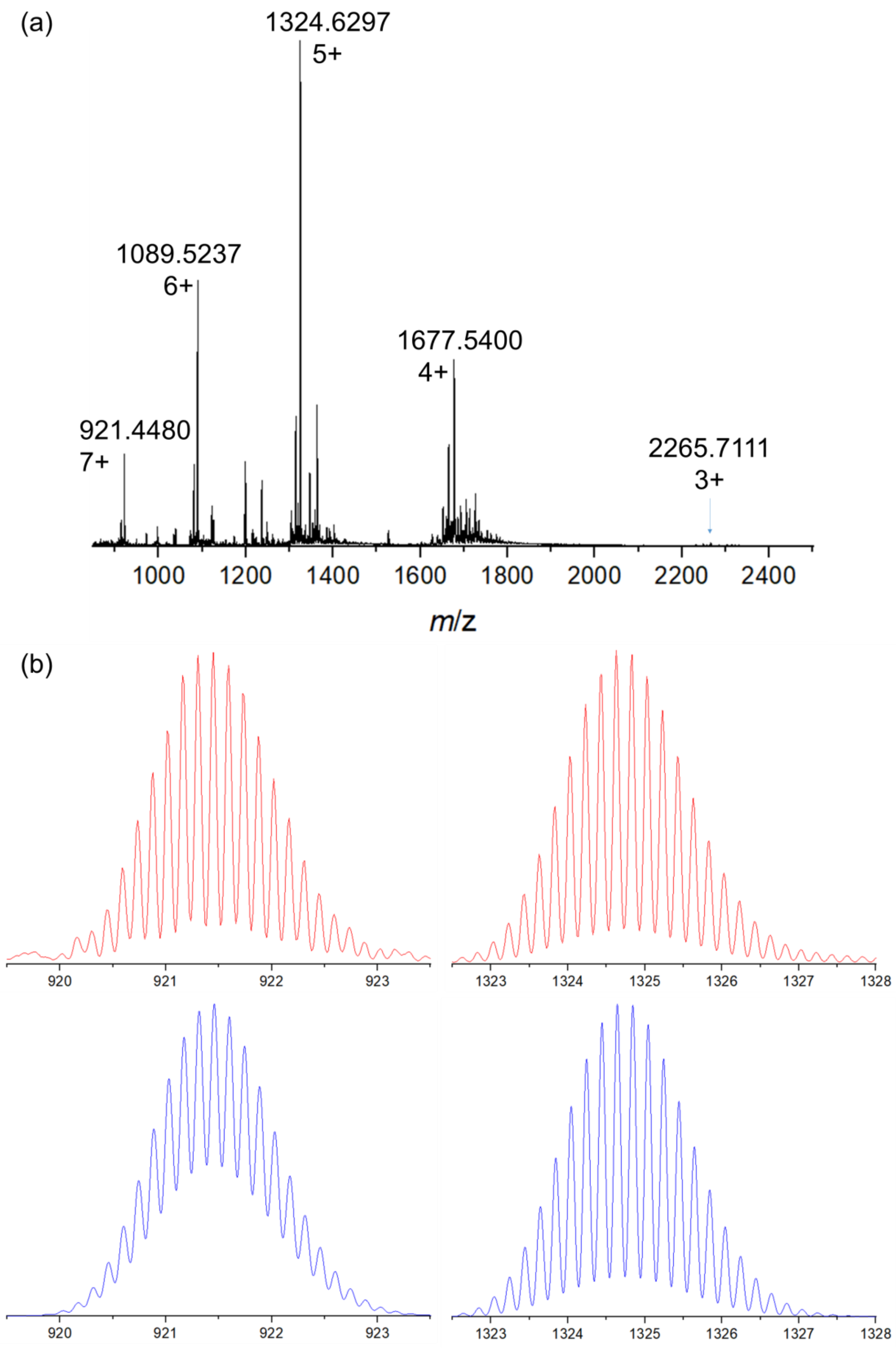

Figure S50. High-resolution ESI-mass spectrum of $\mathbf{2}^{\mathbf{OMe}}$•$BF_4$: (a) Full spectrum and (b) selected cations (7+ and 5+, from left to right) with experimental (red) and calculated (blue) isotopic distributions. In the full spectrum, one BF_4^- of the assembly was replaced by one Cl^-; these peaks appeared on the left of the main peaks. Only the original assembly (main peak) is labelled.

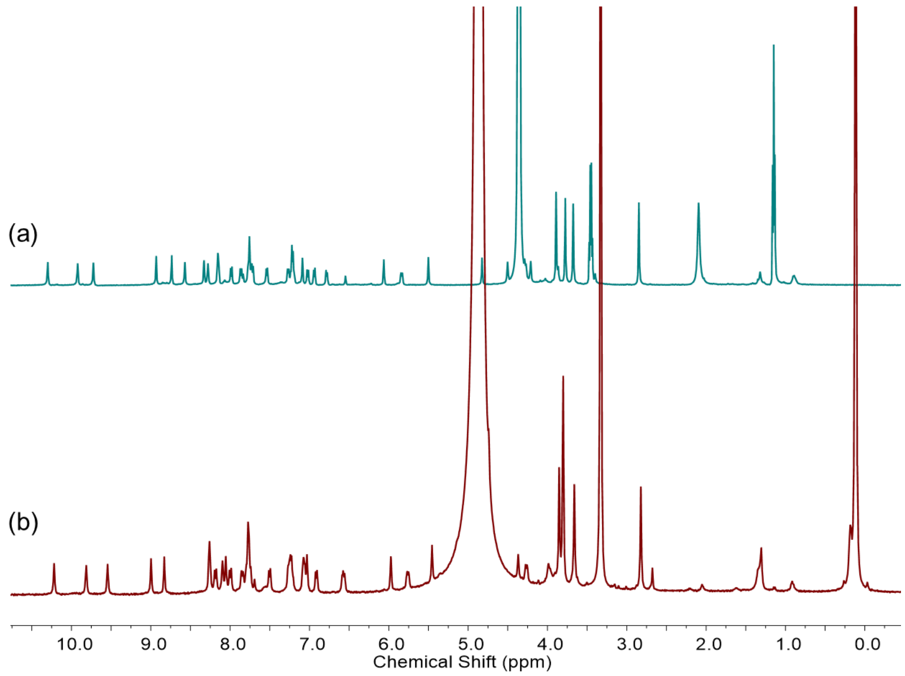

Figure S51. ^{1}H NMR spectra (400 MHz, 298 K) of **2^{OMe}** after precipitation and redissolution in (a) CD_3NO_2 and (b) CD_3OD.

8. Synthesis and characterization of $\mathbf{2}^{\mathbf{OMe}}$•OTf

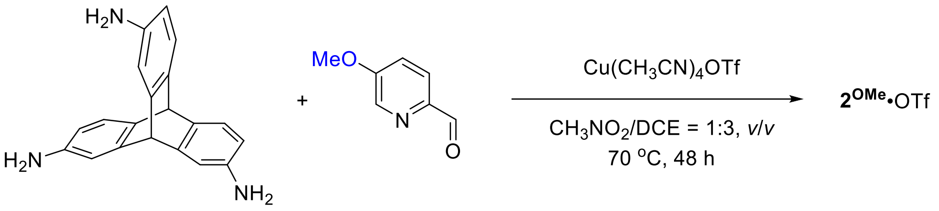

Scheme S8. Synthesis of $\mathbf{2}^{\mathbf{OMe}}$•OTf

2,7,14-triaminotriptycene (1.00 mg, 2 equiv, 3.34 μmol), 5-methoxy-2-formylpyridine (1.37 mg, 6 equiv, 10.0 μmol), tetrakis(acetonitrile)copper(I) triflate (1.89 mg, 3 equiv, 5.01 μmol), 0.20 mL of nitromethane and 0.60 mL of 1,$2^{\mathbf{OMe}}$ichloroethane were added into a small vial that was sealed in glove box. The vial was kept at 343 K for 48 h, affording a red-orange solution. The solvent was reduced by vacuum, followed by the addition of diethyl ether. Precipitates were collected by centrifugation and washed with ether three times. After drying in vacuum, $\mathbf{2}^{\mathbf{OMe}}$•OTf was obtained as a red-orange solid in approximately quantitative yield. ^{1}H NMR (400 MHz, 298 K, CD_3NO_2): δ 10.28 (s, 6H, the first imine), 9.68 (s, 6H), 9.45 (s, 6H, the second imine), 8.93 (s, 6H, the third imine), 8.73 (s, 6H, the fourth imine), 8.31 (s, 6H), 8.27 (d, J = 2.8 Hz, 6H), 8.24 (d, J = 2.8 Hz, 6H), 8.10 (d, J = 8.7 Hz, 6H), 7.96 (d, J = 8.8 Hz, 6H), 7.83 (d, J = 7.8 Hz, 6H), 7.72 (ddd, J = 8.5, 5.0, 2.8 Hz, 18H), 7.66 (s, 2H), 7.63 (dd, J = 6.0, 2.5 Hz, 14H), 7.52 (dd, J = 11.3, 8.2 Hz, 14H), 7.30 – 7.24 (m, 24H), 7.17 (d, J = 19.0 Hz, 18H), 7.02 (d, J = 7.9 Hz, 6H), 6.77 – 6.74 (m, 6H), 6.63 (s, 2H), 6.36 (s, 6H), 5.94 (dd, J = 8.8, 2.8 Hz, 6H), 5.58 (s, 2H), 5.52 (s, 6H), 5.07 (s, 6H), 4.66 (dd, J = 8.9, 2.8 Hz, 6H), 3.92 (s, 18H, the methoxyl group), 3.78 (s, 18H, the methoxyl group), 3.76 (s, 18H, the methoxyl group), 2.68 (s, 18H, the methoxyl group). ^{13}C NMR (126 MHz, 298 K, CD_3NO_2): δ 160.1, 159.6 (the first imine), 158.7, 155.5 (the second imine), 155.1 (the third imine), 154.7 (the fourth imine), 154.3, 147.8, 147.4, 146.6, 145.4, 145.3, 145.2, 145.1, 144.9, 144.5, 144.2, 143.7, 143.2, 143.0, 143.0, 142.8, 142.6, 141.6, 141.6, 141.4, 141.2, 140.0, 139.7, 139.4, 139.0, 134.4, 129.6, 129.3, 128.8, 128.2, 125.8, 125.6, 125.5, 125.4, 124.9, 124.7, 124.5, 124.2, 121.7, 121.3, 119.8, 119.6, 119.4, 119.2, 118.9, 118.8, 117.5, 116.6, 115.8, 114.2, 113.6, 67.4, 65.2, 56.4 (the methoxyl group), 55.9 (the methoxyl group), 55.6 (the methoxyl group), 55.5 (the methoxyl group), 53.2, 52.0, 51.9. ^{19}F NMR (376 MHz, 298 K, CD_3NO_2): δ –74.44 (encapsulated OTf^{-}), –79.75 (free OTf^{-}).

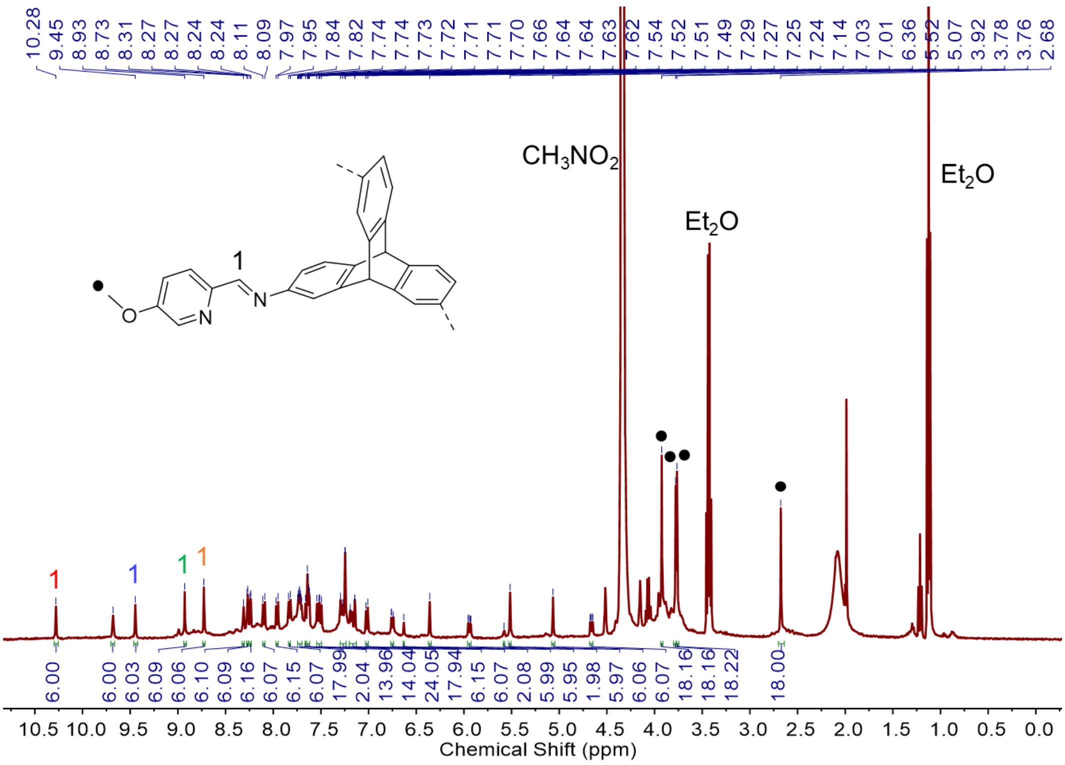

Figure S52. ^{1}H NMR spectrum (400 MHz, 298 K, CD_3NO_2) of **2[OMe]**•OTf.

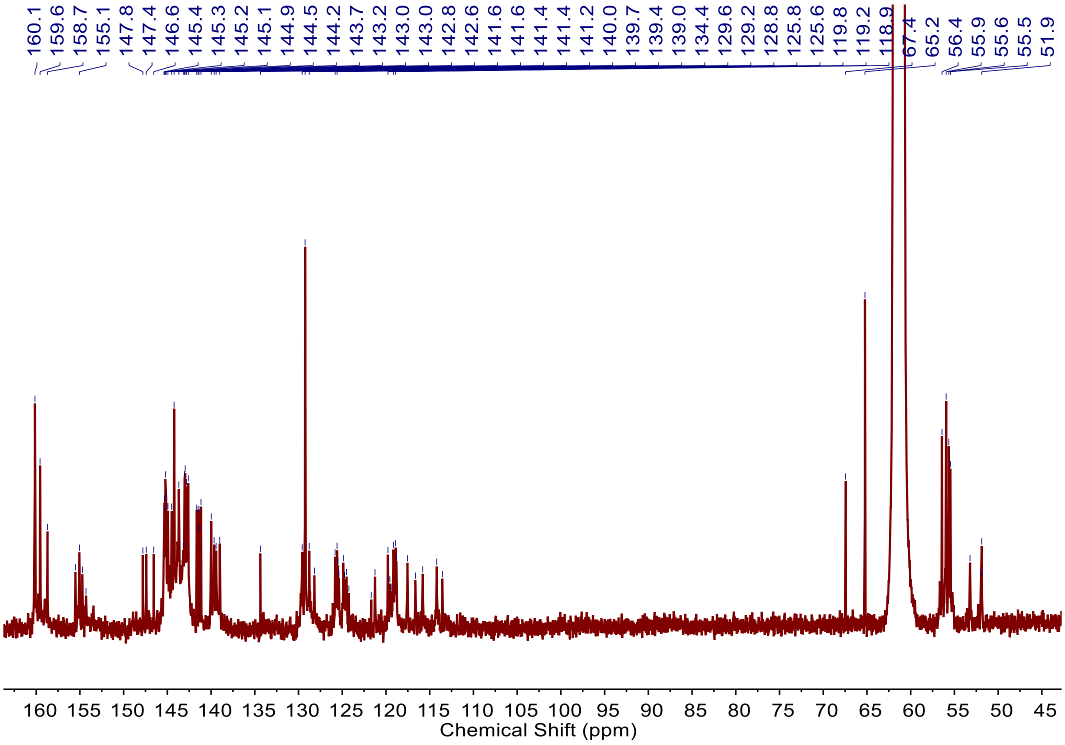

Figure S53. ^{13}C NMR spectrum (126 MHz, 298 K, CD_3NO_2) of **2[OMe]**•OTf.

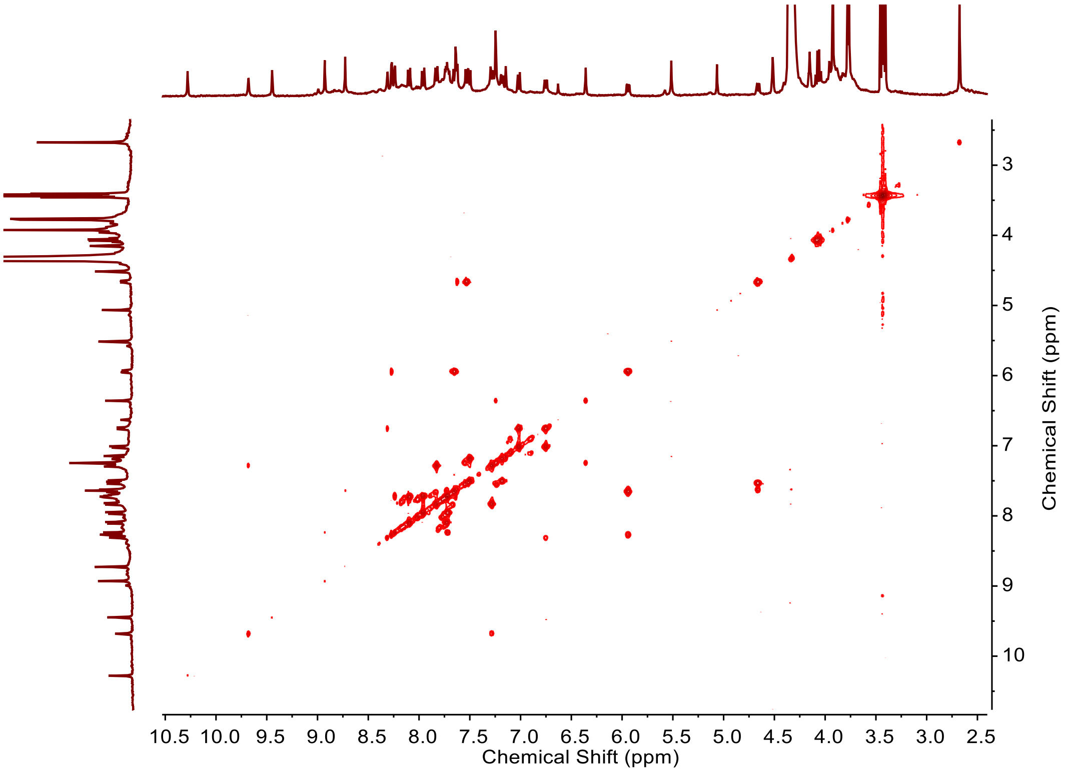

Figure S54. ^{1}H-^{1}H COSY spectrum (400 MHz, 298 K, CD_3NO_2) of $\mathbf{2}^{\mathbf{OMe}}$•OTf.

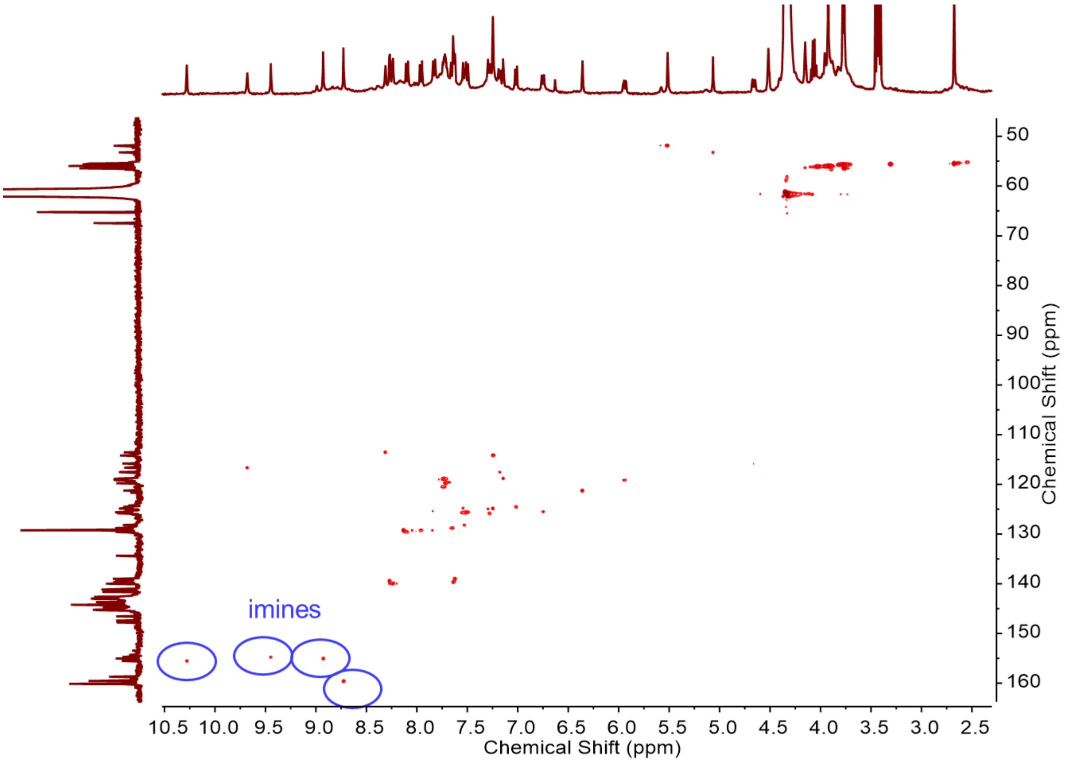

Figure S55. ^{1}H-^{13}C HSQC spectrum (400 MHz, 298 K, CD_3NO_2) of $\mathbf{2}^{\mathbf{OMe}}$•OTf.

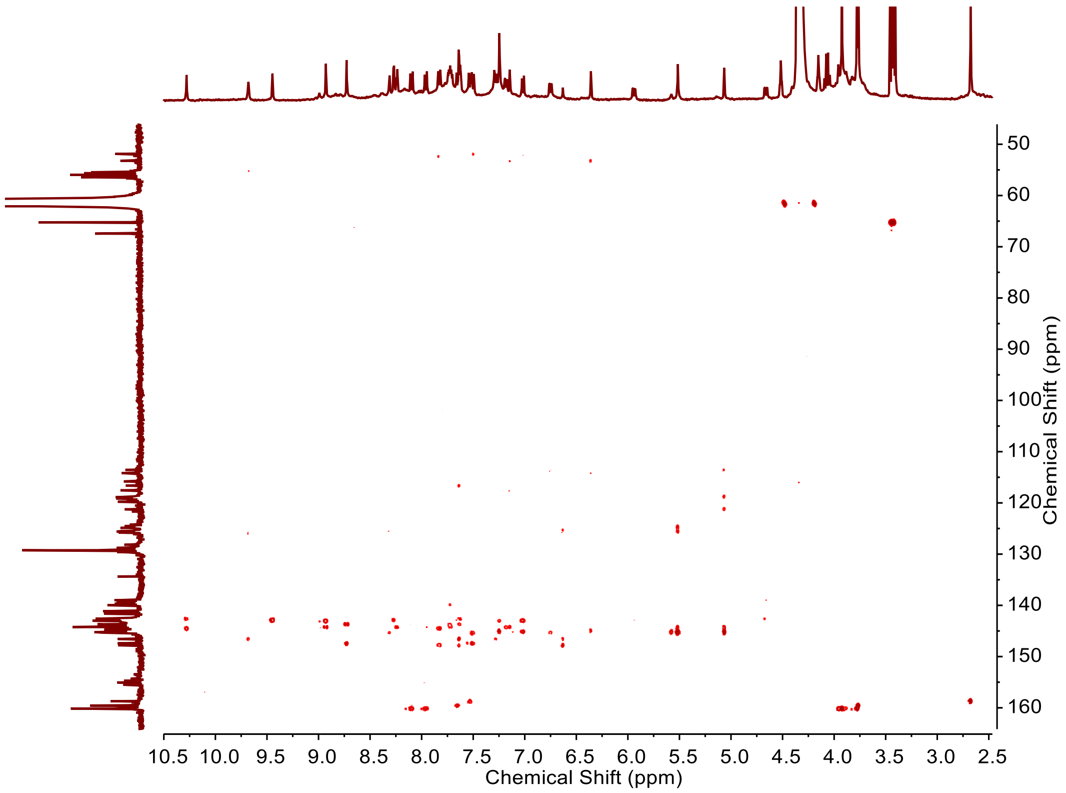

Figure S56. ^{1}H-^{13}C HMBC spectrum (400 MHz, 298 K, CD_3NO_2) of $\mathbf{2}^{\mathbf{OMe}}$•OTf.

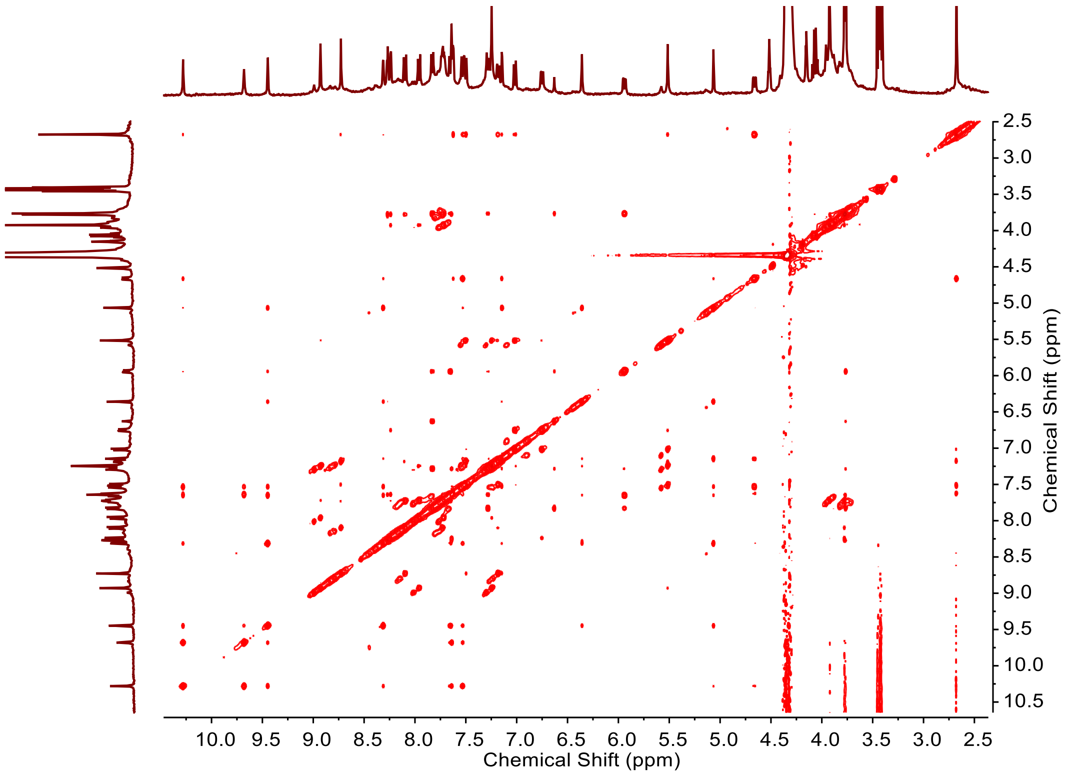

Figure S57. ^{1}H-^{1}H NOESY spectrum (400 MHz, 298 K, CD_3NO_2) of $\mathbf{2}^{\mathbf{OMe}}$•OTf.

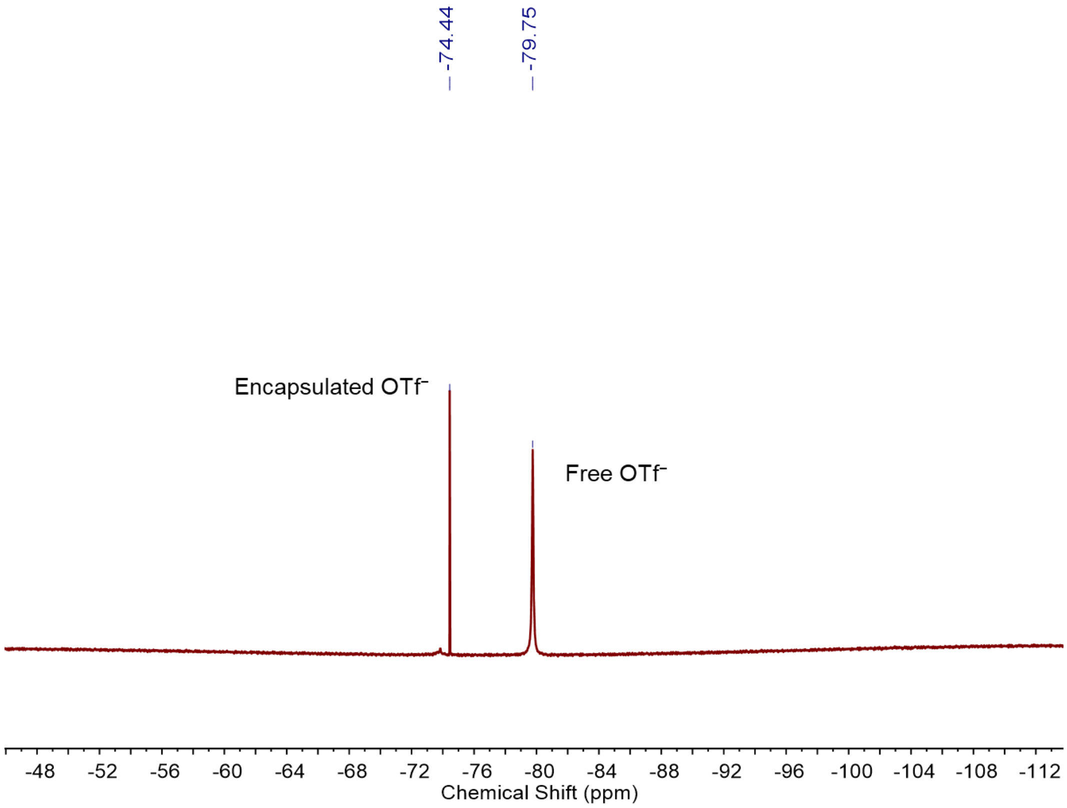

Figure S58. ^{19}F NMR spectrum (376 MHz, 298 K, CD_3NO_2) of $\mathbf{2}^{\mathbf{OMe}}$•OTf.

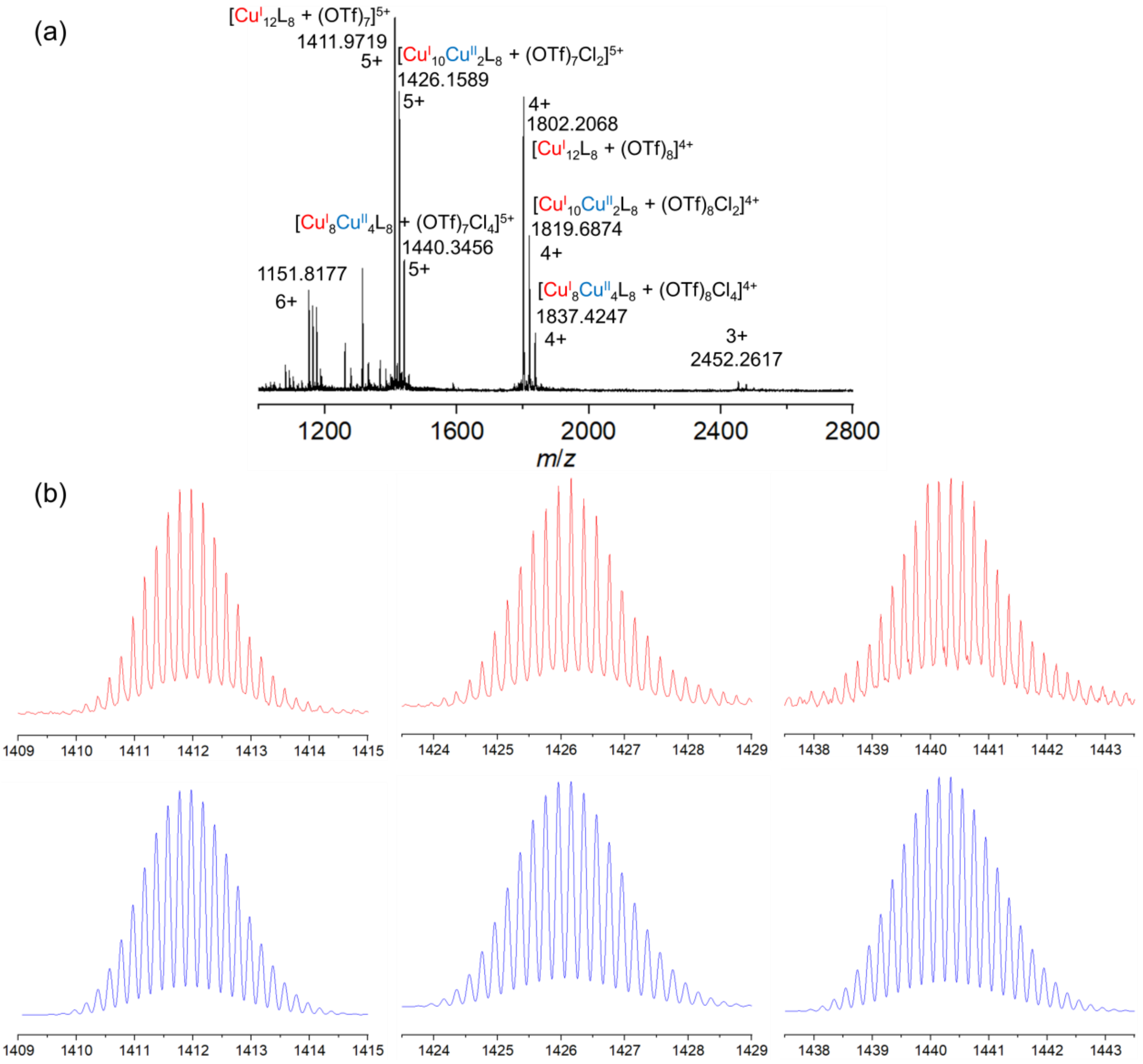

Figure S59. High-resolution ESI-mass spectrum of $\mathbf{2^{OMe}}$•OTf: (a) Full spectrum and (b) selected cations ($[Cu^{I}_{12}L_8 + (OTf)_7]^{5+}$, $[Cu^{I}_{10}Cu^{II}_{2}L_8 + (OTf)_7Cl_2]^{5+}$, and $[Cu^{I}_{8}Cu^{II}_{4}L_8 + (OTf)_7Cl_4]^{5+}$, from left to right) with experimental (red) and calculated (blue) isotopic distributions. In the full spectrum, there are two extra peaks near each main peak (6+, 5+, 4+, and 3+) of the assembly, which are ascribed to the addition of two Cl^{-} and four Cl^{-}. We infer that this assembly is partially oxidised in the mass spectrometer, producing Cu^{II} species with chloride as counteranions. Peaks in 5+ and 4+ regions are clearly labelled. Many attempts with different ionisation conditions and solvents have been done, but they all give the same result.

9. Solvent effect on the self-assembly

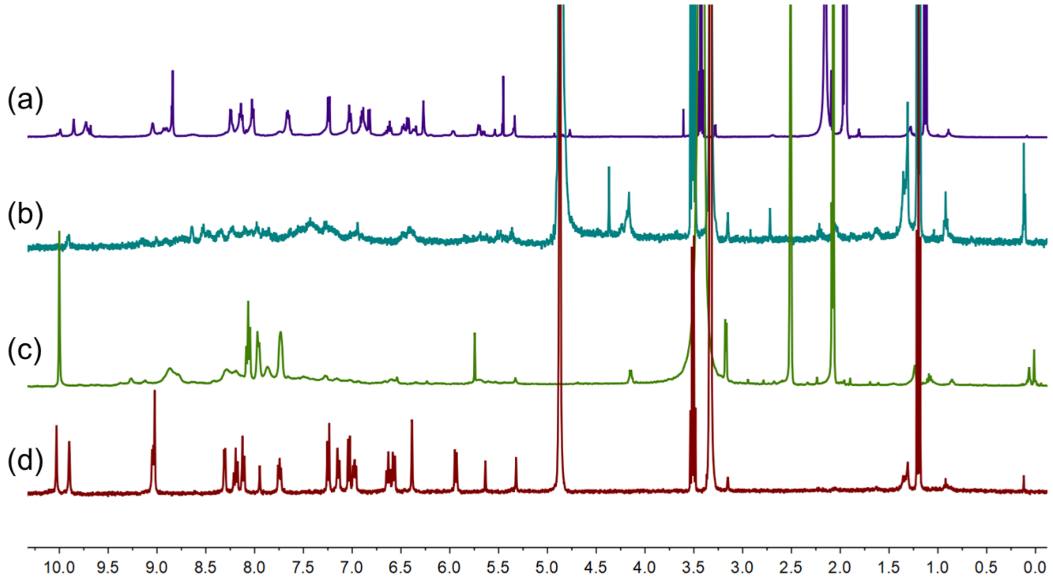

Figure S60. ^{1}H NMR spectra (400 MHz, 298 K) of the reactions of the triptycene ligand, 2-formylpyridine and tetrakis(acetonitrile)copper(I) tetrafluoroborate in different solvent: (a) CD_3CN, (b) CD_3NO_2, (c) DMSO-d_6 and (d) CD_3OD. 1.00 mg of the triptycene ligand and 0.70 mL of corresponding solvent were used in each reaction.

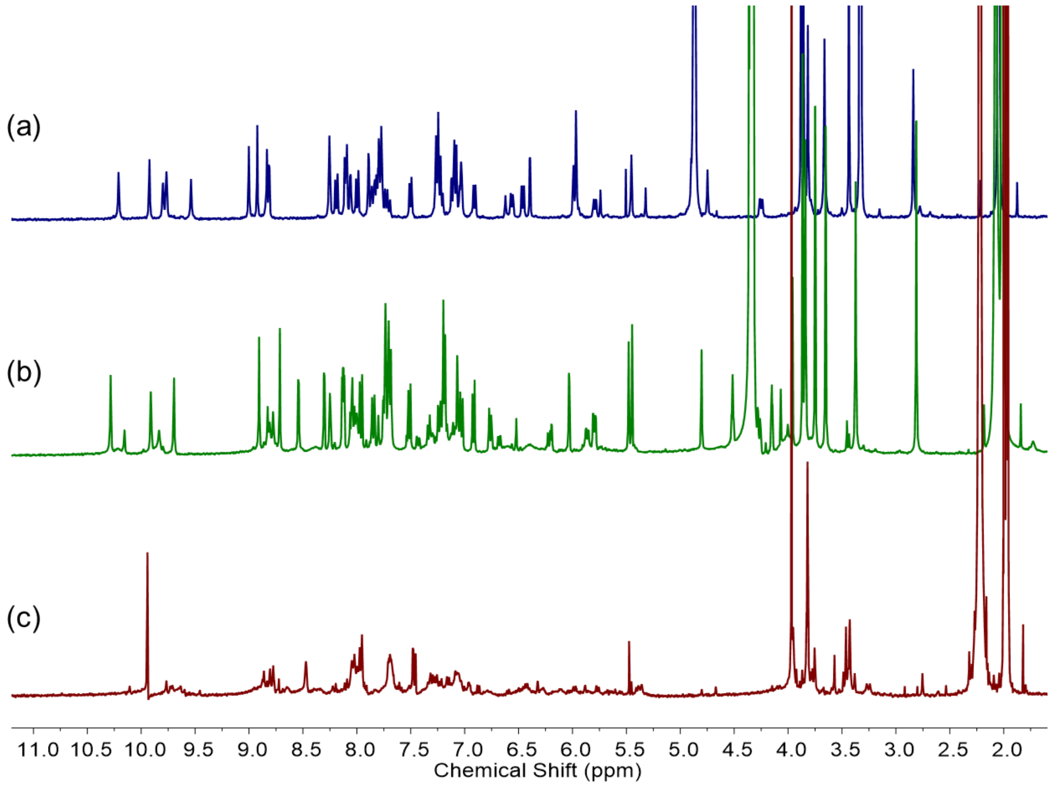

Figure S61. ^{1}H NMR spectra (400 MHz, 298 K) of the crude reaction mixtures of the triptycene subcomponent, 5-methoxy-2-formylpyridine and tetrakis(acetonitrile)copper(I) tetrafluoroborate in different solvents: (a) CD_3OD, (b) CD_3NO_2 and (c) CD_3CN.

10. BF_4^- binding of **1$^{H/F}$**•OTf and **2^{OMe}**•OTf

In the crystal structure of **1^{F}**•BF_4, four BF_4^- anions were encapsulated inside the assembly, distributing in a tetrahedral mode. The cavity of this assembly was calculated by MoloVol,[2] after removing the bound anions. The shape of the cavity was found to be a tetrahedron-like void, with a total volume of 234.49 Å^3 (including the centre). Figure S60a shows an overlap between the cavity (green mesh) and bound BF_4^-. The BF_4^- anion is bound at four corners of the cavity.

The cavities of assemblies prepared by either 2-formylpyridine or 5-fluoro-2-formylpyridine are believed to be identical due to the same cage framework. This is also supported by the ^{19}F NMR spectrum of **1$^{H/F}$**•BF_4, in which bound BF_4^- is observed.

The assembly **1^{H}**•OTf, however, exhibits a very weak binding of OTf$^-$ in methanol, as evidenced by its ^{19}F NMR spectrum, shown below and in Figure S18. This can be explained by the limited cavity size at each corner. We inferred that this assembly could bind four BF_4^- anions as the cavity was empty.

^{1}H and ^{19}F NMR titration was carried out subsequently. Tetrabutylammonium tetrafluoroborate (TBABF$_4$) was added into a solution of **1^{H}**•OTf in methanol, and the ^{1}H NMR spectrum was collected after 5 min of addition. Obtained spectra are shown below. The ratios of TBABF$_4$ added are also displayed. In the ^{1}H NMR spectra (Figure S60b), the signals after the addition of 14.0 equiv of TBABF$_4$ are identical with those of **1^{H}**•BF_4 at the bottom, indicating the encapsulation is complete. Multiple sets of peaks of intermediates during the titration also imply a slow-exchange behaviour of BF_4^- binding. The messy spectra during titration are explained by different intermediates (host-guest complexes), which are discussed below.

In the ^{19}F NMR spectra (Figure S60c and d), details of the binding behaviour during titration are revealed by the signals of bound anions. Initially, the spectrum of **1^{H}**•OTf infers that the assembly barely binds OTf$^-$, as the signal of bound OTf$^-$ is negligible. This is because the corners of the cavity are not large enough to bind OTf$^-$ at this stage. However, after the addition of BF_4^- (less than four equiv), signals of encapsulated BF_4^- and OTf$^-$ simultaneously increase, implying that the binding of BF_4^- improves the affinity to OTf$^-$. Thus, a cooperative binding mode is revealed. We speculate that after inclusions of one or two or three BF_4^- anions, the corners of the cavity slightly expand, which fit the size of OTf$^-$ and thereby the enhanced binding of OTf$^-$ appears. In addition, after adding largely excessive BF_4^- (more than six equiv), the signals of bound OTf$^-$ gradually disappear, illustrating a competitive binding mode. This can be explained by stronger binding affinity to BF_4^-. Consequently, by titration of BF_4^-, an interesting binding behaviour, the cooperative binding followed by competitive binding, is discovered.

Besides, intermediates of binding are supported by more than one set of peaks of OTf$^-$ and BF_4^-. The generation of intermediates is reasonable, because the cavity of the assembly can accommodate four

anions that can be either BF_4^- or OTf^- and the binding ratio of them also varies. Quantitive analysis of this binding process was not feasible because of the co-existence of many complexes with slow exchange dynamics.

We have also tried the single-crystal-to-single-crystal transformation of $\mathbf{1^F}\bullet BF_4$ in the presence of TBAOTf, but without success. Immersing a crystal of $\mathbf{1^F}\bullet BF_4$ in a methanol or acetonitrile solution of TBAOTf dissolved the crystal. Soaking single crystals in other solvents that did not dissolve $\mathbf{1^F}\bullet BF_4$, such as diethyl ether and chloroform, containing TBAOTf, resulted in crystals that were unstable after being taken out of the solvent. None of these experiments produced single crystals capable of X-ray diffraction. We also attempted to grow single crystals of $\mathbf{1^F}$ in the presence of a mixture of BF_4^- and TfO^- anions, but we only obtained crystals of pure $\mathbf{1^F}\bullet BF_4$.

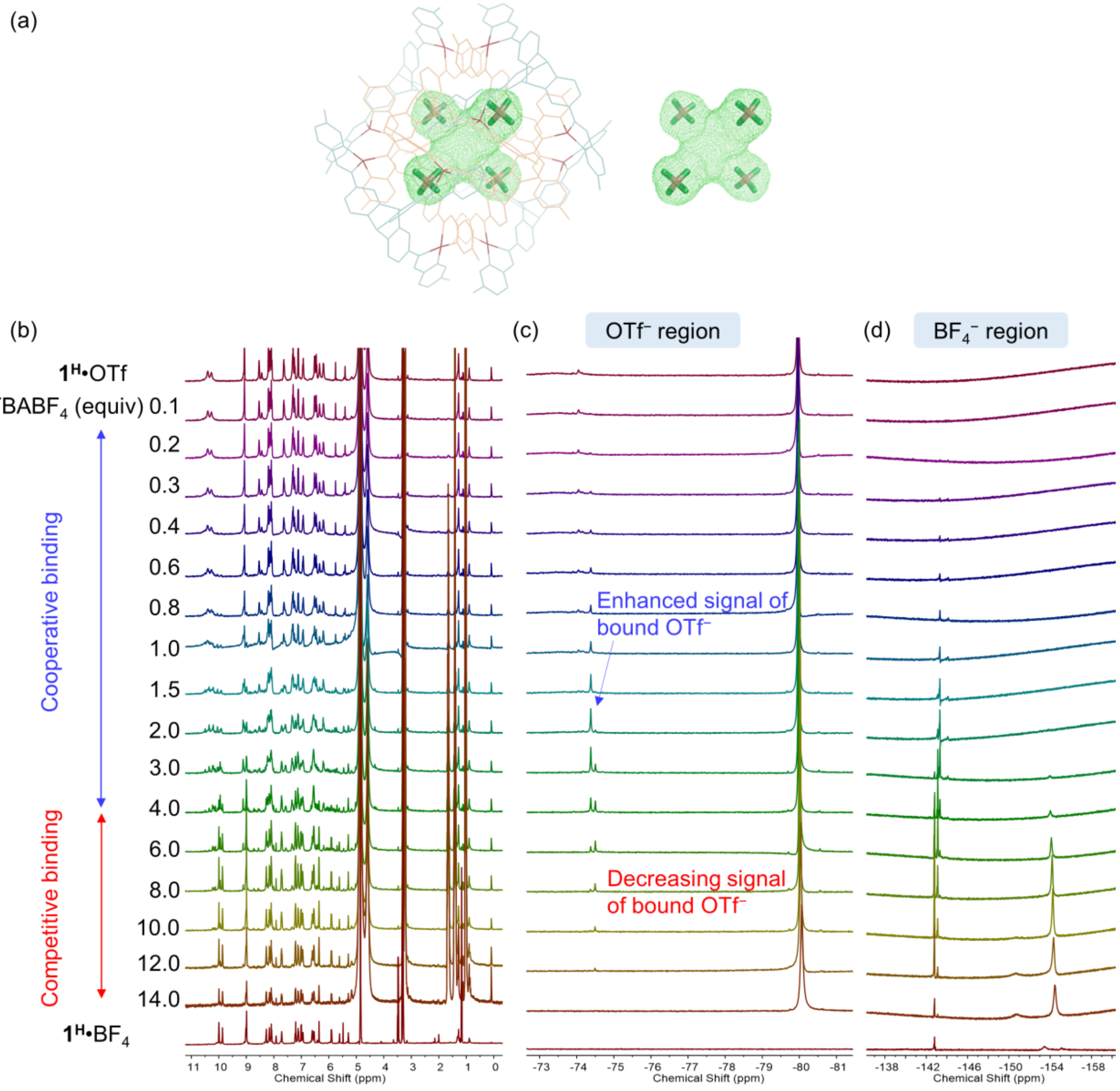

Figure S62. (a) The cavity mapping of $\mathbf{1^H}\bullet BF_4$ (left) and the overlap between the cavity and four bound BF_4^- anions. (b) ^1H NMR spectra and (c,d) ^{19}F NMR spectra of the titration of $TBABF_4$ to the methanol

solution of **1^H^**•OTf. Identifications of peaks in the enlarged spectra are shown in Figure 3 in the manuscript.

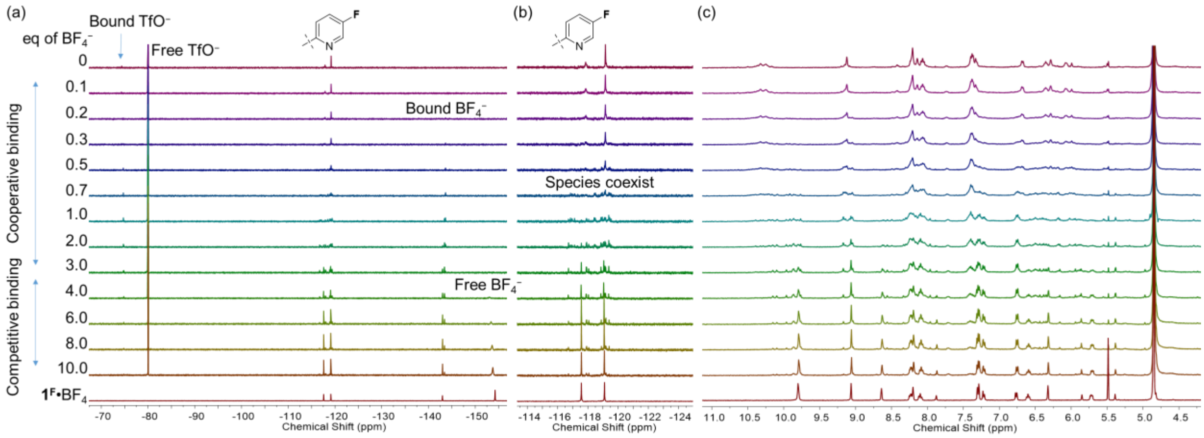

Figure S63. (a) and (b) ^{19}F ^{1}H NMR spectra and (c) ^{1}H NMR spectra of the titration of $TBABF_4$ into a methanol solution of $\mathbf{1^F}$•OTf. The enlargement in (b) indicates multiple sets of peaks belonging to the fluorine substituents on the pyridine rings.

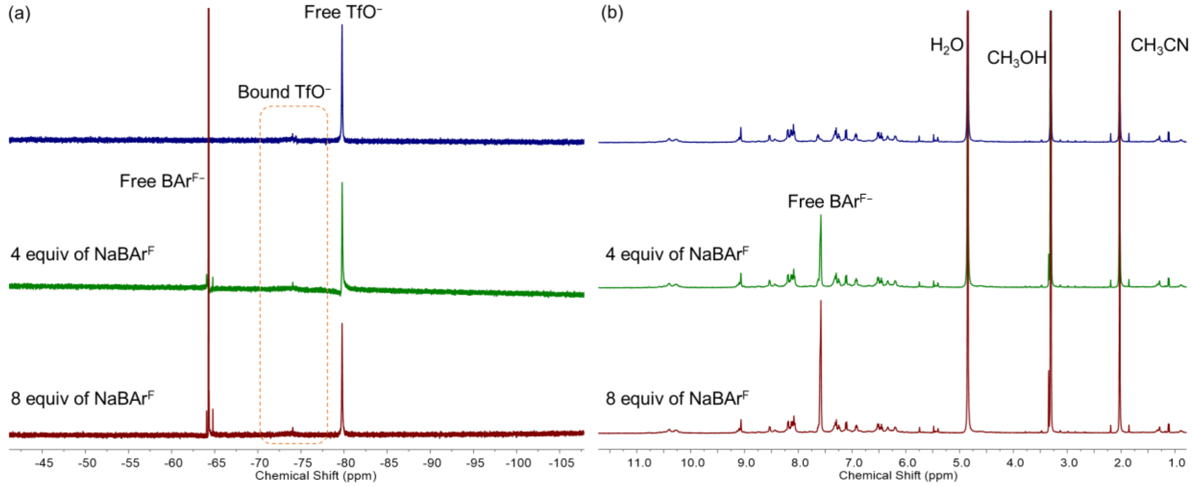

Figure S64. (a) ^{19}F NMR spectra and (b) ^{1}H NMR spectra of the titration of $NaBAr^F$ to the methanol solution of $\mathbf{1^H}$•OTf.

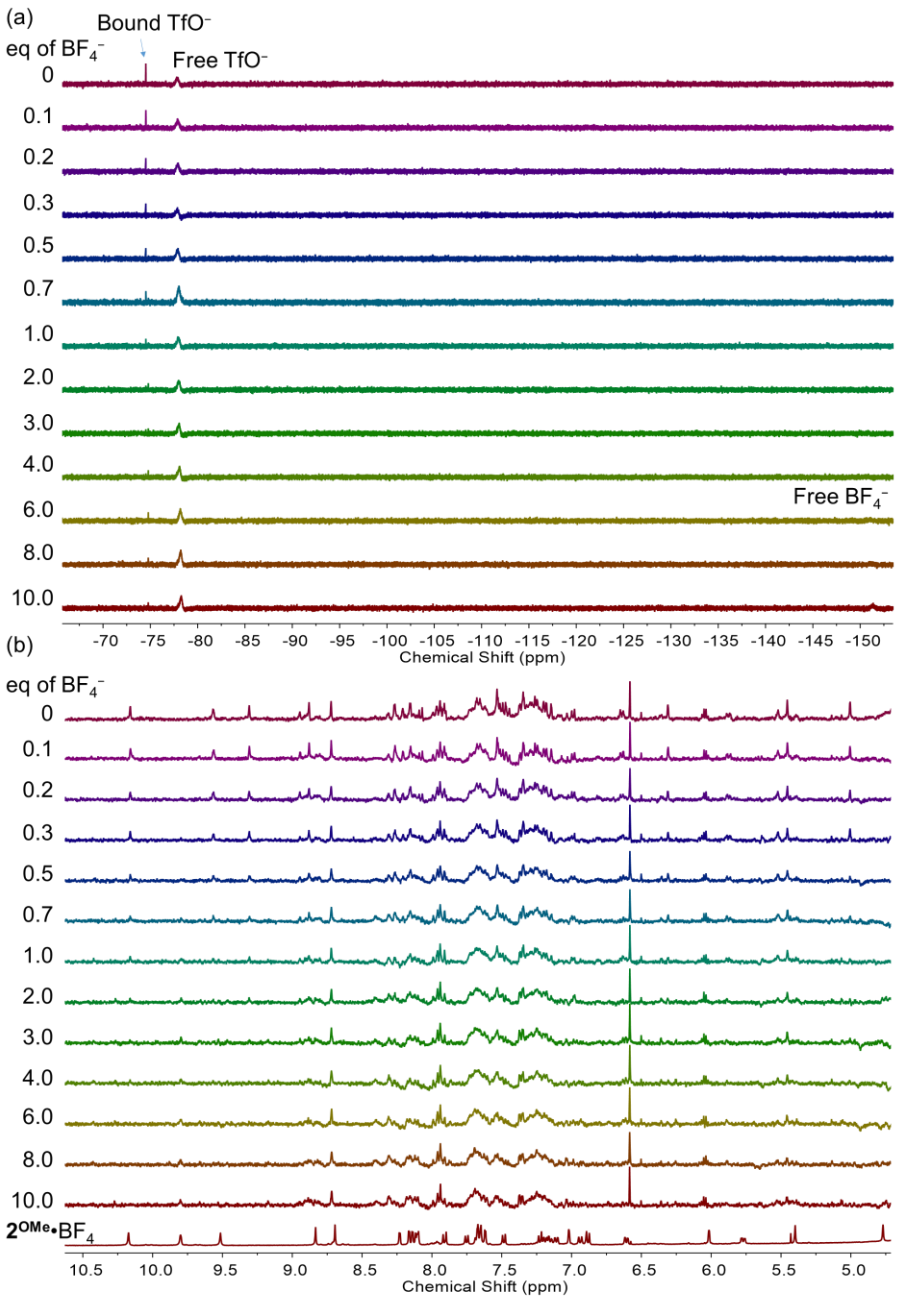

Figure S65. (a) ^{19}F NMR spectra (376 MHz, 298 K) and (b) ^{1}H NMR spectra (400 MHz, 298 K) of **2**$^{\mathbf{OMe}}$•OTf during the addition of $TBABF_4$ showing gradual disassembly. **2**$^{\mathbf{OMe}}$•BF_4 (bottom spectrum) was not formed during the titration. No bound BF_4^- was observed. The solvent is CD_3NO_2 and non-deuterated dichloroethane (1:3, *v*/*v*).

11. Effects of solvents and electron density of subcomponents

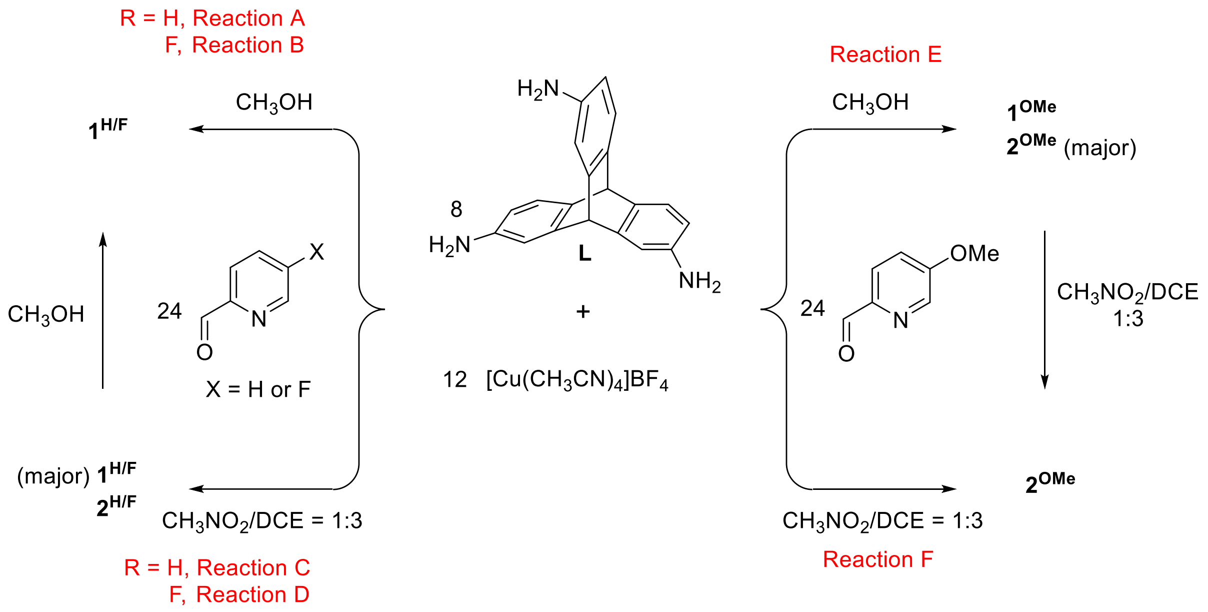

Scheme S7. Self-assembly under different conditions.

Six reactions were carried out to determine the effects of solvent and electron density of subcomponents. In each reaction, the scale was the same, and 1.00 mg of 2,7,14-triaminotriptycene as well as 0.70 mL of solvents were used. Integrations on ^{1}H NMR spectra of each reaction gave the ratios of two copper(I) assemblies. The ^{1}H NMR spectra of Reactions C, D, and E are shown below. Reactions A, B and F afforded the pure assembly, thus their ^{1}H NMR spectra can be found in the above Sections of synthesis. The results are shown in Figure 3a in the manuscript.

The resulting solution of either Reaction C or Reaction D was dried in vacuum, affording solids of two assemblies. To these solid, 0.70 mL of methanol was added. The solution was kept at 70 °C overnight. The ^{1}H NMR spectrum indicated that only **1**$^{\mathbf{H}}$ or **1**$^{\mathbf{F}}$ was present. The other framework transformed to **1**. Reaction H was carried out in the same way, and only **2**$^{\mathbf{OMe}}$ was observed.

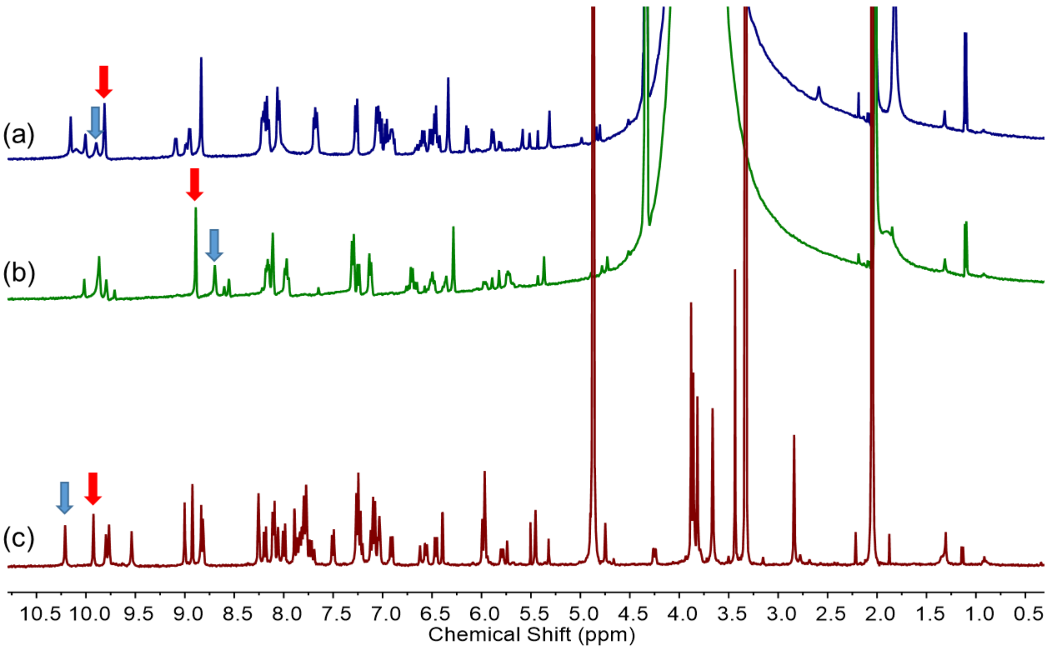

Figure S66. ^{1}H NMR spectra (400 MHz, 298 K) of (a) Reaction C in CD_3NO_2/DCE, (b) Reaction D in CD_3NO_2/DCE and (c) Reaction E in CD_3OD. The huge peak at 3-4.5 ppm is from non-deuterated DCE. The peaks for integration are marked with arrows (framework **1**, red; framework **2**, blue).

12. Photoluminescence studies of **1** and **2**

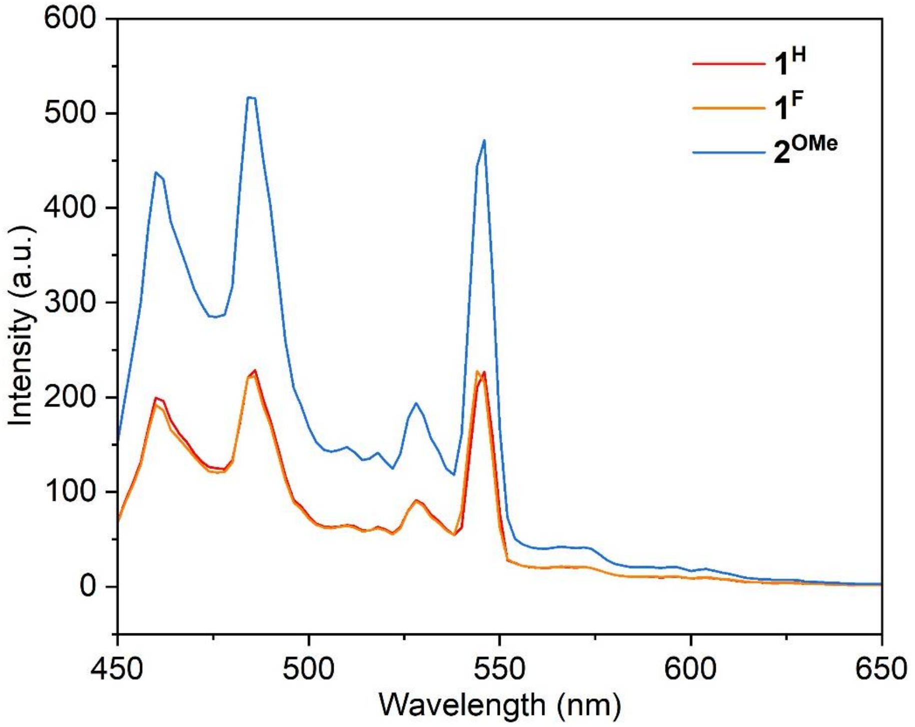

Figure S67. Photoluminescence spectra of $\mathbf{1^H}$, $\mathbf{1^F}$ and $\mathbf{2^{OMe}}$ in methanol at a concentration of 1×10^{-5} M. The excitation wavelength is 400 nm. Excitation at 450 and 500 nm did not afford strong emission.

13. X-ray crystallography

1^F•BF_4

Data were collected at Beamline I19 of Diamond Light Source employing silicon double crystal monochromated synchrotron radiation (0.6889 Å) with ω and ψ scans at 100(2) K.[3] Data integration and reduction were undertaken with Xia2.[4] Subsequent computations were carried out using the WinGX-32 graphical user interface.[5] A multi-scan empirical absorption correction using spherical harmonics was applied to the data using DIALS.[4b] The structure was solved by intrinsic phasing using SHELXT[6] then refined and extended with SHELXL.[7] Carbon-bound hydrogen atoms were included in idealised positions and refined using a riding model. Disorder was modelled using standard crystallographic methods including constraints and restraints where necessary.

The crystals of **1^F**•BF_4 were grown by diffusion of diethyl ether into a methanol solution of the complex. The crystals employed immediately lost solvent after removal from the mother liquor. However rapid handling prior to flash cooling in liquid nitrogen and the use of synchrotron radiation enabled the collection of high resolution data. The asymmetric unit was found to contain one third of a $Cu_{12}L_8$ assembly as well as associated counterions and solvent molecules. Thermal parameter restraints (SIMU, RIGU) were applied to all atoms except for copper. The Cu-N bond lengths and angles within the structure are fully consistent with the presence of copper(I), as also reflected in the sharp, diamagnetic NMR spectra of this complex.

The anions within the structure also show evidence of substantial disorder. Four tetrafluoroborate anions (including one of the encapsulated anions) were modelled as disordered over two locations and the occupancies of all located anions were freely refined. The tetrafluoroborate anions were restrained to be approximately tetrahedral and lower occupancy anions were refined with isotropic thermal parameters. Some additional minor occupancy positions of the anions could not be located in the electron density map and were not included in the model resulting in a discrepancy of ca. 2 counterions per $Cu_{12}L_8$ assembly (or 0.7 per asymmetric unit).

Consequently the SQUEEZE[8] function of PLATON[9] was employed to remove the contribution of the electron density associated with the remaining anions and further highly disordered solvent, which gave a potential solvent accessible void of 15251 Å^3 per unit cell (a total of approximately 4252 electrons). Diffuse solvent molecules could not be assigned to methanol or diethyl ether and were therefore not included in the formula. Consequently, the molecular weight and density given above are underestimated.

Crystallographic data have been deposited with the CCDC (2224726).

Formula $C_{304}H_{184}B_{12}Cu_{12}F_{72}N_{48}$, *M* 6769.18, Trigonal, space group R-3 (#148), *a* 24.68720(10), *b* 24.68720(10), *c* 102.5904(4) Å, γ 120°, *V* 54147.8(5) Å^3, D_c 1.246 g cm^{-3}, *Z* 6, crystal size 0.040 by 0.040 by 0.030 mm, colour brown, habit block, temperature 100(2) Kelvin, λ(Synchrotron) 0.6889 Å, μ(Synchrotron) 0.717 mm^{-1}, *T*(Analytical)$_{min,max}$ 0.855375884930681, 1.0, $2\theta_{max}$ 64.00, *hkl* range -28 28, -28 37, -156 156, *N* 174802, N_{ind} 45129(R_{merge} 0.0448), N_{obs} 30064(I > 2σ(I)), N_{var} 1423, residuals* $R1(F)$ 0.0663, $wR2(F^2)$ 0.2309, GoF(all) 1.076, $\Delta\rho_{min,max}$ -0.654, 1.132 e$^-$ Å^{-3}.

*$R1 = \Sigma||F_o| - |F_c||/\Sigma|F_o|$ for $F_o > 2\sigma(F_o)$; $wR2 = (\Sigma w(F_o^2 - F_c^2)^2/\Sigma(wF_c^2)^2)^{1/2}$ all reflections

$w=1/[\sigma^2(F_o^2)+(0.1287P)^2+41.4442P]$ where $P=(F_o^2+2F_c^2)/3$

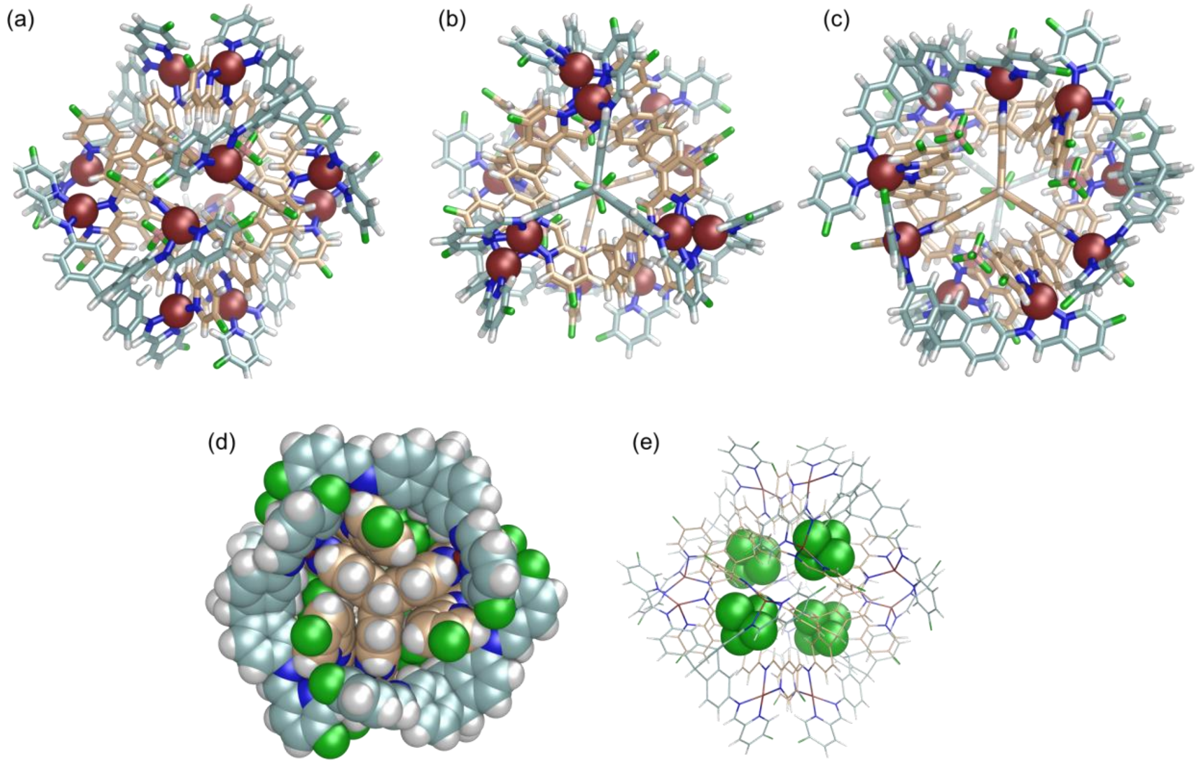

Figure S68. Views of $\mathbf{1^F}$•BF_4 through (a) the C_2 axis, (b) one C_3 axis and (c) the other C_3 axis in stick representations and (d) through a C_3 axis in space-filling representations, highlighting the *T* symmetry of the assembly. (e) Illustrates of four bound BF_4^- anions that are in space-filling representations. Free counteranions, solvents and disorder are omitted for clarity. Hydrogen atoms are white, fluoride atoms are green, nitrogen atoms are blue, and copper atoms are ruby. The outer ligands are coloured in pale cyan and the inner ligands are coloured in wheat.

1^F^•OTf

Data were with collected using a Bruker D8 VENTURE equipped with high-brilliance IμS Cu-Kα radiation (1.54178 Å), with ω and ψ scans at 180(2) K. Data integration and reduction were undertaken with Bruker APEX 4 and XPREP. Subsequent computations were carried out using the WinGX-32 graphical user interface. A multi-scan empirical absorption correction was applied to the data using SADABS. The structure was solved by intrinsic phasing using SHELXT[6] then refined and extended with SHELXL.[7] Disorder was modelled using standard crystallographic methods including constraints and restraints where necessary.

The crystals of $\mathbf{1^F}$•OTf were grown by diffusion of diethyl ether into a methanol solution of the complex. The crystals employed immediately lost solvent after removal from the mother liquor but rapid handling prior to flash cooling in the cryostream enabled data to be collected to ca. 0.85 Å resolution. The asymmetric unit was found to contain one third of a $Cu_{12}L_8$ assembly as well as associated counterions and solvent molecules. Bond lengths and angles within pairs of chemically identical organic ligands were restrained to be similar to each other. Thermal parameter restraints (SIMU, RIGU) were applied to all atoms except for copper. The Cu-N bond lengths and angles within the structure are fully consistent with the presence of copper(I), as also reflected in the sharp, diamagnetic NMR spectra of this complex.

The anions within the structure show evidence of substantial disorder. One of the encapsulated triflate anions (also located on a 3-fold axis) was modelled as disordered end-for-end with approximately equal occupancies for the two orientations. Two further triflate anions were modelled as disordered over two or three locations and the occupancies of all located anions were freely refined. Substantial bond length and angle restraints were applied to the disordered triflate anions and a low occupancy anion was refined with isotropic thermal parameters. Some additional minor occupancy positions of the anions could not be located in the electron density map and were not included in the model resulting in a discrepancy of ca. 1.3 counterions per $Cu_{12}L_8$ assembly (or 0.43 per asymmetric unit). Hydrogen atoms could not be located for the water and methanol solvent molecules and were therefore not included in the model.

Consequently, the SQUEEZE[8] function of PLATON[9] was employed to remove the contribution of the electron density associated with the remaining anions and further highly disordered solvent, which gave a potential solvent accessible void of 10162 Å^3 per unit cell (a total of approximately 3558 electrons). Diffuse solvent molecules could not be assigned to methanol or diethyl ether and were therefore not included in the formula. Consequently, the molecular weight and density given above are underestimated.

CheckCIF gives one A and four B level alerts, all resulting from the solvent molecules for which hydrogen atoms were not modelled (short contacts from missing H-bonds, apparent singly bonded carbon atoms).

The cavity of this cage increases to 297 Å^3 (probe radius 1.6 Å), calculated by MoloVol.[2]

Crystallographic data have been deposited with the CCDC (2300612). Formula $C_{326.50}H_{229}Cu_{12}F_{60}N_{48}O_{48}S_{12}$, *M* 7879.76, Trigonal, space group R-3 (#148), *a* 25.3300(4), *b* 25.3300(4), *c* 105.864(3) Å, γ 120°, *V* 58823(3) Å^3, D_c 1.335 g cm^{-3}, *Z* 6, crystal size 0.170 by 0.160 by 0.050 mm, colour brown, habit block, temperature 180(2) Kelvin, λ(CuKα) 1.54178 Å, μ(CuKα) 2.117 mm^{-1}, T(SADABS)$_{min,max}$ 0.6080, 0.7526, 2θ_{max} 130.46, *hkl* range -29 29, -29 25, -124 48, *N* 111769, N_{ind} 22314(R_{merge} 0.0832), N_{obs} 13574(I > 2σ(I)), N_{var} 1750, residuals* $R1(F)$ 0.1053, $wR2(F^2)$ 0.3452, GoF(all) 1.096, $\Delta\rho_{min,max}$ -0.644, 1.240 e$^-$ Å^{-3}.

*$R1 = \Sigma||F_o| - |F_c||/\Sigma|F_o|$ for $F_o > 2\sigma(F_o)$; $wR2 = (\Sigma w(F_o^2 - F_c^2)^2/\Sigma(wF_c^2)^2)^{1/2}$ all reflections

$w=1/[\sigma^2(F_o^2)+(0.2000P)^2+300.0000P]$ where $P=(F_o^2+2F_c^2)/3$

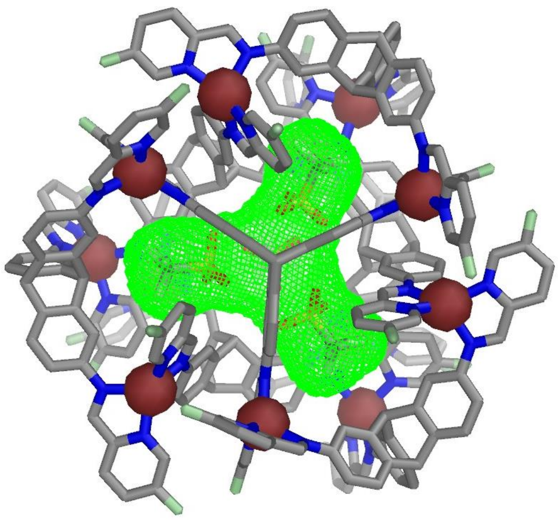

Figure S69. Crystal structure of **1^F**•OTf, viewing along C_3 axis. The cavity (297 Å^3), determined by MoloVol with a 1.6 Å probe, is shown in green mesh.

2^{OMe}*•*BF$_4$

The crystals of **2^{OMe}**•BF_4 were grown by diffusion of di-isopropyl ether into a methanol solution of the complex. The crystal was mounted on the diffractometer at the synchrotron Elettra, Trieste (Italy), beamline XRD1, using the robot present at the facility. Temperature was kept at 100 K by a stream of nitrogen on the crystal. Diffraction data were collected by the rotating crystal method using synchrotron radiation, wavelength 0.70 Å, rotation interval 1°/image, crystal-to-detector distance of 85 mm. A total of 360 images were collected. Reflections were indexed and integrated using the XDS package[10], space

group P21 was determined using POINTLESS[11] and the resulting data set was scaled using SCALA[12] of the CCP4 package[13]. Phase information were obtained by direct methods using the software SHELXT[7]. Refinements cycles were conducted with SHELXL-14[7] Carbon-bound hydrogen atoms were included in idealised positions and refined using a riding model. Disorder was modelled using standard crystallographic methods including constraints and restraints where necessary.

The crystals employed immediately lost solvent after removal from the mother liquor. However rapid handling prior to flash cooling in liquid nitrogen and the use of synchrotron radiation enabled the collection of high resolution data. The asymmetric unit was found to contain the whole $Cu_{12}L_8$ assembly as well as associated counterions and solvent molecules. Thermal parameter restraints (SIMU, RIGU) were applied to all atoms except for copper.

GRADE program[144] was employed using the GRADE Web Server[15] to generate a full set of bond distance and angle restraints (DFIX, DANG, FLAT) for the organic parts of the structure. Thermal parameter restraints (SIMU, RIGU) were applied to all atoms except for copper to facilitate a stable anisotropic refinement. Even with these restraints some thermal parameters remain larger than ideal as a consequence of the high level of thermal motion throughout the structure.

The anions within the structure also show evidence of substantial disorder. Two encapsuled tetrafluoroborate anions were modelled as disordered over two locations and the occupancies of all located anions were freely refined. The tetrafluoroborate anions were restrained to be approximately tetrahedral and lower occupancy anions were refined with isotropic thermal parameters. Some additional solvent molecules could not be located in the electron density map and were not included in the model.

Therefore, the SQUEEZE[8] function of PLATON[9] was employed to remove the contribution of the electron density associated with highly disordered solvent, which gave a potential solvent accessible void of 11475 Å^3 per unit cell (a total of approximately 3156 electrons). Diffuse solvent molecules could not be assigned to methanol or ethyl ether and were therefore not included in the formula. Consequently, the molecular weight and density given above are underestimated.

CheckCIF gives four B level alerts. These alerts result from the flexibility of the structure, high fraction of disordered solvent/ions and weak crystal quality, the high level of thermal motion around the BF_4 anions.

Crystallographic data have been deposited with the CCDC (2233061).

Formula $C_{329}H_{260}B_{12}Cu_{12}F_{48}N_{48}O_{25}$, Formula weight 7090.03, Temperature 100(2), Crystal system monoclinic, Space group (number) C2/c (15), a 78.439(16), b 19.647(4) c 46.219(9) α 90, β 93.78(3), γ 90, Volume 71073(25), Z 8, ρ_{calc} 1.325, μ 0.793, F(000) 28880, Crystal size [mm^3] 0.100×0.050×0.020,

Crystal colour black, Crystal shape block, Radiation Synchrotron (λ = 0.700 Å), 2Θ range [°] 1.74 to 51.88 (0.80 Å), Index ranges -97 ⩽ h ⩽ 97, -24 ⩽ k ⩽ 24, -57 ⩽ l ⩽ 57, Reflections collected 234840, Independent reflections 72290, R_{int} = 0.0981, R_{sigma} = 0.0925, Completeness to Θ = 24.835° 99.6 %, Data / Restraints / Parameters 72290/8557/4361, Goodness-of-fit on F2 1.101, Final R indexes, [I⩾2σ(I)] R1 = 0.1082, wR2 = 0.3203, Final R indexes, [all data] R1 = 0.2002, wR2 = 0.3822, Largest peak/hole [eÅ−3] 1.64/-0.83.

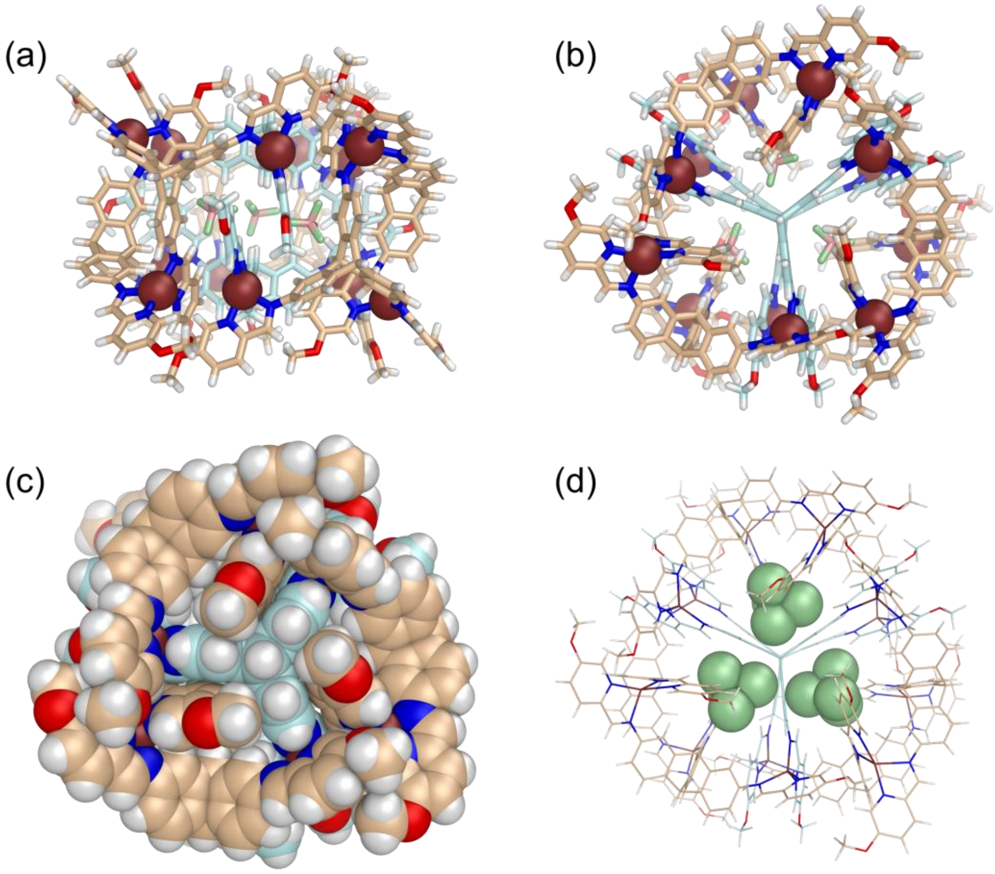

Figure S70. Views of $\mathbf{2}^{\mathbf{OMe}}$•$BF_4$ through (a) the C_2 axis and (b) the C_3 axis in stick representations and (d) through a C_3 axis in space-filling representations. (e) Illustrates of three bound BF_4^- anions that are in space-filling representations. Free counteranions, solvents and disorder are omitted for clarity. Hydrogen atoms are white, fluoride atoms are green, nitrogen atoms are blue, oxygen atoms are red, and copper atoms are ruby. The central ligands are coloured in pale cyan and the peripheral ligands are coloured in wheat.

14. References

1. Zhang, C.; Chen, C.-F. Synthesis and Structure of 2,6,14- and 2,7,14-Trisubstituted Triptycene Derivatives. *J. Org. Chem,* **2006**, *71*, 6626–6629.

2. Maglic, J. B.; Lavendomme, R. MoloVol: an easy-to-use program for analyzing cavities, volumes and surface areas of chemical structures. *J. Appl. Cryst.* **2022**, *55*, 1033–1044.

3. Allan, D. et al. A Novel Dual Air-Bearing Fixed-χ Diffractometer for Small-Molecule Single-Crystal X-ray Diffraction on Beamline I19 at Diamond Light Source. *Crystals*, **2017**, *7*, 336.

4. (a) Winter, G. xia2: an expert system for macromolecular crystallography data reduction. *J. Appl. Crystallogr.* **2010**, *43*, 186–190. (b) Winter, G. et al. DIALS: implementation and evaluation of a new integration package. *Acta Cryst*. **2018**, *D74*, 85–97.

5. Farrugia, L. WinGX and ORTEP for Windows: an update. *J. Appl. Crystallogr.* **2012**, *45*, 849–854.

6. Sheldrick, G. SHELXT-Integrated space-group and crystal-structure determination. *Acta. Cryst*. **2015**, *A71*, 3–8.

7. Sheldrick, G. M. Crystal structure refinement with SHELXL. *Acta. Cryst.* **2015**, *C71*, 3–8.

8. van der Sluis, P.; Spek, A. L. BYPASS: an effective method for the refinement of crystal structures containing disordered solvent regions. *Acta Cryst*. **1990**, *A46*, 194–201.

9. Spek, A. L. PLATON: A Multipurpose Crystallographic Tool. Utrecht University: Utrecht, The Netherlands, 2008.

10. Kabsch, W. *Acta. Cryst.* **2010**, *D66*, 125−132.

11. Evans, P. R. *Acta. Cryst.* **2006**, *D62*, 72−82.

12. Evans, P. R. *Acta. Cryst.* **2011**, *D67*, 282−292.

13. Winn M.D. et al., *Acta. Cryst.* **2011**, *D67*, 235-242.

14. Bricogne, G.; Blanc, E.; Brandle, M.; Flensburg, C.; Keller, P.; Paciorek, W.; Roversi, P.; Sharff, A.; Smart, O. S.; Vonrhein, C.; Womack, T. O., BUSTER. 2.11.2 ed.; Global Phasing Ltd.: Cambridge, United Kingdom, 2011.

15. Smart, O. S.; Womack, T. O., Grade Web Server. Global Phasing Ltd.: 2014
